# Diverse MarR bacterial regulators of auxin catabolism in the plant microbiome

**DOI:** 10.1038/s41564-022-01244-3

**Published:** 2022-10-20

**Authors:** Jonathan M. Conway, William G. Walton, Isai Salas-González, Theresa F. Law, Chloe A. Lindberg, Laura E. Crook, Suzanne M. Kosina, Connor R. Fitzpatrick, Adam D. Lietzan, Trent R. Northen, Corbin D. Jones, Omri M. Finkel, Matthew R. Redinbo, Jeffery L. Dangl

**Affiliations:** 1 Department of Biology, University of North Carolina at Chapel Hill, Chapel Hill, NC USA; 2 Howard Hughes Medical Institute, University of North Carolina at Chapel Hill, Chapel Hill, NC USA; 3 Department of Chemistry, University of North Carolina at Chapel Hill, Chapel Hill, NC USA; 4 Curriculum in Bioinformatics and Computational Biology, University of North Carolina at Chapel Hill, Chapel Hill, NC USA; 5 Environmental Genomics and Systems Biology, Lawrence Berkeley National Laboratory, Berkeley, CA USA; 6 Division of Oral and Craniofacial Health Sciences, Adams School of Dentistry, University of North Carolina at Chapel Hill, Chapel Hill, NC USA; 7 Joint Genome Institute, Lawrence Berkeley National Laboratory, Berkeley, CA USA; 8 Department of Genetics, University of North Carolina at Chapel Hill, Chapel Hill, NC USA; 9 Lineberger Comprehensive Cancer Center, University of North Carolina at Chapel Hill, Chapel Hill, NC USA; 10 Curriculum in Genetics and Molecular Biology, University of North Carolina at Chapel Hill, Chapel Hill, NC USA; 11 Department of Plant and Environmental Sciences, Institute of Life Sciences, The Hebrew University of Jerusalem, Jerusalem, Israel; 12 Department of Biochemistry and Biophysics, and the Integrative Program for Biological and Genome Sciences, University of North Carolina at Chapel Hill, Chapel Hill, NC USA; 13 Department of Microbiology and Immunology, University of North Carolina at Chapel Hill, Chapel Hill, NC USA; 14 Present Address: Department of Chemical and Biological Engineering, Princeton University, Princeton, NJ USA

**Keywords:** Systems biology, Microbial communities, Phylogenomics, Microbial ecology, X-ray crystallography

## Abstract

Chemical signalling in the plant microbiome can have drastic effects on microbial community structure, and on host growth and development. Previously, we demonstrated that the auxin metabolic signal interference performed by the bacterial genus *Variovorax* via an auxin degradation locus was essential for maintaining stereotypic root development in an ecologically relevant bacterial synthetic community. Here, we dissect the *Variovorax* auxin degradation locus to define the genes *iadDE* as necessary and sufficient for indole-3-acetic acid (IAA) degradation and signal interference. We determine the crystal structures and binding properties of the operon’s MarR-family repressor with IAA and other auxins. Auxin degradation operons were identified across the bacterial tree of life and we define two distinct types on the basis of gene content and metabolic products: *iac*-like and *iad*-like. The structures of MarRs from representatives of each auxin degradation operon type establish that each has distinct IAA-binding pockets. Comparison of representative IAA-degrading strains from diverse bacterial genera colonizing *Arabidopsis* plants show that while all degrade IAA, only strains containing *iad*-like auxin-degrading operons interfere with auxin signalling in a complex synthetic community context. This suggests that *iad*-like operon-containing bacterial strains, including *Variovorax* species, play a key ecological role in modulating auxins in the plant microbiome.

## Abstract (web summary)

Characterization of bacterial auxin degradation loci and their regulators reveals two distinct types across plant microbiome species, where only one, exemplified in *Variovorax* species, can interfere with root growth inhibition in a complex synthetic microbial community.

## Main

The many biochemical communication mechanisms that exist between plants and their microbiomes are only beginning to be elucidated, dissected and understood using modern omics, genetics and biochemical techniques^1–3^. These complex interactions affect plant host health, development and productivity, as well as the growth and persistence of microbiota at plant–microbe interfaces^4,5^. Auxins, a family of plant growth hormones produced and degraded by plants and bacteria, are an important biochemical signal used to mediate plant–microbe interactions^6,7^. Auxins, typified by indole-3-acetic acid (IAA) which is the most abundant auxin in plants, play critical roles in plant developmental and reproductive processes including cell division, root development, cell wall elongation, vascular patterning and flowering^8,9^. Auxins also interact with other plant hormone signalling pathways, including those regulated by ethylene, salicylic acid and jasmonic acid^10–12^. Members of the microbiota can produce and/or degrade auxins to modulate auxin levels and thus interactions with the plant host^6,7,13^. Recently, we identified an auxin-degrading locus in the bacterial genus *Variovorax* and demonstrated its crucial role in modulating microbiome community function via balancing the effects of microbe-derived auxins on plant root development^14^.

Auxin catabolism has been identified in many soil- and plant-derived bacterial isolates and consortia over the past 60 years^15–18^. However, only recently were the genes responsible for auxin catabolism identified in *Pseudomonas putida* 1290^19,20^. The indole-3-acetic acid catabolism (*iac*) locus in *P. putida* 1290 contains catabolism genes *iacABCDEFGHI* and a MarR-family transcriptional regulator *iacR*^19,20^. Similar *iac*-like loci have subsequently been identified and characterized to varying extents in *Enterobacter soli* LF7^21^, *Paraburkholderia phytofirmans* PsJN^22^, *Acinetobacter baumannii*^23^ and *Caballeronia glathei* DSM50014^24^. A distinct anaerobic IAA-degradation pathway to 2-aminobenzoyl-CoA was identified in *Aromatoleum evansii* strain KB740 and *Aromatoleum aromaticum* strain EbN1^25^. The genetic locus responsible for this anaerobic IAA degradation is termed the *iaa* locus (for indoleacetic acid) and contains genes *iaaABCDEFGHIJKLMPQR*^25^. Present knowledge of bacterial IAA catabolism involving aerobic *iac* loci and anaerobic *iaa* loci was recently reviewed^17^.

The auxin-degrading locus we identified previously^14^ in *Variovorax* lacks *iacA* and exhibits high sequence divergence from *iacCDEF*. A subsequent report of IAA degradation by two *Achromobacter* strains and genomic analysis describe a type of IAA degradation (*iad*) locus containing *iadABCDEFGHIJKLMNR*, which is distinct from the *P. putida* 1290 *iac* locus and the *A. evansii* KB740 *iaa* locus^26^. These *Achromobacter iad* genes have high sequence identity to the *Variovorax* IAA degradation locus, and thus we will adopt this *iad* nomenclature to describe the *Variovorax* locus (see Supplementary Table 1). The anaerobic IAA degradation *iaa* genes and pathway exhibit such high divergence from *iac* and *iad* aerobic IAA degradation that anaerobic IAA degradation will not be discussed further here.

The *iac* locus is regulated by IacR, a MarR-family transcriptional regulator. Aerobic IAA degradation loci contain either one or two MarR-family regulators; *P. phytofirmans* PsJN is an exception that contains a LysR regulator instead^22^. The MarR family of regulators is diverse^27^. Most characterized MarR-family regulators function as repressors by binding to an operator sequence upstream of the genes they control^28^. When a relevant ligand binds the MarR repressor, it releases from DNA, allowing gene expression. This was demonstrated in *E. soli* LF7 where IacR represses the *iac* locus IAA catabolism enzymes^21^. MarR regulators that function as transcriptional activators or have dual activator and repressor functionality have also been characterized^29–31^.

Here we dissect the *Variovorax iad* locus and its function in IAA degradation using both gain-of-function and loss-of-function genetics. We demonstrate that genes *iadDE*, which exhibit low similarity to *iac* locus genes *iacCD*, are minimally required to degrade IAA. We characterize the binding of IAA to MarR-family regulators from both *iac* and *iad* loci. We present 11 crystal structures of the *Variovorax* MarR-family regulator in unliganded and various ligand-bound states. We also provide crystal structures of MarR regulators from *P. putida*, *E. soli*, *Bradyrhizobium japonicum* and *A. baumannii*. We examine the role of MarR in regulation of the *Variovorax iad* locus. Using phylogenomics, we assess the diversity of IAA degradation loci and their MarR-family regulators and establish two distinct types of IAA-degrading locus (*iac*-like and *iad*-like) with clear gene content boundaries and sequence divergence. We demonstrate that strains from six genera relevant to the plant microbiome can degrade IAA in culture and that this capacity is maintained to revert root growth inhibition (hereafter, RGI) caused by IAA in mono-association with *Arabidopsis* seedlings. However, while all of these representative strains colonize *Arabidopsis* in a complex synthetic community context, only *Variovorax* and *Bradyrhizobium* provide the metabolic signal interference capacity to revert RGI caused by a diverse bacterial synthetic community in our experimental context^14^, suggesting that only bacteria containing the *iad* locus are able to perform auxin metabolic signal interference within a complex root microbiome.

## Results

### *Variovorax* genes are necessary and sufficient for IAA catabolism

**Fig. 1:**
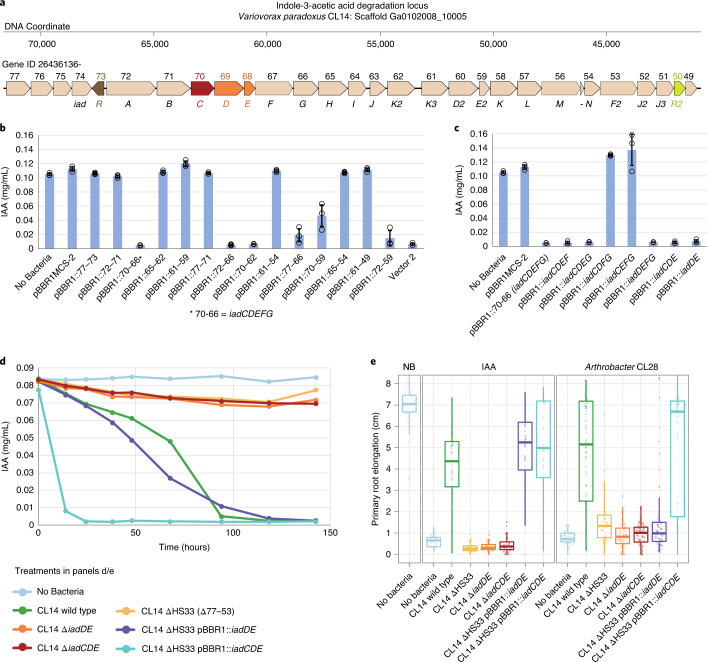
*V. paradoxus* CL14 *iadD* and *iadE* are necessary and sufficient for IAA degradation and metabolic signal interference on *Arabidopsis*. **a**, IAA degradation locus in *V. paradoxus* CL14. Genes are identified above by the last two digits of the IMG gene ID number 26436136## and below using the *iad* nomenclature from ref. ^26^. Genes of importance in this study are coloured: MarR transcriptional regulators MarR_73 (brown) and MarR_50 (yellow); the large and small subunit *iadD* and *iadE*, respectively, of an aromatic-ring hydroxylating dioxygenase (orange); and a ferredoxin subunit *iadC* (red). **b**,**c**, *iadD* and *iadE* are sufficient to complement IAA degradation in mutant *V. paradoxus* CL14 ΔHS33 (deletion of genes 77–53). Various portions of the *Variovorax* IAA degradation locus were cloned into broad host range vector pBBR1MCS-2 and screened in two rounds (see Extended Data Fig. 1). ‘Vector 2’ containing part of gene 72 through part of gene 61 was previously identified^14^. Final IAA concentration was measured after 3 d of culture in 2xYT medium (*n* = 3 biological replicates, bars and error bars are means ± 1 s.d.). **d**, *iadD* and *iadE* are necessary for IAA degradation by *V. paradoxus* CL14 and *iacC* enhances the IAA degradation rate. Strains were cultured in M9 medium containing succinate, and IAA and IAA concentration was measured over time (*n* = 3 biological replicates). **e**, *iadD* and *iadE* are necessary to prevent RGI caused by both IAA and *Arthrobacter* CL28. Complementation of the CL14 ΔHS33 mutant with pBBR1::*iadDE* is sufficient to prevent IAA-induced RGI, but pBBR1::*iadCDE* complementation is required to enable the *Variovorax* metabolic signal interference to prevent *Arthrobacter* CL28-caused RGI. Boxplot bold line, median primary root elongation; box edges, 25th and 75th percentiles; whiskers, 1.5× the interquartile range. Left to right *n* = 79, 48, 49, 48, 48, 48, 48, 48, 78, 78, 78, 78, 78, 78 and 79 biological replicates over two independent experiments for IAA and three independent experiments for CL28. Source data

**Extended Data Fig. 1:**
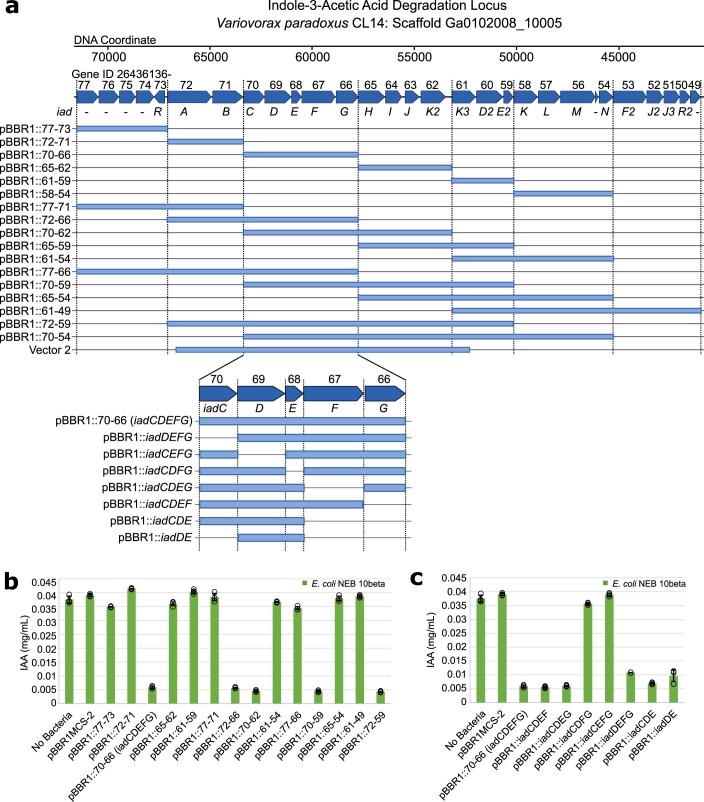
Vectors used to determine the minimal gene set of the *Variovorax paradoxus* CL14 IAA degradation locus required to degrade IAA. **a)** Vectors, named at left, contain the genes indicated by the blue bar in broad host range vector pBBR1MCS-2. For the vector insert, the *Variovorax iad* locus genes were cloned in the place of *lacZ* on pBBR1MCS-2 with the start codon of the first gene from the insert in place of the start codon of *lacZ*. The vector inserts contain the intergenic region following the last gene of the insert, ending at the start codon of the next sequential gene not contained in the insert. Vector 2, which contains part of gene 72 through part of gene 61, was identified previously^14^ from a library of random clones as being able to confer IAA degradation ability on *Escherichia coli* and *Acidovorax sp*. Root219. **b/c)** IAA degradation in *E. coli* NEB 10beta containing the indicated vectors after 3 days of culture in 2xYT medium supplemented with IAA (n = 3 biological replicates, bar represents the average, error bars represent one standard deviation from the average). Source data

Previously^14^, we defined the locus responsible for IAA degradation in *Variovorax paradoxus* CL14 (Fig. 1a). Mutant derivative *V. paradoxus* CL14 ΔHS33 lost the ability to degrade IAA or to revert RGI phenotypes caused by IAA or a commensal root bacterium, *Arthrobacter* CL28, on *Arabidopsis* seedlings^14^. To define the minimal genes required for IAA degradation, portions of the locus were cloned in place of *lacZ* on the broad host range plasmid pBBR1MCS-2^32^. We screened two vector sets (Extended Data Fig. 1a) for complementation of IAA degradation ability in *Escherichia coli* (Extended Data Fig. 1b,c) and the *V. paradoxus* CL14 ΔHS33 mutant background (Fig. 1b,c). We defined vector pBBR1::*iadDE* as the minimal vector necessary for IAA degradation in both the *V. paradoxus* CL14 ΔHS33 (Fig. 1c) and *E. coli* background (Extended Data Fig. 1c). Interestingly, however, the CL14 ΔHS33 pBBR1::*iadDE* strain degraded IAA slower than CL14 ΔHS33 pBBR1::*iadCDE*, which contains *iadC*, an annotated ferredoxin subunit (Fig. 1d). To test the necessity of these genes, we constructed knockout strains CL14 Δ*iadDE* and CL14 Δ*iadCDE*. Both lacked the ability to degrade IAA (Fig. 1d), demonstrating that *iadD* and *iadE* are necessary and sufficient for IAA degradation by *Variovorax*.

Next, we used a previously developed model system^14^ to test the ability of these *Variovorax* mutants to revert the RGI phenotype on *Arabidopsis* caused by IAA or *Arthrobacter* CL28 (Fig. 1e). In this system, sterile *Arabidopsis* seedlings are exposed to chemical or bacterial treatments whose influence over root development is assessed by measuring primary root elongation. Here, CL14 strains lacking the ability to degrade IAA (CL14 ΔHS33, CL14 Δ*iadDE* and CL14 Δ*iadCDE*) did not revert the RGI phenotype caused by either IAA or *Arthrobacter* CL28 (Fig. 1e). Both complementation strains, CL14 ΔHS33 pBBR1::*iadDE* and CL14 ΔHS33 pBBR1::*iadCDE*, were able to revert the IAA-induced RGI and these had slightly longer primary roots than seedlings treated with wild-ype CL14. However, only the CL14 ΔHS33 pBBR1::*iadCDE* strain containing all three genes was able to revert the *Arthrobacter* CL28-dependent RGI to CL14 wild-type levels. This suggests that the faster degradation rate in the CL14 ΔHS33 pBBR1::*iadCDE* strain may be essential for overcoming RGI caused by another bacterium, while slower degradation may be sufficient to revert RGI caused by one-time addition of IAA.

### Two MarRs regulate *Variovorax* IAA degradation

We recombinantly produced the two MarR proteins from the auxin degradation locus of *V. paradoxus* CL14, MarR_73 (IadR) and MarR_50 (IadR2), and employed isothermal titration calorimetry to determine their binding affinities for IAA, other auxins and related aromatic compounds. MarR_73 bound IAA with 0.41 ± 0.03 µM affinity, while no binding to MarR_50 was detected. Eleven additional auxin and aromatic ligands were examined, with ten exhibiting lower affinities for MarR_73 than that of IAA (Supplementary Table 2). Only 1-Naphthaleneacetic acid, a synthetic auxin, exhibited a higher affinity (0.33 ± 0.01 µM) than IAA.

**Fig. 2:**
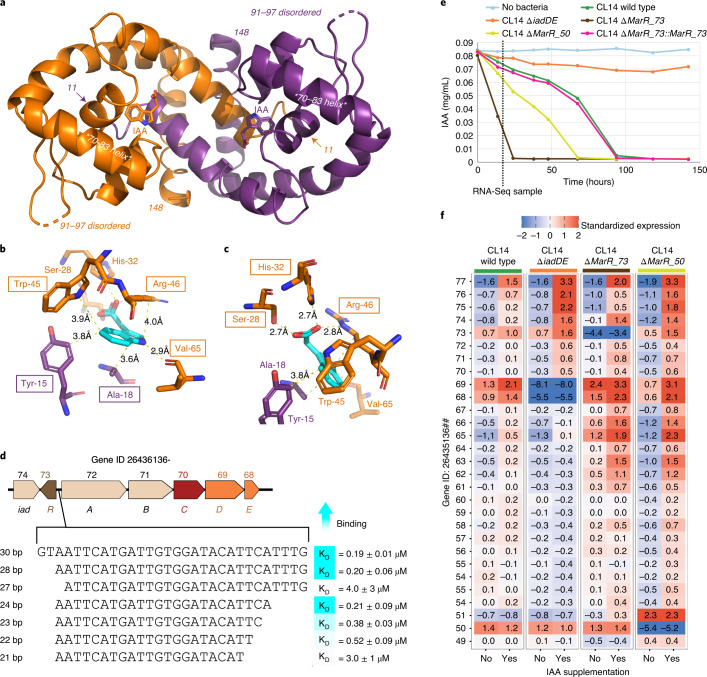
MarR_73 forms a dimer, binds to IAA and has a DNA binding site upstream of gene 72 to regulate the IAA degradation locus. **a**, The crystal structure of dimer MarR_73 at 1.3 Å with IAA bound to each MarR_73 monomer. The MarR_73 primary monomer is shown in orange, while the secondary monomer is shown in purple. All numbers correspond to residue position. Residues 11 and 148 are the first and last observed residues, respectively. The DNA binding helices (residues 70–83, labelled with asterisks) are not positioned in a manner conducive to binding DNA. For example, they are separated by 38 Å (intermonomer distance between the residue 77 Cα positions central to these helices), which is 10 Å further than that observed in complexes of MarRs and DNA (PMID 28124121). It has been shown that ligand binding shifts the MarR DNA binding helices and adjacent loops such that they are unable to bind the major groove of target DNA sequences^65^. **b**,**c**, Key contacts between MarR_73 and the IAA indole ring (**b**) and the IAA carboxylate (**c**). IAA is shown in teal. **d**, Identification of the 24 bp MarR_73 DNA binding sequence between genes 73 and 72 in the *Variovorax* IAA degradation locus. Binding affinities to DNA were determined using the MarR_73 S28A because of its weakened binding to IAA. *K*_D_ values are means± s.e.m. from two independent ITC measurements (see Supplementary Table 2 for full binding data). **e**, Deletion of MarR_73 or MarR_50 causes increased degradation of IAA. IAA was measured from cultures in M9 medium containing succinate and IAA (*n* = 3). The time of sampling for RNA-seq in **f** is shown as a dotted line. **f**, Transcriptomes of *V.* *paradoxus* CL14 wild type and deletion mutants reveal the role of MarR_73 and MarR_50 in the regulation of the IAA degradation locus (*n* = 3). MarR_73 represses the IAA degradation locus and is derepressed by IAA. Deletion of MarR_50 appears to amplify the upregulation of the IAA degradation locus in response to IAA. Source data

**Extended Data Fig. 2:**
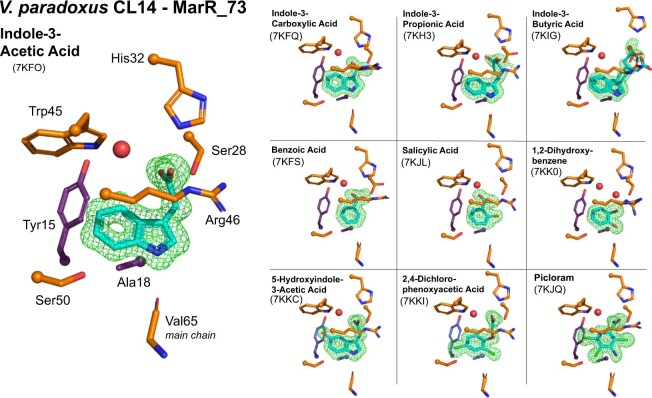
Simulated-annealing omit maps of ten *Variovorax paradoxus* CL14 MarR_73 crystal structures with distinct aromatic ligands bound. The amino acid groups contacting each ligand (cyan) co-crystallized with MarR_73 are shown, with orange residues from the primary monomer and purple from the secondary monomer. A water molecule localized to each binding site is rendered as a sphere in red. The electron density (green mesh, contoured at 3σ) for each ligand is generated from simulated-annealing (Fo-Fc) omit maps. PDB codes are shown in parentheses.

To understand the molecular basis of ligand binding by MarR_73, we determined its crystal structure complexed with IAA refined to 1.3 Å resolution (Fig. 2a, PDB code: 7KFO, Supplementary Tables 3 and 4). MarR_73 structures complexed with nine other auxins were also determined and refined to 1.35–1.6 Å resolution (Extended Data Fig. 2, and Supplementary Tables 3 and 4). The MarR_73 residues coordinating the indole ring of IAA are Tyr-15, Ala-18, Trp-45, Arg-46 and Val-65 (Fig. 2b), while those coordinating the IAA carboxylate are Ser-28, His-32 and Arg-46 (Fig. 2c). The MarR_73 structures resolved in complexes with other auxins revealed that the positions of ligand-contacting regions remained unchanged upon the binding of auxins, with the exceptions of the His-32 imidazole ring, which shifted by up to 2.9 Å, and the Arg-46 guanidinium group, which shifted by up to 4.2 Å (Extended Data Fig. 2). In both cases, these shifts accommodated differences in the carboxylate-containing substituent groups of the auxins examined.

**Extended Data Fig. 3:**
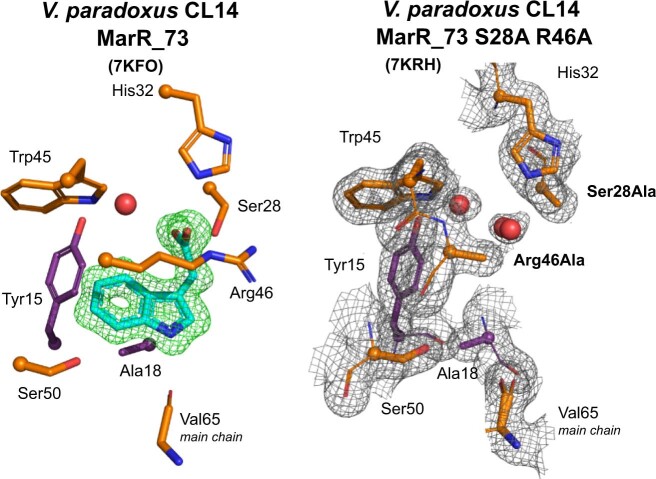
Electron density of the *Variovorax paradoxus* CL14 MarR_73 wild type compared to the S28A R46A mutant. Electron density (grey mesh, contoured at 3σ) from a simulated-annealing (2Fo-Fc) composite omit map reveals that the mutations (labeled in bold) that eliminate IAA binding do not alter the positioning of the remaining residues in the MarR_73 ligand-binding pocket. Water molecules are rendered as spheres in red. PDB codes are shown in parentheses.

Individual mutations to the carboxyl group-coordinating residues weakened or eliminated IAA binding (Supplementary Table 2). The 1.5 Å resolution crystal structure of the MarR_73 S28A R46A double mutant showed no ligand in the binding pocket (Extended Data Fig. 3) while maintaining the same structure as wild-type MarR_73, with a root-mean-square deviation in Cα positions of 0.18 Å. Using MarR_73 S28A protein with reduced ligand binding capacity (Supplementary Table 2), we identified a 24 bp double-stranded DNA oligo carrying a sequence located between *MarR_73 (iadR)* and gene 72 (*iadA*) that was capable of binding to this variant MarR_73 protein (Fig. 2d). These results show that MarR_73 is capable of binding to a DNA regulatory site ahead of the primary *iad* operon and appears to use auxin ligand binding to trigger DNA release and operon derepression.

Next, we constructed knockout mutant strains CL14 Δ*MarR_73* and CL14 Δ*MarR_50* to examine their regulatory roles. These MarR deletion strains were grown on minimal medium containing succinate and IAA (Fig. 2e), and both degrade IAA faster than CL14 wild type and a genetic control complementation strain CL14 Δ*Mar_73*::*MarR_73*. This suggests that both MarRs play a role in regulating aspects of IAA degradation, and loss of this regulation increases the rate of IAA degradation.

To further examine this, we performed RNA-seq on triplicate cultures of CL14 wild type, IAA degradation deficient strain CL14 Δ*iadDE*, and MarR mutants CL14 Δ*MarR_73* and CL14 Δ*MarR_50*. Standardized gene expression of the IAA degradation locus in these strains with and without IAA supplementation is shown in Fig. 2f. In CL14 wild type, the locus is upregulated in the presence of IAA. In the CL14 Δ*MarR_73* mutant, the genes downstream of gene 69 (*iadD*) are upregulated even in the absence of IAA. This demonstrates the repressive regulatory role of MarR_73 in the absence of IAA. With the addition of IAA in the CL14 Δ*MarR_73* mutant, genes in the locus are induced above the upregulated expression levels without IAA, suggesting that there may be additional layers of regulation controlled by IAA addition independent of the regulation through MarR_73. In the CL14 Δ*MarR_50* mutant, only gene 51 appears to be highly upregulated independent of the presence of IAA, while the rest of the loci appear to be repressed in the absence of IAA and activated in the presence of IAA, as in the wild type.

These data (Fig. 2) demonstrate that MarR_73 functions as a repressor of this locus: in the absence of IAA, MarR_73 binds to a DNA site between *MarR_73 (iadR)* and *iadA* to repress expression of the IAA degradation genes. When IAA is present, it binds to MarR_73, releasing MarR_73 from the DNA site to allow expression of the IAA degradation genes downstream. Because neither a ligand nor a DNA binding site was identified for MarR_50, and RNA-seq only identified MarR_50 as potentially regulating the non-essential gene 51, its precise role in the regulation of IAA degradation remains unclear.

### Phylogenomics defines two bacterial auxin-degrading loci

**Fig. 3:**
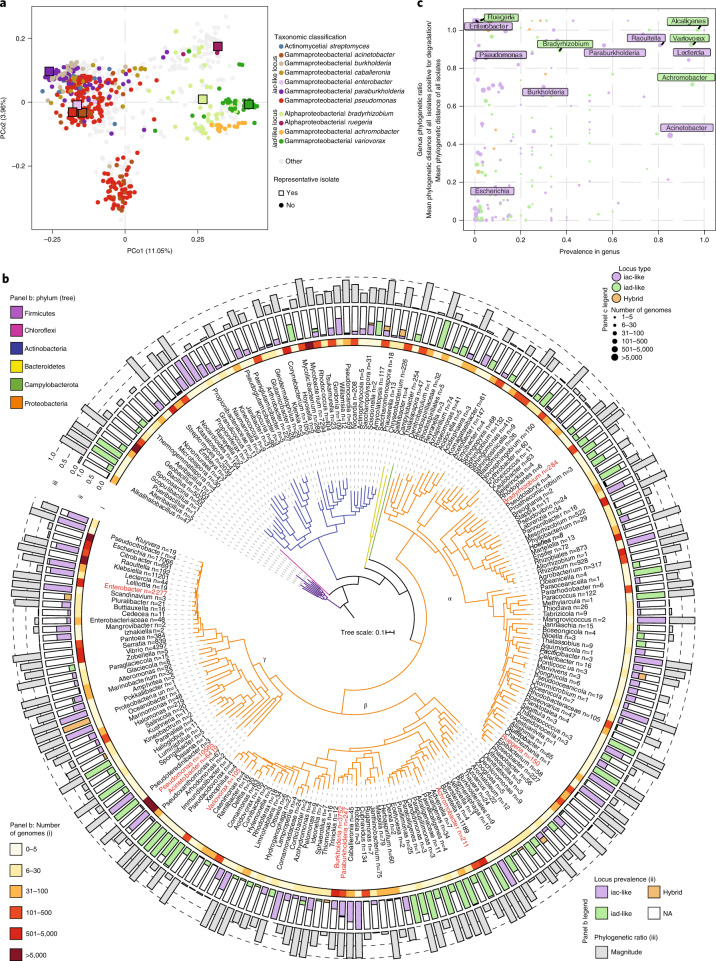
Phylogenomics delineates two distinct branches of IAA degradation loci: *iac*-like and *iad*-like. **a**, PCoA analysis of the gene repertoire across loci harbouring adjacent *iacC* and *iacD* homologue genes. Across the first principal coordinate, two distinct clouds are delineated. Each dot within the scatterplot represents a locus. The auxin-degrading loci obtained from previously validated operons are highlighted within the scatterplot. **b**, Phylogenetic tree of the 221 genera with at least one strain harbouring an auxin-degrading operon. Each tip in the tree represents a genus, the branches of the tree are coloured on the basis of the phylum classification of that given tip. Within the name of each genus, the total numbers of isolates in the RefSeq 202 database are highlighted. In addition, three outer rings (i, ii, iii) are shown. Ring (i) is coloured on the basis of the total number of isolates for each genus within the RefSeq 202 database. Ring (ii) represents the prevalence of the auxin-degrading operon across the isolates in that genus and corresponds to the ratio between the number of isolates within the genus harbouring an auxin-degrading operon and the total number of isolates within the genus. Ring (iii) represents the evenness of the distribution of the auxin-degrading operon across a genus taking into account the topology of an inferred phylogenetic tree for that given genus. The evenness of distribution is estimated via the phylogenetic ratio that is defined as the ratio between the mean phylogenetic distance of all isolates within the genus harbouring an auxin-degrading locus and the mean phylogenetic distance of all isolates within the genus. Genera highlighted in red have at least one strain utilized in this study (see Fig. 6). **c**, Scatterplot showing the phylogenetic ratio of the 221 aforementioned genera vs auxin-degrading locus prevalence in each genus. The size of each dot corresponds to the total number of genomes within that genus. The names of representative genera are overlayed in the scatterplot. Source data

**Extended Data Fig. 4:**
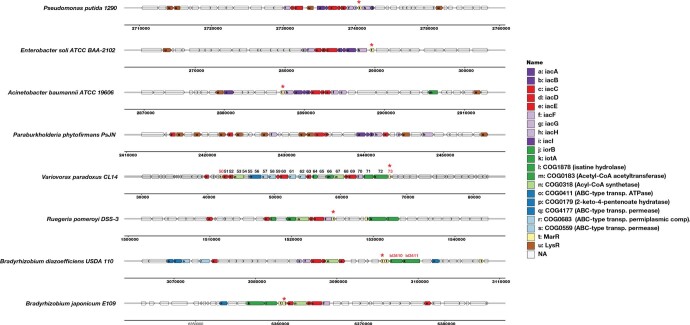
Homologous genes across auxin degradation loci in known auxin degrading bacteria. Labels a-i: homologs of *Pseudomonas putida* 1290 *iac* genes; j & k: indolepyruvate ferredoxin oxidoreductase beta subunit (*iorB*) and alpha subunit (*iotA*); l-s: homolog genes in the *Variovorax* and *Bradyrhizobium iad* loci; t: MarR family transcriptional regulator; u: LysR family transcriptional regulator. Source data

All previously characterized aerobic auxin catabolic operons contain homologous genes with distinct levels of sequence dissimilarity to the *iacCDEF* genes described in *P. putida* 1290^14,17^. Despite this, the gene neighbourhoods between these experimentally validated operons can be divided in two types on the basis of gene prevalence among them (Extended Data Fig. 4). To further delineate this subdivision of operons, we performed a de novo profiling of loci containing adjacent genes homologous to the *iacCD* genes via the scanning of ~180,000 bacterial genomes available at the RefSeq database. This analysis resulted in a neighbourhood gene content matrix tabulated using the distribution of Cluster of Orthologous Groups (COG) across all loci harbouring potential IAA-degrading genes. Using principal coordinate analysis (PCoA), we identified two major types of locus exhibiting dissimilar gene content (Fig. 3a), which correlated perfectly with the separation of previously experimentally validated *iac* and *iad* auxin-degrading loci, supporting the existence of two major types of auxin-degrading locus within the bacterial kingdom: *iac*-like and *iad*-like.

To further explore the distribution of the two major types of auxin-degrading locus, we performed a conservative profile scanning using type-specific marker genes (Extended Data Fig. 4) across the ~180,000 RefSeq isolates. The genomic potential for auxin degradation is distributed across five Phyla (Firmicutes, Actinobacteria, Bacteroidetes, Chloroflexi and Proteobacteria) and 221 genera in the bacterial kingdom (Fig. 3b). Additionally, we delineated the evolutionary trajectories of the loci across the bacterial tree of life by estimating metrics of IAA-degrading locus prevalence within each of the 221 bacterial genera. The prevalence and evenness of distribution of the auxin-degrading loci across the 221 genera differed dramatically, ranging from genera that contained a single strain harbouring an auxin-degrading locus to genera in which the prevalence of the locus was a core trait (Fig. 3b,c). Among the genomes harbouring *iad*-like loci, only three genera with substantial isolate representation in the database (*n* > 30)—*Variovorax*, *Alcaligenes* and *Achromobacter*—exhibited a high auxin-degrading locus prevalence (average prevalence >0.95) (Fig. 3b,c).

### *iad* loci degrade IAA through isatin to anthranilic acid

**Fig. 4:**
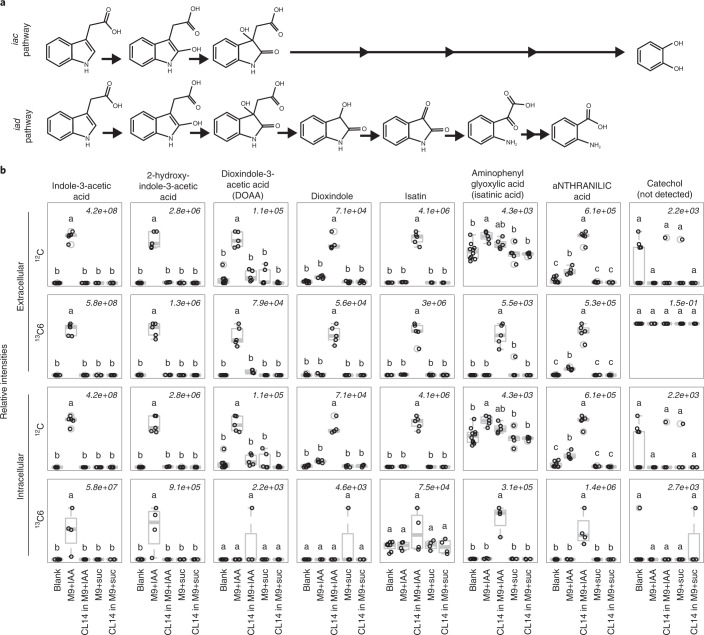
Metabolomics identifies the IAA degradation pathway in *V. paradoxus* CL14. **a**, The two known aerobic bacterial IAA degradation pathways. Top: the IAA degradation pathway used by *iac-*like loci from IAA through DOAA to catechol^17,20,22,24^. Bottom: the IAA degradation pathway used by *iad*-like loci, previously identified in *B. japonicum*^34^ and shown here for *V. paradoxus* CL14 from IAA through isatin to anthranilic acid. **b**, Metabolomics analysis of IAA degradation in *V. paradoxus* CL14. Intracellular and extracellular extractions from cell pellet and culture supernatant, respectively, of *V. paradoxus* CL14 grown in M9 minimal medium supplemented with either IAA, ^13^C6-IAA (with the 6 carbons of the benzene ring of the indole labelled), or succinic acid (suc), were analysed by LC–MS/MS to evaluate production of IAA degradation products. Succinate-supplemented M9 was used as a control for de novo synthesis of metabolites. ^13^C6-IAA supplementation was used to confirm intermediates generated during the degradation in ^12^C IAA-supplemented samples. Media controls were incubated but not inoculated with bacteria. Blank extraction controls were prepared alongside samples and used to indicate background signal. The maximum signal of the compound’s predominant ion is indicated at the top right of each boxplot (centre, median; box, 25th to 75th percentile; hinge, 1.58× interquartile range/sqrt(*n*)). Letters above boxplots indicate significant differences by one-way analysis of variance (ANOVA) (Tukey-HSD, alpha = 0.05, *n* of extraction controls = 10, *n* of supernatants = 5, *n* of pellets = 4). The full metabolomics identification table can be found in Supplementary Table 6.

The two known aerobic bacterial IAA degradation pathways, *iac* and *iad*, are shown in Fig. 4a. In *iac* locus-containing bacteria, IacA converts IAA to 2-hydroxy-indole-3-acetic acid, which is then converted to dioxindole-3-acetic acid (DOAA) by IacE, as has been shown in *P. putida* 1290^20^ and *C. glathei* DSM50014^24^. The genetic requirement of *iacA* in conversion of IAA was shown in *A. baumannii*^33^ and *E. soli* LF7^21^. Studies of the *iac*-like locus in *P. phytofirmans* PsJN^22^ suggest that IacG works with IacA, and IacB works with IacE in the initial two steps of IAA degradation. Conversion of DOAA to catechol is thought to involve multiple steps but chemical intermediates have not been determined. This conversion requires *iacC* in *P. putida* 1290^20^, and involves *iacCDF* and possibly *iacI* in *P. phytofirmans* PsJN^22^.

**Extended Data Fig. 5:**
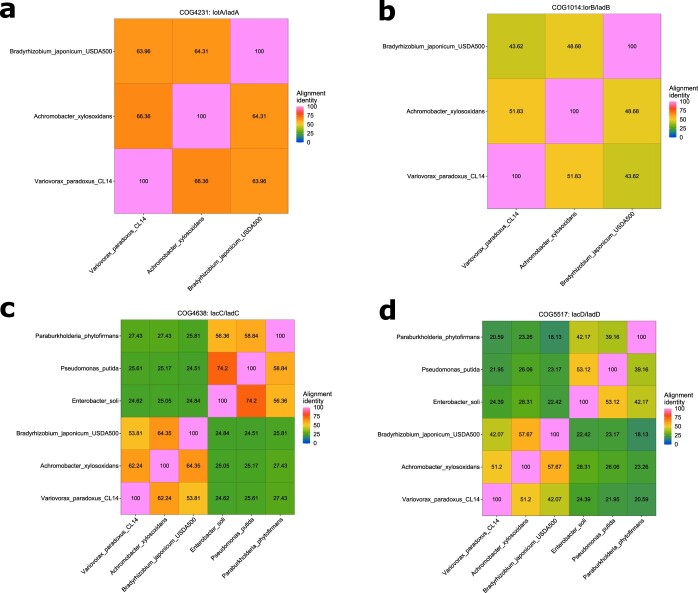
Amino acid sequence similarity across COGs found in *iac* and *iad* loci from key strains. **a)** COG4231 IotA/IadA **b)** COG1014 IorB/IadB **c)** COG4638 IacC/IadC **d)** COG5517 IacD/IadD. Percentage amino acid sequence similarity is given. Source data

In contrast, *B. japonicum* USDA110, which contains an *iad* locus (Extended Data Fig. 4) with high sequence identity to other *iad* loci (Extended Data Fig. 5, and Supplementary Tables 1 and 5), degrades IAA through DOAA to isatin and then on to anthranilic acid^34^ (Fig. 4a). We used metabolomics to analyse both intracellular and extracellular metabolites from *V. paradoxus* CL14 grown on either IAA, ^13^C6-labelled IAA, or succinate (Fig. 4b, full identification table in Supplementary Table 6) to demonstrate that *V. paradoxus* CL14 degrades IAA through the same pathway as *B. japonicum*. IAA, 2-hydroxyindole-3-acetic acid and DOAA were all detected in the media controls (possibly due to abiotic degradation) and are completely consumed by *V. paradoxus* CL14. This does not preclude production of these intermediates by CL14, as their presence may be transient due to rapid consumption in downstream reactions of the pathway. The intermediates dioxindole, isatin and isatinic acid, and the product anthranilic acid were all detected and thus produced in the bacterial cultures. Importantly, catechol, the main product of the *iac* IAA degradation, was not detected in any of the *V. paradoxus* CL14 samples.

### MarRs from IAA-degrading bacteria cluster into three groups

**Fig. 5:**
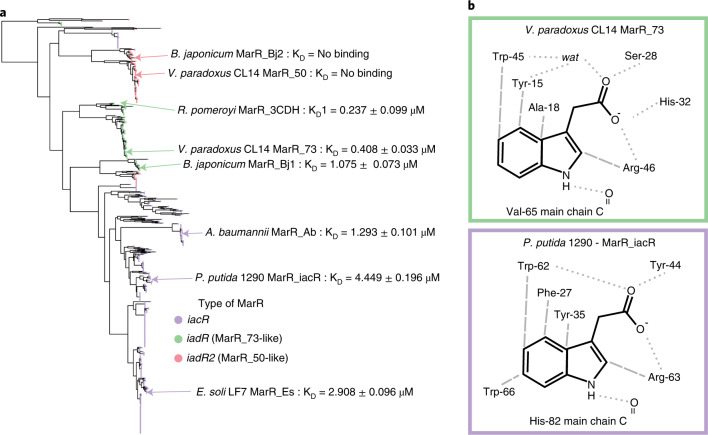
MarR-transcriptional regulators associated with IAA degradation loci form *iac*-like (IacR) and *iad*-like groups (IadR and IadR2) which have distinct IAA-binding pockets. **a**, Phylogenetic tree of protein sequences for MarR proteins associated with auxin-degrading operons showing separation of the *iacR* (purple), *iadR* (MarR_73-like, green) and *iadR2* (MarR_50-like, red) branches. Representative MarRs are indicated. **b**, Key IAA-binding contacts in *V. paradoxus* CL14 MarR_73 and *P. putida* 1290 MarR_iacR as representatives of the *iadR* and *iacR* groups, respectively. Hydrogen bonding interactions between protein groups and ligand are indicated with grey dotted lines (for example, *P. putida* 1290 MarR_iacR Tyr44, which replaces Ser-28 in *V. paradous* MarR_73, is positioned 2.6 Å from IAA carboxylate, the same distances observed between Ser-28 and this group in the MarR_73 complex) and non-hydrogen bonds with grey dashed lines. See Extended Data Fig. 6 for crystal structures of all MarRs from this study and Extended Data Fig. 7 for IAA contacts in additional MarR proteins. See Extended Data Table 1 for IAA-binding data. Source data

**Extended Data Fig. 6:**
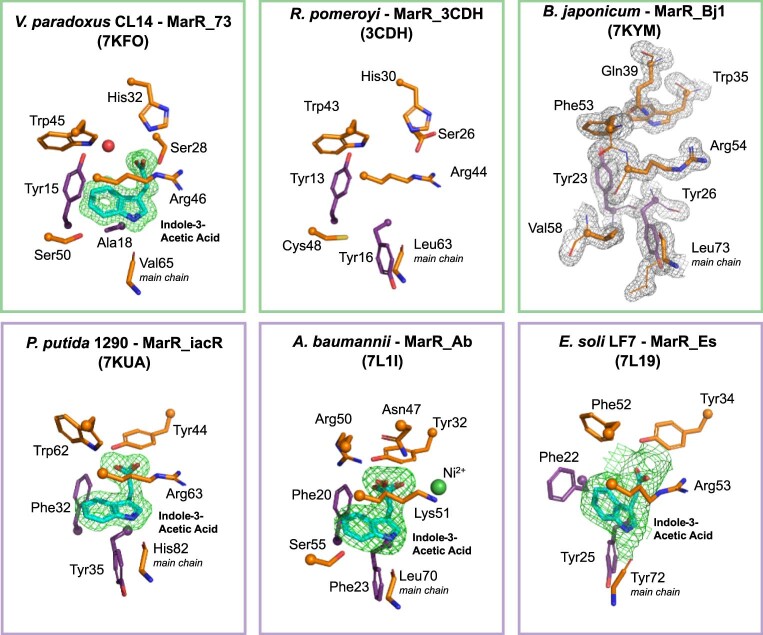
Ligand-binding pockets of MarR-family proteins. Binding contacts between MarR receptors and indole-3-acetic acid (IAA) are shown, with orange residues from the primary monomer and purple from the secondary monomer. Electron density (green mesh, contoured at 3σ) is generated from simulated-annealing (Fo-Fc) omit maps. A water molecule is shown in a red sphere. Electron density (grey mesh, contoured at 3σ) for *B. japonicum* MarR_Bj1 was generated from a simulated-annealing (2Fo-Fc) composite omit map. PDB codes for all structures are shown in parentheses. The structure of *R. pomeroyi* MarR_3CDH was solved previously (see doi: 10.2210/pdb3cdh/pdb).

**Extended Data Fig. 7:**
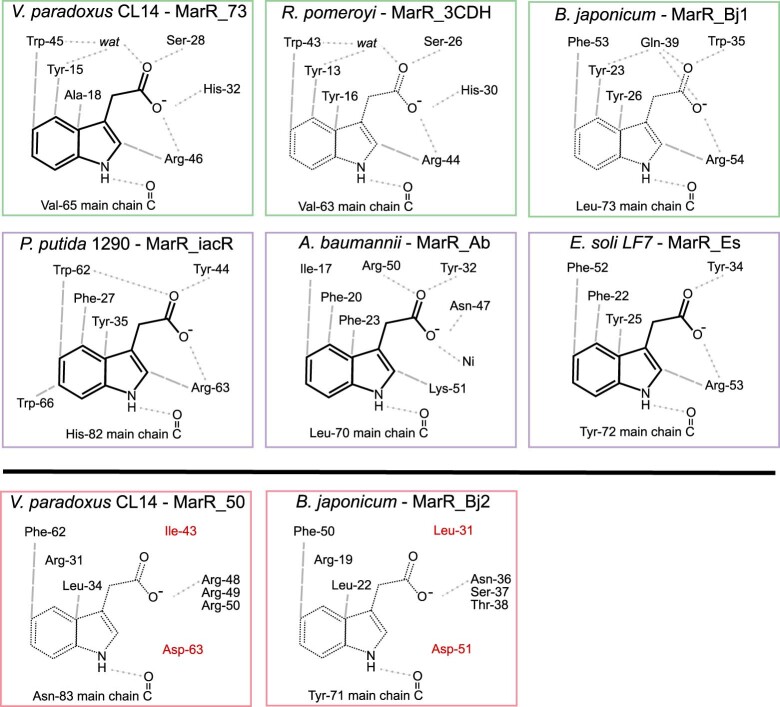
Key IAA-binding residues Conserved in MarR-family proteins. At top, five crystal structures reported here along with the previously reported structure 3CDH; four of these structures contain bound indole-3-acetic acid (IAA, chemical structure shown as solid lines) and two were in unliganded states (IAA chemical structure shown as dashed). Hydrogen bonding interactions between protein groups and ligand are indicated with grey dotted lines, and non-hydrogen bonds with gray dashed lines. Outline colors correspond to *Variovorax* MarR_73-like (green), *iac*-like (purple), and *Variovorax* MarR_50-like (red) MarR types. The three top *Variovorax* MarR_73-like (green) proteins cluster together and exhibit highly similar ligand-binding residues. The three *iac*-like (purple) proteins, at middle, also cluster together and employ residues distinct from those at top, while maintaining similar contacts to IAA. The two *Variovorax* MarR_50-like (red) proteins, at bottom, do not have representative crystal structures; however, analysis of amino acid positions equivalent in sequence reveal that key contacting residues in the IAA-binding proteins at top are not conserved, specifically those highlighted in red.

Given the two main clades of auxin-degrading loci and their different metabolic pathways, we examined more closely the MarRs from a diverse set of IAA-degrading bacteria. MarR proteins were produced from three *iad* and three *iac* loci and their binding affinities for IAA were measured (Extended Data Table 1). The strongest binding to IAA was observed for the IadR MarRs from *iad* loci, with MarR_3CDH from *Ruegeria pomeroyi* having the highest affinity (*K*_D_ of 0.237 ± 0.099 µM). As with the *V. paradoxus* MarR_50, the secondary IadR2 MarR from *B. japonicum* (MarR_Bj2) did not bind to IAA. On the basis of the phylogeny of the MarRs from the auxin-degrading operons identified in the phylogenomic analysis (Fig. 3), these MarR proteins cluster into *iacR, iadR and iadR2* groups (Fig. 5a). Crystal structures for the IAA-binding IacR MarRs from *P. putida, E. soli* and *A. baumannii* and IadR MarR from *B. japonicum* were determined and refined to 1.5–2.8 Å resolution (Extended Data Fig. 6, and Supplementary Tables 3 and 4). The *Variovorax* IAA-binding MarR proteins and the previously deposited structure of *R. pomeroyi* MarR_3CDH (PDB code 3CDH) were compared for their IAA-contacting residues and contrasted with MarRs that do not bind IAA (Extended Data Fig. 7). The contacts between MarR and IAA ligand are typified for the *iadR* group by MarR_73 and the *iacR* group by *P. putida* 1290 MarR_iacR (Fig. 5b). By contrast, MarRs that do not bind IAA lack a similar network of residues capable of coordinating the carboxylic acid group of IAA, such as those predicted for *Variovorax* MarR_50 and *B. japonicum* MarR_Bj2 (Extended Data Fig. 7). These observations define key molecular contacts involved in IAA binding by MarR orthologues responsive to this ligand.

### Bacteria encoding the *iad*-like locus revert RGI

**Fig. 6:**
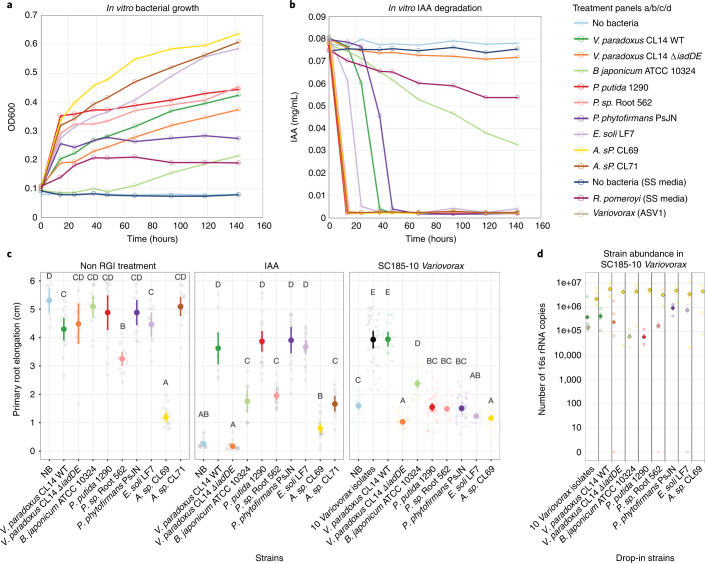
Strains encoding *iad*-like IAA degradation loci perform the metabolic signal interference required to prevent RGI caused by a 175-member synthetic microbiome community on *Arabidopsis* roots. **a**,**b**, Growth as measured by OD_600_ (**a**) (*n* = 3) and IAA degradation by diverse IAA-degrading strains in M9 medium with amino acids and IAA (**b**) (*n* = 3). *R. pomeroyi* was grown in identical medium supplemented with 2% (w/v) sea salts (SS media). **c**, Measurement of primary root elongation confirms that IAA-degrading strains can prevent IAA-induced RGI, but only representatives from *Variovorax* and *Bradyrhizobium* (both carrying *iad*-like loci) are able to prevent RGI induced by a 175-member synthetic community (SC185-10V, a 185-member synthetic community used previously^14^ minus its 10 *Variovorax* members). Mean primary root elongation is shown as a bold circle with 95% confidence interval. Significance was determined via the fitting of an ANOVA model within each panel with the design primary root elongation ~ Bacteria; letters above each treatment represent the compact letter of a Tukey post hoc test. Left to right: no RGI treatment: *n* = 53, 18, 17, 17, 24, 15, 15, 12 and 15, 19 biological replicates; IAA: *n* = 19, 13, 20, 11, 22, 20, 17, 20, 20 and13 biological replicates; SC185-10V: *n* = 41, 36, 33, 40, 44, 45, 39, 50, 39 and 44 biological replicates over 2 independent experiments. **d**, 16S absolute abundance measurement (*n* = 5 open circles, median shown as diamond with black contour) confirms colonization and persistence of all IAA-degrading strains in the SC185 minus 10 *Variovorax* synthetic community even for the *iac*-like loci-containing bacteria that do not revert the RGI phenotype in *Arabidopsis* roots shown in **c**. *Variovorax* ASV1 (grey) corresponds to *Variovorax* 16S amplicon sequence variants of *Variovorax* strains in the ‘10 *Variovorax* isolate’ treatment that are distinct from the *Variovorax* CL14 ASV (dark green). *Acinetobacter* sp. CL69 is present in SC185-10V and is thus found in all samples. Each panel in the figure focuses on a specific drop-in treatment, with the respective inoculated strains highlighted on the *x* axis. Source data

To compare the functions of different IAA-degrading genera, strains from different genera were grown in medium supplemented with IAA while tracking growth (Fig. 6a) and IAA concentration (Fig. 6b). All strains tested with IAA degradation loci were able to degrade IAA at varying rates; most strains degraded IAA completely within 48 h. Next, we tested the ability of these strains to degrade IAA and affect *Arabidopsis* root development. All strains, except the marine bacterium *R. pomeroyi* which requires high salt conditions not amenable to *Arabidopsis* growth, were applied either alone, with 1 µM IAA, or with an RGI-inducing synthetic community ‘SC185-10 *Variovorax*’ (a 185-member synthetic community minus the 10 *Variovorax* members defined previously^14^) to *Arabidopsis* seedlings. Primary root elongation was assessed as a measure of the ability of the IAA-degrading strains to revert RGI caused by either IAA or the synthetic community. Nearly all the IAA-degrading strains had little or no effect on the *Arabidopsis* primary root elongation when applied alone, and only *Acinetobacter* sp. CL69 caused RGI (Fig. 6c). *Arabidopsis* with IAA alone exhibited the expected severe RGI phenotype. This RGI phenotype was reverted by all the IAA-degrading bacteria that do not themselves cause RGI (*Acinetobacter* sp. CL69), presumably by consuming the IAA supplemented in the media. Finally, we tested these bacteria in a complex synthetic community (SynCom) context. Surprisingly, only *Variovorax* (a mix of 10 *Variovorax* strains or *V. paradoxus* CL14 alone) and *B. japonicum* were able to revert the RGI caused by the SC185-10V SynCom. These strains have an *iad*-like IAA-degrading locus. None of the bacteria containing an *iac*-like locus were able to revert the RGI phenotype caused by the SC185-10V SynCom. To rule out that this was due to lack of colonization or persistence in the synthetic community by these IAA degraders, 16S ribosomal RNA-guided bacterial absolute abundance was analysed at the end of the colonization experiment. Within the SC185-10V SynCom, all inoculated *iac*-like loci-containing bacteria exhibited quantifiable 16S rRNA signal within the community, but the RGI reversion did not correlate with the strain’s abundance (Fig. 6d).

## Discussion

The capacity of bacteria to degrade IAA and other auxins exists in a diverse array of bacteria^19–22,24,26,34^. The most well-studied of these is *P. putida* 1290 where the genes responsible (*iacABCDEFGHIR*, collectively the *iac* operon) were first identified^19^. Only recently was an auxin degradation locus with high sequence divergence from this canonical *iac* operon identified in *Variovorax* and shown to play a critical role in auxin metabolic signal interference to maintain stereotypic plant root development^14^. Here we demonstrated that these two are distinct types of bacterial auxin degradation locus: *iac*-like exemplified by *P. putida* 1290, and *iad*-like exemplified by *V. paradoxus* CL14. Metabolomics in previous studies of *B. japonicum*^34^*, P. phytofirmans* PsJN^22^ and *P. putida* 1290^19,20^, and performed here on *V. paradoxus* CL14, showed that *iac*-like loci degrade IAA to catechol, while *iad*-like loci degrade IAA to anthranilic acid. Further, by examining the binding of MarR-family regulators from both locus types and solving crystal structures for five of these MarRs, we demonstrated that IadR MarRs (for example, *Variovorax* MarR_73) have stronger binding affinity for IAA than their *iac*-like counterparts, probably caused by optimized coordination of the IAA carboxyl group in their MarR binding pocket. This may explain the ability of Iad-expressing strains to more readily catabolize auxin and contribute to their RGI-reversion phenotype. The *iad*-like loci also contain a second MarR (IadR2, for example, *Variovorax* MarR_50), for which we did not identify a ligand. The increased IAA degradation in deletion strain *V. paradoxus* CL14 Δ*MarR_50* suggests that this second non-IAA-binding MarR plays some role in IAA degradation regulation, but this requires additional investigation to elucidate.

In addition to our work understanding the structure and function of the MarRs from these loci, we used gain- and loss-of-function genetics in *V. paradoxus* CL14 to better understand the genes required for IAA degradation in the *iad*-like auxin degradation locus. We defined *iadD* and *iadE*, weak homologues of *iacC* and *iacD*, that encode the alpha- and beta subunit of an aromatic-ring hydroxylating dioxygenase, respectively, as necessary and sufficient for IAA degradation in *V. paradoxus* CL14. Gene 70 (*iadC*) of the CL14 operon, which encodes a ferredoxin subunit, speeds up degradation of IAA and was required along with *iadD* and *iadE* to complement CL14 ΔHS33 to perform metabolic signal interference in duo-association with *Arthrobacter* CL28 in *Arabidopsis*. Perhaps other ferredoxin subunits in the CL14 genome can compensate for the loss of IadC when it is absent, but to achieve maximal auxin degradation and perform the necessary signal interference in duo-association with an auxin-producing strain, *iadC* appears to be required.

Finally, we tested the auxin degradation capacity of *iad*-like and *iac*-like locus-containing strains in parallel to establish their ability to degrade IAA in vitro and revert RGI phenotypes both in mono-association with *Arabidopsis* where IAA is applied and in our 185-10V synthetic community model system. All tested strains degraded IAA in culture and reverted RGI caused by a one-time addition of IAA to *Arabidopsis* in mono-association. Yet, when combined in the SC185-10V synthetic community, only strains containing *iad*-like operons reverted RGI caused by the synthetic community. Importantly, this discrepancy is not due to insufficient colonization or persistence of *iac*-like strains. It is not known what auxins are produced by the synthetic community, their concentration, or localization. It is possible that *iad*-like operons may be associated with auxin transporters or other accessory genes which make these strains more effective in a community context. Co-localization of these strains within the root system may also play a role, such that auxin-producing strains may more readily associate with anthranilic acid-producing *iad* locus-containing strains rather than *iac* locus-containing strains. Ultimately, we demonstrate the importance of strains containing *iad*-like IAA-degrading loci for maintaining stereotypic root development in a complex ecological context, substantially expanding on the ecological ramifications of our previous study^14^. Our elucidation of the structures and functions of auxin-binding MarR regulators of auxin degradation loci of both *iac*-like and *iad*-like types and definition of the required gene for IAA degradation in *Variovorax* enable further study of this model system and the development of strains with auxin signal interference properties appropriate for application in real-world microbiome systems.

## Methods

### Bacterial strains and media

A collection of 185 genome-sequenced bacterial isolates, described previously^14^, was utilized to assemble the synthetic community used in this work. These isolates were obtained from surface-sterilized Brassicaceae roots, primarily *Arabidopsis thaliana*, grown in two soils from North Carolina, USA^35^. This isolate collection includes strains *V. paradoxus* CL14, *Arthrobacter* CL28, *Acinetobacter* CL69 and *Acinetobacter* CL71, which are also used in this work in individual strain contexts. *V. paradoxus* CL14 ΔHS33, which has a clean deletion of genes with gene ID 2643613677 through 2643613653 was constructed previously^14^ and used here. Additional strains were obtained from the American Type Culture Collection (ATCC): *E. soli* LF7 (ATCC BAA-2102), *R. pomeroyi* (ATCC 700808) and *B. japonicum* (ATCC 10324). *P. phytofirmans* PsJN (DSMZ 17436) was obtained from the DSMZ-German Collection of Microorganisms and Cell Cultures. *P. putida* strain 1290 was generously provided by Johan Leveau (University of California Davis). *Pseudomonas* strain Root 562 was generously provided by Paul Schulze-Lefert (Max-Planck-Gesellschaft). All bacteria, with exceptions noted below, were routinely grown on LB agar plates (10 g l^−1^ tryptone, 5 g l^−1^ yeast extract, 10 g l^−1^ NaCl, 1.5% (w/v) agar) and in 2xYT liquid medium (16 g l^−1^ tryptone, 10 g l^−1^ yeast extract, 5 g l^−1^ NaCl) at 28 °C. The 175-member (185-member minus 10 *Variovorax* strains) synthetic community (SC185-10V) was grown on KB medium as was done previously to culture this synthetic community^14^. *B. japonicum* (ATCC 10324) was routinely grown on liquid and solidified YM medium (1 g l^−1^ yeast extract, 10 g l^−1^ mannitol, 0.5 g l^−1^ dipotassium phosphate, 0.2 g l^−1^ magnesium sulfate, 0.1 g l^−1^ NaCl, 1 g l^−1^ CaCO_4_, pH 6.8, solidified with 1.5% agar as necessary) at 28 °C. *R. pomeroyi* (ATCC 700808) was routinely grown on liquid and solidified LB medium supplemented with 2% sea salt (Millipore Sigma S9883) and solidified with 1.5% (w/v) agar as necessary. M9 base medium was formulated using 1x M9 minimal salts medium (Sigma M6030) supplemented with 2 mM MgSO_4_, 0.1 mM CaCl_2_ and 10 µM FeSO_4_. A carbon source or sources were added to this M9 base medium to support bacterial growth. Unique strains constructed in this study are available upon request.

### Bacterial 16S rRNA sequencing

Bacterial colonization of *Arabidopsis* roots was assessed using a method similar to the previous study^14^. Roots from 8–10 plants were collected into sterilized 2 ml tubes containing three 4 mm glass beads and root fresh weight in each tube was obtained. Five such samples were collected for each bacterial treatment. The roots were washed three times with sterile distilled water and stored at −80 °C until further processing. The roots were then lyophilised for 48 h using a Labconco freeze-dry system and pulverized using an MPBio tissue homogenizer. DNA was extracted from the root samples and bacterial cell pellets saved from the bacteria for input into the experiment using the DNeasy PowerSoil HTP 96 kit (Qiagen) according to manufacturer instructions. The V3-V4 region of the bacterial 16S rRNA gene was amplified and sequenced as previously described^14^.

### 16S amplicon sequence data processing

The 16S sequencing data from synthetic community experiments were processed as previously described^14^. Briefly, usable read output from MT-Toolbox^36^ (reads with 100% primer sequences that successfully merged with their pair) were filtered for quality with Sickle^37^ by not allowing any window with *Q* score under 20. The resulting sequences were globally aligned to a 16S rRNA gene sequence reference dataset from genome assemblies of the synthetic community members. For strains that do not have an intact 16S rRNA sequence in their assembly, Sanger sequencing was used to obtain the 16S rRNA gene sequence of the strains for inclusion in the reference dataset. The reference dataset also included sequences from *Arabidopsis* organellar sequences and known bacterial contaminants. Sequence alignment was performed with USEARCH v.7.1090^38^ using the optional usearch_global at a 98% identity threshold. On average, 85% of read sequences matched an expected isolate. The 185 isolates of our 185-member synthetic community could not all be distinguished from one another on the basis of the V3-V4 sequence. They were thus classified into 97 unique sequences encompassing a set of identical (clustered at 100%) V3-V4 sequences coming from a single or multiple isolate strains. An isolate abundance table was created from the sequence mapping results.

We estimated 16S rRNA absolute abundance using a plasmid spike-in method^39^. Synthetic DNA was spiked at known quantities into samples before DNA extraction and the ratio of added to recovered synthetic DNA served as a conversion factor by which the total number of 16S rRNA molecules in a given sample was estimated. We designed a plasmid which included 16S V3-V4 primer binding sequences flanking a randomly generated DNA sequence matching the most frequent length and Guanine + Cytosine (GC) content of amplicons generated using the same primer sequences from wild soil. These sequences were synthesized by Geneart (Invitrogen) and supplied cloned in plasmid pMA-T. The plasmid was transformed into *E. coli* and isolated using a miniprep spin kit (Qiagen). Specific volumes of this isolated plasmid were then added to individual samples before DNA extraction to spike-in approximately 20% of the predicted 16S copies occurring within the sample. We performed colony-forming units (c.f.u.) counting using similarly treated plant samples (that is, growth on SynCom-inoculated agar plates) to obtain an estimate of the 16S copy number per mg fresh weight of plant roots. We plated serial dilutions of plant root samples ground in MgCl_2_ on LB to perform c.f.u. counts. The c.f.u. count multiplied by a given sample’s fresh weight were used to calculate sample-specific predicted 16S copy numbers.

### Plant growth conditions and root growth inhibition assay

*A. thaliana* ecotype Col-0 seeds were sterilized in 70% household bleach, 0.2% Tween-20 for 10 min with vigorous agitation and then rinsed 10 times with sterile distilled water. Seeds were stratified at 4 °C in sterile distilled water for 1–2 d. Plants were germinated for 7 d on 0.5x MS agar medium (2.22 g l^−1^ PhytoTech Labs M-404: Murashige & Skoog modified basal medium with Gamborg vitamins, 0.5 g l^−1^ MES hydrate, pH adjusted to 5.7, solidified with 1% (w/v) agar) supplemented with 0.5% (w/v) sucrose in vertical 12 ×12 cm square plates under long-day conditions (21 °C/18 °C, 16 h light/8 h dark, day/night cycle). Then 8 to 10 plants were aseptically transferred to 12 ×12 cm plates containing 0.5x MS agar medium without sucrose where the medium surface was spread with the bacterial inoculum. For assays with IAA addition, 100 nM IAA was added to the medium before pouring the plates. The plant root tip location was marked on plates after transfer to record the initial root tip position. The plates containing the plants and bacteria were incubated vertically under short-day conditions (22 °C/18 °C, 9 h light/15 h dark, day/night cycle) for an additional 11 d. Plates were imaged on a document scanner and primary root elongation was determined using imageJ to quantify the change in root tip position from the initial to the final position.

### Bacterial inoculation of plants

Individual bacterial strains were grown on agar plates of the media types specified above at 28 °C. Before plant inoculation, a single colony was picked into the appropriate liquid medium and grown at 28 °C to late exponential or early stationary phase. To remove the medium from the bacteria before inoculation, strains were washed three times in sterile 10 mM MgCl_2_. The optical density at 600 nm (OD_600_) was measured for each washed strain and normalized to OD_600_ of 0.01 in 10 mM MgCl_2_. For plant experiments with mono-association of an individual strain, 100 µl of OD_600_ = 0.01 washed bacteria was spread on the 12 ×12 cm plate before plant transfer. For experiments in duo-association with *Arthrobacter* CL28, 100 µl of OD_600_ = 0.01 washed *Arthrobacter* CL28 was spread along with 100 µl of OD_600_ = 0.01 of the second strain.

The 175-member synthetic community (SC185-10V) was prepared as described for the 185-member synthetic community used previously^14^ by leaving out the 10 isolates from the genus *Variovorax*. Briefly, 7 d before plant transfer, strains were inoculated individually into 600 µl KB medium in a 96-well plate and grown at 28 °C for 5 d. At 2 d before plant transfer, 20 ul from these 5-day-old cultures were transferred to 380 ul fresh KB medium in a new set of 96-well plates and both sets of plates were returned to the incubator for 2 d. This resulted in two cultures of each strain, one 7 d old and the other 2 d old, which were combined. The OD_600_ of the strains in each well was measured and the strains were combined while normalizing the OD_600_ of each strain in the pool. This pool was washed twice with 10 mM MgCl_2_ and diluted to OD_600_ = 0.2. For experiments with the SC185-10V SynCom, 100 µl of this OD_600_ = 0.2 washed pool was spread on 12 ×12 cm plates. For treatments where an additional strain was added to the SC185-10V SynCom, the individual strain was washed as described above, diluted to OD_600_ = 0.0034 in 10 mM MgCl_2_, and 100 µl of this dilution was spread on the plates with the SC185-10V SynCom. This addition of the individual strain corresponded to an OD_600_ three times that of a single strain in the SC185-10V SynCom (0.0034 = (0.2/175) × 3). For the addition of the 10 *Variovorax* strains to the SC185-10V SynCom experiment, the 10 *Variovorax* strains were grown individually in 2xYT medium from colonies grown on plates. The OD_600_ values of the 10 cultures were measured and the 10 strains were pooled while normalizing the OD_600_ of each strain to the same value. This mixture of the 10 *Variovorax* strains was then treated as the individual strains for washing and addition of 100 µl of OD_600_ = 0.0034 to the SC185-10V SynCom on plates.

### Construction of vectors with *Variovorax* CL14 *iad* gene inserts

Portions of the *V. paradoxus* CL14 IAA degradation locus were subcloned into broad host range vector pBBR1MCS-2^32^. Primers JMC579 through JMC604 (Supplementary Table 8) were used to amplify 3–5 kb segments of the locus by PCR using Q5 DNA polymerase (New England Biolabs). These primers were designed to amplify sections beginning and ending at gene start codons and with appropriate overlapping sequences for Gibson assembly either into the pBBR1MCS-2 backbone or to the adjacent section to make larger vector inserts, as appropriate. The pBBR1MCS-2 vector backbone was prepared for Gibson assembly by amplifying the vector by PCR using primers JMC577 and JMC578 (Supplementary Table 8) and subsequently treating with DpnI to remove circular vector template. PCR fragments were cleaned up as necessary using the QIAquick PCR purification kit (Qiagen). Appropriate fragments were mixed to construct the vectors by Gibson assembly using HiFi DNA Assembly Mastermix (New England Biolabs) according to manufacturer instructions. Gibson assembly products were transformed into NEB 10beta chemically competent *E. coli* (New England Biolabs) and selected on LB plates supplemented with 50 µg ml^−1^ kanamycin. Vectors were miniprepped using either the ZR plasmid miniprep classic kit or Zymo BAC DNA miniprep kit (Zymo Research) and confirmed via restriction mapping with PstI-HF (New England Biolabs) and Sanger sequencing (Genewiz).

To construct vectors that are derivatives of pBBR1::70–66, the Q5 site-directed mutagenesis kit (New England Biolabs) was used for gene deletion. Briefly, vector pBBR1::70–66 was used as a PCR template and portions of this vector were amplified by PCR using primers JMC641 through JMC650 (Supplementary Table 8) and Q5 DNA polymerase (New England Biolabs). PCR products were cleaned up and circularized using KLD Mastermix (New England Biolabs). The product was transformed into NEB 10beta chemically competent *E. coli* (New England Biolabs) and selected on LB plates supplemented with 50 µg ml^−1^ kanamycin. Vectors were miniprepped and Sanger sequenced as described above to confirm the construction of the correct vectors.

### Conjugation of vectors to *V. paradoxus* CL14 ΔHS33

Vectors were conjugated into *V. paradoxus* CL14 ΔHS33 using tri-parental mating. The helper *E. coli* strain carrying plasmid pRK2013^40^ and donor NEB 10beta *E. coli* strains containing the pBBR1MCS-2-based vectors with *Variovorax* IAA degradation locus gene inserts were cultured in LB media containing 50 µg ml^−1^ kanamycin at 37 °C. *V. paradoxus* CL14 ΔHS33 was grown in 2xYT medium containing 100 µg ml^−1^ ampicillin at 28 °C. *V. paradoxus* CL14 wild type and derivative strains such as ΔHS33 are naturally resistant to ampicillin and this ampicillin selection allows for recovery of only *Variovorax* from the conjugation reaction. To prepare for conjugation, all bacteria were pelleted by centrifugation at 5,000 × *g* for 5 min and washed 3 times in 2xYT medium without antibiotics. For each conjugation reaction, equal volumes (100–300 µl) of each of the three washed bacteria: recipient *V. paradoxus* CL14 ΔHS33, donor NEB 10beta *E. coli* containing a pBBR1MCS-2-based vector, and helper *E. coli* pRK2013 were mixed. Control conjugation mixtures of each pair of strains and individual strains alone were performed in parallel to ensure successful selection of exconjugants only from mixtures of all three strains together. Conjugation mixtures were pelleted by centrifugation at 5,000 × *g* for 5 min, resuspended in 50 µl 2xYT media, transferred to LB media plates without antibiotics and allowed to dry in a laminar flow hood. These conjugation plates were incubated overnight at 28 °C. After 18–24 h, exconjugants were selected by streaking from the pooled conjugation mixtures on the LB plate without antibiotics to LB plates containing 50 µg ml^−1^ kanamycin and 100 µg ml^−1^ ampicillin. This selects for only *V. paradoxus* CL14 ΔHS33 (ampicillin resistant) containing the pBBR1MCS-2-based vector (kanamycin resistant). Individual colonies were picked into and subsequently cultured in 2xYT medium containing 50 µg ml^−1^ kanamycin and 100 µg ml^−1^ ampicillin at 28 °C.

### Construction of *V. paradoxus* CL14 gene deletions

Unmarked gene deletions in *V. paradoxus* CL14 were constructed as described previously^14^ using the suicide vector backbone pMo130 originally developed for gene knockouts in *Burkholderia* spp.^41^. Primers JMC203 and JMC204 (Supplementary Table 8) were used to amplify the pMO130 vector backbone by PCR. This product was subsequently treated with DpnI (New England Biolabs) to digest circular template DNA. Primers JMC605 through JMC612 and JMC671 through JMC677 (Supplementary Table 8) were used to amplify flanking regions for the gene deletion targets from *V. paradoxus* CL14 genomic DNA. All PCR was performed using Q5 DNA polymerase (New England Biolabs) and products were cleaned up, as appropriate, with the QIAquick PCR purification kit (Qiagen). These PCR products were assembled into suicide vectors using HiFi Gibson Assembly Mastermix (New England Biolabs), transformed into chemically competent NEB 5alpha *E. coli* (New England Biolabs), and selected on LB plates with 50 µg ml^−1^ kanamycin. Vectors were miniprepped using the ZR plasmid miniprep classic kit (Zymo Research) and confirmed by Sanger sequencing (Genewiz). Confirmed vectors were transformed into the chemically competent bi-parental mating strain *E. coli* WM3064. Transformants were selected at 37 °C on LB media supplemented with 50 µg ml^−1^ kanamycin and 0.3 mM diaminopimelic acid (DAP), and single colonies picked into LB medium also with 50 µg ml^−1^ kanamycin and 0.3 mM DAP.

Bi-parental mating was performed by growing *E. coli* WM3064 containing the appropriate suicide vector as described above, and *V. paradoxus* CL14 was grown in 2xYT medium containing 100 µg ml^−1^ ampicillin at 28 °C. Both *E. coli* and *Variovorax* were washed separately three times using 2xYT medium, then mixed in a 1:1 ratio and pelleted. All centrifugation steps were performed at 5,000 × *g* for 5 min. The pelleted conjugation mixtures were resuspended in 1/10 the volume of 2xYT, plated on LB agar with 0.3 mM DAP and grown at 28 °C overnight. Exconjugants from these plates were streaked out and grown on LB agar with 100 µg ml^−1^ ampicillin, 50 µg ml^−1^ kanamycin, and no DAP at 28 °C. These strains were purified by streaking and growing on plates of the same medium once more. These strains with suicide vector integration were then grown once in liquid LB containing 100 µg ml^−1^ ampicillin and 1 mM isopropyl 1-thio-d-galactopyranoside (IPTG) at 28 °C and then streaked on plates containing media with 10 g l^−1^ tryptone, 5 g l^−1^ yeast extract, 100 g l^−1^ sucrose, 1.5% agar, 100 µg ml^−1^ ampicillin and 1 mM IPTG. Colonies from these plates were picked and grown in the same liquid media. These strains were then assessed for gene deletion by PCR using primers JMC657 through JMC660 and JMC697 through JMC699 (Supplementary Table 8). The Quick-DNA miniprep kit (Zymo Research) was used to isolate all genomic DNA for PCR screening. To purify the knockout strains, they were streaked and grown out three times on LB plates containing 100 µg ml^−1^ ampicillin before a final PCR confirmation. To check the purity of the final strains, PCR was performed with one primer outside the deletion region and one inside the deleted gene to ensure no product is produced for the knockout strain. The sequences for the primers used for this PCR reaction (JMC691, JMC717, JMC718, JMC693 and JMC694) can be found in Supplementary Table 8.

### Measurement of bacterial growth and IAA degradation

Individual strains were grown in 5 ml cultures in various media types supplemented with IAA at 28 °C and 250 r.p.m. To screen the *V. paradoxus* CL14 ΔHS33 pBBR1 vector complemented mutants, 2xYT medium supplemented with 0.1 mg ml^−1^ IAA was used. For comparison of other *V. paradoxus* CL14 mutants, M9 medium with 15 mM succinate and 0.1 mg ml^−1^ (0.57 mM) IAA was used. For comparison of IAA-degrading strains from diverse genera, M9 medium with 0.1% (w/v) casamino acids (Bacto) and 0.1 mg ml^−1^ IAA was used. For *R. pomeroyi*, 2% (w/v) sea salts were added to this M9 medium with casamino acids and IAA. The pBBR1 vector library in *E. coli* NEB 10beta was screened in LB medium supplemented with 0.04 mg ml^−1^ IAA and grown at 37 °C and 250 r.p.m. For all media types, IAA was solubilized in 100% ethanol at 20 mg ml^−1^ and diluted to 0.1 mg ml^−1^ in the media, resulting in 0.5% (v/v) ethanol in the media.

To measure growth, a 200 µl sample was taken from the growing cultures and OD_600_ was determined on an Infinite M200 Pro plate reader (Tecan). Subsequently, cells were pelleted by centrifugation at 4,200 × *g* for 15 min and 50 µl of supernatant was transferred to a new 96-well plate and frozen at −80 °C until further analysis. IAA degradation was determined by thawing the plates containing 50 µl aliquots of culture supernatant and combining this with 100 µl of Salkowski reagent (10 mM ferric chloride and 35% perchloric acid)^42^. This was performed alongside mixing 50 µl of IAA standards with 100 µl of Salkowski reagent in the same 96-well plate format. Colour development was allowed to proceed for 1 h and absorbance was read at 530 nm on the Infinite M200 Pro plate reader (Tecan). The absorbances measured were converted to IAA concentration on the basis of the absorbances measured for the IAA standards.

### Liquid Chromatography Dual Mass Spectroscopy (LC–MS/MS) metabolomics on *Variovorax* IAA degradation

*V. paradoxus* CL14 was grown in 5 ml cultures of M9 minimal medium supplemented with either 0.1 mg ml^−1^ IAA, 0.1 mg ml^−1^ ^13^C6-IAA (with the 6 carbons of the benzene ring of the indole labelled, Cambridge Isotope Laboratories CLM-1896-PK), and/or 15 mM succinate. Cultures and parallel media controls were incubated at 28 °C with shaking at 250 r.p.m. Cultures and media controls were centrifuged (4,200 × *g* for 15 min at 4 °C) to pellet cells; supernatants were transferred to new tubes and both pellets (intracellular fraction) and supernatants (extracellular fraction) were stored frozen at −80 °C until extraction. All subsequent work was performed over dry ice or in chilled cold blocks. Frozen pellets from the intracellular fraction were thawed for 3 h at 4 °C, then 800 µl of cold LCMS-grade water was added to the pellets with repeated pipetting to break up the pellet until visually homogeneous. Samples were then quickly returned to −80 °C to freeze the suspension. Frozen pellet suspensions and extracellular solutions were lyophilised until dry. The cells from the dried pellet suspensions were lysed and homogenized with a bead mill (BioSpec Mini-Beadbeater-96) using one sterile 3.2 mm steel ball in each tube for 3 rounds of 5 s each with 10 s breaks in between to reduce heat production. Dried extracellular samples were concentrated by resuspension in 100 µl LCMS- grade methanol, vortexed 3 times for 10 s each, water bath sonicated for 20 min, incubated at 4 °C overnight, centrifuged (1,000 × *g*, 4 °C, 5 min), and the methanol supernatant was dried using a speed vacuum concentrator. On the day of LC–MS/MS analysis, homogenized dry material was suspended in LCMS-grade methanol with internal standard mix (100 µM U-^13^C/^15^N-labelled amino acids, SIGMA 767964). Intracellular samples were suspended at 11.1 µl mg^−1^ of original sample cell pellet wet weight; extracellular samples were suspended at 38.9 µl mg^−1^ of corresponding cell pellet wet weight from the culture. The solutions were vortexed 3 times for 10 s each, bath sonicated in ice water for 10 min, chilled at −20 °C for 10 min, then centrifuged (10,000 × *g*, 5 min, 10 °C) to pellet insoluble material. Supernatants containing the methanol extracts were filtered through 0.22 µm PVDF microcentrifuge filtration tubes (10,000 × *g*, 5 min, 10 °C); filtrates were transferred to glass vials and immediately capped. Filtrates were then analysed by LC–MS/MS using an Agilent 1290 UHPLC system connected to a Thermo Q Exactive Hybrid Quadrupole-Orbitrap mass spectrometer equipped with a heated electrospray ionization (HESI-II) source probe. Extracts were chromatographically separated on a ZORBAX RRHD Eclipse Plus C18, 95 Å, 2.1 × 50 mm, 1.8 µm column (Agilent) for non-polar metabolomics. Separation, ionization, fragmentation and data acquisition parameters are specified in Supplementary Table 7. Briefly, metabolites were separated by gradient elution followed by MS1 and data-dependent (top 2 most abundant MS1 ions not previously fragmented in last 7 s) MS2 collection; targeted data analysis was performed by comparison of sample peaks to a library of analytical standards analysed under the same conditions or by searching the raw data files for predicted *m*/*z* values based on structural information of compounds of interest. Three parameters were compared: matching *m*/*z*, retention time and fragmentation spectra using Metabolite Atlas (https://github.com/biorack/metatlas)^43,44^. Identification and standard reference comparison details are provided in Supplementary Table 6. Raw and processed data are available for download at the JGI Joint Genome Portal under ID 1340427. Statistical comparisons were performed using R version 3.6.2, using package agricolae 1.3–5 and stats 3.6.2^45^; boxplots were generated with base R graphics using the boxplot function.

### Phylogenomic analysis

To guide the delineation of the IAA degradation operons across the bacterial tree of life, we constructed two Hidden Markov Model (HMM) profiles of the genes *iacC* and *iacD* by subsetting all homologous genes from the previously validated operons (Extended Data Fig. 4). In parallel, we downloaded the assembly files for all available complete genomes deposited in the NCBI RefSeq 202 repository^46^. For the 220,000 assembly files downloaded, we performed open reading frame (ORF) prediction using prodigal. We then used the two HMM profiles described above to query the predicted ORFs. Utilizing ad hoc scripts, we constructed a table of HMM hits along the genomes scanned and subset genomic loci where both *iacC* and *iacD* genes appeared adjacent to one another. The logic of using the *iacC* and *iacD* genes as anchor genes for our search is that the adjacent physical location of both *iacC* and *iacD* homologues is a conserved feature across all previously experimentally validated IAA-degrading operons (Extended Data Fig. 4). Next, for each region containing the adjacent *iacC* and *iacD* homologue genes, we extracted the gene neighbourhood adjacent to the anchor hit by extracting the amino acid sequence of ORFs +10 kb and −10kb with respect to the anchor hit. Using hmmscan from the Hmmer v3.1.b2 suite^47^, we performed HMM profiling in all ORFs extracted via our neighbourhood delineation against the COG database version 2003. Finally, we used the COG profiles across the neighbourhoods to create a matrix describing the prevalence of COGs across the regions (candidate regions) with the adjacent *iacC* and *iacD* homologue genes.

For each genome containing at least one candidate region, we performed taxonomic classification using the GTDB database^48^. Due to the size of our estimated genomic matrix and to reduce potential biases due to over-representation of certain lineages within RefSeq, we performed principal coordinate analysis (PCoA) using a reduced matrix containing one representative candidate region per species. Species labelling was obtained from the GTDB taxonomic classification described above. PCoA was performed using the oh.pco function from the ohchibi package^49^, taking as input a binary version of the gene matrix described above. We classified candidate reads into the two types of IAA-degrading operon (*iac*-like and *iad*-like), utilizing a majority count-based approach using marker COGs conserved between the previously experimentally validated IAA-degrading operons (Extended Data Fig. 4). Specifically, for each potential operon, we determined the prevalence of COGs that a priori (Extended Data Fig. 4) showed differential prevalence across the two degrading operons (for example, *iacA*, *iacB* and *iacI* are markers of the *iac* operon, while *iorB/iadB* and *iotA/iadA* are exclusive markers of the *iad*-like operon). Hybrid gene clusters were defined as operons that exhibited the hallmark COGs of both operons.

In parallel, we performed phylogenetic inference over all the genomes belonging to genera with at least one representative strain harbouring any of the two types of IAA-degrading operon. This phylogenetic tree was constructed using a super-matrix-based approach as previously described^35^. Finally, for each genus with at least one assembly harbouring a positive IAA-degrading operon, we estimated the prevalence of the trait across the genus by dividing the total number of isolates with detectable IAA degradation locus by the total number of isolates belonging to that genus in the dataset. In addition, to see the phylogenetic evenness of the distribution of the IAA degradation trait across each genus, we calculated the phylogenetic ratio by calculating the ratio between the average phylogenetic distance (computed via the cophenetic.phylo function from the ape R package^50^) of isolates with a detectable IAA degradation locus and the total average phylogenetic distance of all isolates within that genus. We constructed the MarR phylogeny using the MarR sequences from candidate regions with 100% markers of one of the two types of IAA-degrading operon. Amino acid sequences of the MarR homologues were aligned using MAFFT^51^ and phylogenetic inference was performed using FastTree 2^52^.

### RNA-seq on *Variovorax* strains

*V. paradoxus* CL14 was grown in 5 ml cultures of M9 minimal medium supplemented with 15 mM succinate and 0.5% (v/v) ethanol alone or containing IAA. IAA was at a final concentration of 0.1 mg ml^−1^ in the medium to which it was added. Cultures were prepared at a starting OD_600_ of 0.02 and incubated at 28 °C, shaking at 250 r.p.m. Cells from all samples were collected for RNA-seq at 18 h to ensure IAA was still present in the cultures of strains that degraded IAA most rapidly. Cells were pelleted by centrifuging the culture at 4,200 × *g* for 15 min and removing the supernatant. Cell pellets were frozen at −80 °C before RNA extraction. To extract RNA, cells were lysed in TRIzol reagent (Invitrogen) according to manufacturer instructions for lysis and phase separation. After these steps, RNA was purified from the aqueous phase using the RNeasy mini kit (Qiagen) including the optional on-column DNase digestion with RNase-free DNase set (Qiagen). Total RNA was quantified using the Qubit 2.0 fluorometer (Invitrogen) and RNA-seq libraries were prepared using the Universal Prokaryotic RNA-Seq Prokaryotic AnyDeplete kit (Tecan) according to manufacturer instructions. The resulting libraries were pooled and sequenced on the Illumina HiSeq4000 to generate 50 bp single-end reads.

### RNA-seq data analysis

The *V. paradoxus* CL14 RNA-seq sequence data were analysed as described previously^14^. Briefly, the raw reads were mapped to the *V. paradoxus* CL14 genome (fasta file available at https://github.com/isaisg/variovoraxRGI/blob/master/rawdata/2643221508.fna) using bowtie2^53^ with the ‘very sensitive’ flag. Hits to each individual coding sequence were counted and annotated using the function featureCounts from the R package Rsubread^54^, inputting the *V. paradoxus* CL14 gff file (available at https://github.com/isaisg/variovoraxRGI/blob/master/rawdata/2643221508.gff) and using the default parameters with the flag allowMultiOverlap = FALSE. Finally, DESeq2^55^ was used to estimate Differentially Expressed Genes (DEGs) between treatments, with the corresponding fold-change estimates and False Discovery Rate (FDR) adjusted *P* values. For visualization purposes, we performed *z*-score standardization of each gene across samples and we visualized this standardized expression values utilizing a heat map constructed using ggplot2^56^. These data can be found in Supplementary Table 9.

### MarR protein expression and purification

The coding sequence for each gene can be found in Supplementary Table 10. MarR expression plasmids were synthesized as codon-optimized genes for *E. coli* expression by BioBasic in the pLIC-His N-term vector (pMCSG7) and transformed into *E. coli* BL21 (DE3) Gold cells for expression. Cells were grown in the presence of ampicillin in LB medium with shaking at 225 r.p.m. at 37 °C to an OD_600_ of 0.5, at which point the temperature was reduced to 18 °C. At an OD_600_ of 0.8, protein expression was induced by the addition of 0.1 mM IPTG and incubation continued overnight. Cells were collected by centrifugation at 4,500 × *g* for 20 min at 4 °C in a Sorvall (model RC-3B) swinging bucket centrifuge. Cell pellets were resuspended in buffer A (20 mM potassium phosphate, pH 7.4, 50 mM imidazole, 500 mM NaCl), DNase, lysozyme and a Roche Complete EDTA-free protease inhibitor tablet. Resuspended cells were sonicated and clarified via centrifugation at 17,000 × *g* for 60 min in a Sorvall (model RC-5B) swinging bucket centrifuge. The lysate was applied to a nickel-nitrilotriacetic acid HP column (GE Healthcare) on an Aktaxpress Fast Performance Liquid Chromatography (FPLC) system (Amersham Bioscience) and washed with buffer A. Protein was eluted with buffer B (20 mM potassium phosphate, pH 7.4, 500 mM imidazole, 500 mM NaCl). Fractions containing the protein of interest were combined and passed over a HiLoadTM 16/60 SuperdexTM 200 gel filtration column. Proteins were eluted in S200 buffer (20 mM HEPES, pH 7.4, 300 mM NaCl). Fractions were combined and concentrated for long-term storage at −80 °C.

MarR mutant proteins were created by site-directed mutagenesis using primers from Integrated DNA Technologies. The mutant plasmids were sequenced to confirm the mutations. The mutants were produced and purified using *E. coli* BL21 (DE3) Gold as described above.

### Ligand binding studies by isothermal titration calorimetry (ITC)

All ITC measurements were performed at 25 °C using an Auto-ITC200 microcalorimeter (MicroCal/GE Healthcare). The buffer employed was 20 mM HEPES, pH 7.4, 50 mM NaCl and 0.5% dimethly sulfoxide (DMSO) for protein/ligand binding and 20 mM HEPES, pH 7.4 and 300 mM NaCl for DNA/protein binding experiments. For ligand binding experiments, the calorimetry cell (volume 200 ml) was loaded with MarR wild-type, mutant or homologue protein at a concentration of 50 μM. The syringe was loaded with a ligand concentration of 0.5 or 2 mM. For DNA binding experiments, wild-type MarR_73 did not bind any of the DNA oligos examined; however, we hypothesized that this arose from the ability of this native receptor to remain bound to ligands retained from its recombinant expression in *E. coli*. Thus, we employed the MarR_73 S28A protein with reduced ligand binding capacity. Here, the calorimetry cell was loaded with duplex oligo at a concentration of 25 μM and the syringe was loaded with MarR S28A mutant protein, which was necessary to prevent ligand binding during expression and purification, at a concentration of 0.5 mM. A typical injection protocol included a single 0.2 μl first injection followed by 20 1.5 μl injections of the syringe sample into the calorimetry cell. The spacing between injections was kept at 180 s and the reference power at 8 μcal s^−1^. The data were analysed using Origin for ITC version 7.0 software supplied by the manufacturer and fit well to a one-site binding model. Two independent ITC measurements were performed for each condition. A non-integer *N* value (for example, 0.73 in Fig. 2a) indicates that some protein monomers may not be in an active conformation, and thus do not bind ligand. Additionally, small measurement errors in assessing the protein or ligand concentrations may also contribute to non-integer N values in ITC. To confirm that 300 mM NaCl did not negatively impact DNA binding, MarR_73 S28A was examined by ITC in 150 mM NaCl. In this condition, the *K*_D_ for the 22 bp duplex was 0.428 ± 0.002 μM (*N* = 1.75 ± 0.014), while the *K*_D_ for 24 bp duplex was 0.151 ± 0.025 μM (*N* = 2.51 ± 0.26).

### Protein crystallography

*V. paradoxus* MarR_73 was crystallized using the sitting drop vapour diffusion method at 20 °C in conditions outlined in Supplementary Table 4. Crystallization drops were set up using the Oryx4 protein crystallization robot (Douglas Instruments) and contained 0.15 μl protein and 0.15 μl well solution. For all *V. paradoxus* MarR_73 wild-type conditions, ligands were added at 10-fold molar excess before crystallization trials and crystals appeared within 2–5 d. *V. paradoxus* MarR_73 with the S28A and R46A mutations was crystallized in similar conditions as the wild-type protein. Similarly, *P. putida* MarR_iacR, *B. japonicum* MarR_Bj1, *A. baumannii* MarR_Ab and *E. soli* MarR_Es were crystallized using vapour diffusion methods in sitting drop trays at 20 °C and crystals appeared within 3–5 d. All crystallization conditions are outlined in Supplementary Table 4. Crystal specimens were cryoprotected with the well solution supplemented with glycerol to 20% (v/v) (Supplementary Table 4). X-ray diffraction data were collected at the Advanced Photon Source beamline 23-ID-D (Supplementary Table 3). Diffraction images were reduced using either XDS or Denzo and scaled with either Aimless or Scalepack^57–59^. The *V. paradoxus* MarR_73 structure in complex with IAA was determined by molecular replacement using the structure of 3CDH as a search model in Phaser^60^. All subsequent structures of *V. paradoxus* MarR_73 were determined using the *V. paradoxus* MarR_73 IAA complex structure (PDB: 7KFO) as a search model. The *P. putida* MarR_iacR and *B. japonicum* MarR_Bj1 structures were determined by molecular replacement using the structure of 3CJN as the search model. *P. putida* MarR_iacR (PDB: 7KUA) was subsequently used as the search model for molecular replacement to solve *A. baumannii* MarR_Ab and *E. soli* MarR_Es. A nickel ion was placed in the model of MarR_Ab. The following ions or molecules were examined and refined in this location in the MarR_Ab structure: water, Na, Mg, K, Ca, Mn, Fe, Co, Ni, Cu, Zn and Ba. Water, Na, Mg, K, Ca and Ba were deemed unacceptable in this site due to poor difference density. Of the remaining ions considered, there were no sources of Mn, Fe, Co, Cu or Zn in the protein expression media, protein purification buffers, protein storage buffer, crystallization condition or cryoprotectant solutions. Thus, we concluded that the ion present in this structure is Ni due to the use of a nickel-affinity column during the protein’s purification. It is unclear why this ion remained bound to MarR_Ab even after the subsequent size exclusion chromatography purification step, or why such an ion is only observed in this structure of the proteins examined. All structures were refined with either Phenix.refine or Refmac using iterative model building in Coot to the final parameters outlined in Supplementary Table 3^61,62^. MarR_73 is a dimer with one protein monomer in the asymmetric unit and the dimer generated by crystallographic symmetry. PDB accession codes and associated crystallographic data are reported in Supplementary Table 3.

### Statistics and reproducibility

No statistical method was used to predetermine sample size, but our sample sizes are similar to those reported in previous publications^14,63,64^. No data were excluded from the analyses. The experiments were randomized. The investigators were not blinded to allocation during experiments and outcome assessment. Where not stated, data distribution was assumed to be normal, but this was not formally tested.

### Reporting summary

Further information on research design is available in the Nature Research Reporting Summary linked to this article.

### Supplementary information

Reporting Summary

Supplementary Table See tabs in Excel.

Supplementary Data 1 ITC data files.

Supplementary Data 2 ITC data files.

Supplementary Data 3 ITC data files.

Supplementary Data 5a Tree for Fig. 5a.

### Source data

Source Data Fig. 1 Source data.

Source Data Fig. 2 Source data.

Source Data Fig. 3 Source data.

Source Data Fig. 5 Source data.

Source Data Fig. 6 Source data.

Source Data Extended Data Fig. 1 Source data.

Source Data Extended Data Fig. 4 Source data.

Source Data Extended Data Fig. 5 Source data.

## Figures and Tables

**Extended Data Table 1:**
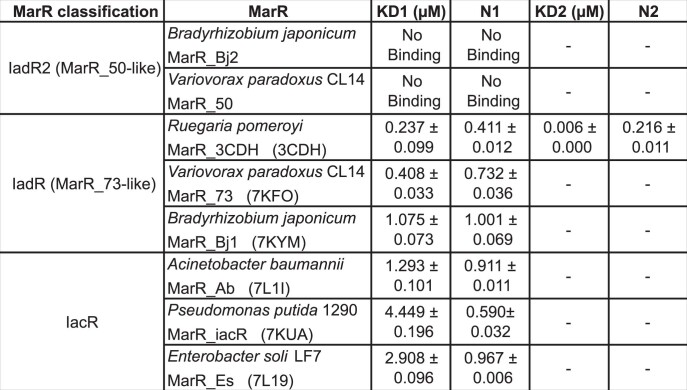
IAA binding of diverse MarR representatives from species previously shown to degrade IAA The K_D_ and N values for ligand binding are the mean from two independent ITC measurements ± standard error. MarRs are classified as defined in Fig. 5. PDB codes for structures are shown in parentheses

## Data Availability

The 16S rRNA amplicon sequencing data in this study have been deposited in the NCBI Sequence Read Archive under BioProject ID PRJNA768850. RNA-seq data associated with this study have been deposited in the NCBI Gene Expression Omnibus (GEO) database under accession GSE210968. Raw and processed metabolomics data are available for download at the JGI Joint Genome Portal https://genome.jgi.doe.gov/portal/ under ID 1340427. PDB (https://www.rcsb.org/) accession codes associated with this work are: 7KFO, 7KFQ, 7KIG, 7KKC, 7KKI, 7KH3, 7KJL, 7KJQ, 7KFS, 7KK0, 7KRH, 7KUA, 7KYM, 7L1I and 7L19. The associated crystallographic data for these structures are reported in Supplementary Table 3. Source data are provided with this paper.

## Code availability

All scripts required to reproduce the results of this study are available in the GitHub repository at https://github.com/isaisg/variovoraxRGImarR.
