# Supplementary material for: Diverse MarR bacterial regulators of auxin catabolism in the plant microbiome
Source: Nat Microbiol. 2022 Oct 20;7(11):1817–33. doi: 10.1038/s41564-022-01244-3 (PMC9613470; doi:10.1038/s41564-022-01244-3)
Supplement: Supplementary file 1 — Reporting Summary [file 41564_2022_1244_MOESM1_ESM.pdf]

nature portfolio

Corresponding author(s): Jeff Dangl & Matthew Redinbo

Last updated by author(s): Aug 13, 2022

Reporting Summary

Nature Portfolio wishes to improve the reproducibility of the work that we publish. This form provides structure for consistency and transparency in reporting. For further information on Nature Portfolio policies, see our Editorial Policies and the Editorial Policy Checklist.

Statistics

For all statistical analyses, confirm that the following items are present in the figure legend, table legend, main text, or Methods section.

n/a	Confirmed	
☐	☒	The exact sample size (n) for each experimental group/condition, given as a discrete number and unit of measurement
☐	☒	A statement on whether measurements were taken from distinct samples or whether the same sample was measured repeatedly
☐	☒	The statistical test(s) used AND whether they are one- or two-sided<br>*Only common tests should be described solely by name; describe more complex techniques in the Methods section.*
☐	☒	A description of all covariates tested
☐	☒	A description of any assumptions or corrections, such as tests of normality and adjustment for multiple comparisons
☐	☒	A full description of the statistical parameters including central tendency (e.g. means) or other basic estimates (e.g. regression coefficient) AND variation (e.g. standard deviation) or associated estimates of uncertainty (e.g. confidence intervals)
☐	☒	For null hypothesis testing, the test statistic (e.g. F, t, r) with confidence intervals, effect sizes, degrees of freedom and P value noted<br>*Give P values as exact values whenever suitable.*
☒	☐	For Bayesian analysis, information on the choice of priors and Markov chain Monte Carlo settings
☒	☐	For hierarchical and complex designs, identification of the appropriate level for tests and full reporting of outcomes
☒	☐	Estimates of effect sizes (e.g. Cohen's d, Pearson's r), indicating how they were calculated

Our web collection on statistics for biologists contains articles on many of the points above.

Software and code

Policy information about availability of computer code

Data collection	ImageJ v.1.52p was used for plant morphometric measurements. Bioinformatic analysis used: MT-Toolbox v4.1.2, Sickle v1.33, USEARCH v7.1090, hmmer v3.1b2, MAFFT v7.407, FastTree 2 v2.1.10, and bowtie2 v2.4.1.
Data analysis	Statistical analysis used: R v3.6.2 with packages: ohchibi v0.0.0.9, DESeq2 v1.24.0, Rsubread v2.2.2, ggplot2 v3.3.3<br>All scripts required to reproduce the results of this study are in the following GitHub repository: https://github.com/isaisg/variovoraxRGImarR

For manuscripts utilizing custom algorithms or software that are central to the research but not yet described in published literature, software must be made available to editors and reviewers. We strongly encourage code deposition in a community repository (e.g. GitHub). See the Nature Portfolio guidelines for submitting code & software for further information.

Data

Policy information about availability of data

All manuscripts must include a data availability statement. This statement should provide the following information, where applicable:

- Accession codes, unique identifiers, or web links for publicly available datasets
- A description of any restrictions on data availability
- For clinical datasets or third party data, please ensure that the statement adheres to our policy

The 16S rRNA amplicon sequencing with this study have been deposited in the NCBI Sequence Read Archive under BioProject ID PRJNA768850. RNASeq data associated with this study have been deposited in the NCBI Gene Expression Omnibus (GEO) database under accession GSE210968, respectively. Raw and processed metabolomics data are available for download at the JGI Joint Genome Portal https://genome.jgi.doe.gov/portal/ under ID 1340427. PDB accession codes associated with this work are: 7KFO, 7KFQ, 7KIG, 7KKC, 7KKI, 7KH3, 7KJL, 7KJQ, 7KFS, 7KKO, 7KRH, 7KUA, 7KYM, 7L1I, and 7L19. The associated crystallographic data for these structures are reported in Supplementary Table 3.

Field-specific reporting

Please select the one below that is the best fit for your research. If you are not sure, read the appropriate sections before making your selection.

- [x] Life sciences
- [ ] Behavioural & social sciences
- [ ] Ecological, evolutionary & environmental sciences

For a reference copy of the document with all sections, see nature.com/documents/nr-reporting-summary-flat.pdf

Life sciences study design

All studies must disclose on these points even when the disclosure is negative.

Sample size	Samples sizes are reported in the figure legends. No sample size calculation was performed. Sample sizes are consistent with previous manuscripts (Finkel et al 2020, Castrillo et al 2017). The size of experiments was established based on the maximal amount of replication that could be reasonably obtained considering physical space and labor constraints.
Data exclusions	A small number of plant samples were excluded from analysis when individual seedling growth was extremely stunted and rosette leaves did not develop beyond the cotyledons. This criteria is consistent with previous studies.<br>For 16S rRNA and RNASeq sequencing data quality control criteria are outlined in the Method section.
Replication	The reported sample sizes in the figure legends represent biologically independent samples. In planta experiments were replicated over two or three independent experiments as specified in the figure legends. Results from these independent experiments were similar and aggregated for data analysis. This study also builds on our previous study (Finkel et al. 2020), and controls performed as part of this study are consistent with the results of the previous study.
Randomization	All experiments were randomized. Plates containing plants were randomly ordered in racks and these racks were placed randomly in the growth chamber. Cultures were randomized in incubators.
Blinding	Where not stated the investigators were not blinded to allocation during experiments and outcome assessment. Root elongation was quantified in large batches without observing the experimental conditions on individual plates, and then aggregated based on condition for analysis. Bacterial growth and indole-3-acetic acid degradation data collection was blinded to the individual bacteria in the cultures by assigning numbers and positions to each independent culture and proceeding with data collection without knowing the bacteria contained in an individual sample. OD600 and IAA measurements were analyzed and then matched to a specific bacterial strain or species based on tube number and position.

Reporting for specific materials, systems and methods

We require information from authors about some types of materials, experimental systems and methods used in many studies. Here, indicate whether each material, system or method listed is relevant to your study. If you are not sure if a list item applies to your research, read the appropriate section before selecting a response.

Materials & experimental systems

n/a	Involved in the study
[x]	[ ] Antibodies
[x]	[ ] Eukaryotic cell lines
[x]	[ ] Palaeontology and archaeology
[x]	[ ] Animals and other organisms
[x]	[ ] Human research participants
[x]	[ ] Clinical data
[x]	[ ] Dual use research of concern

Methods

n/a	Involved in the study
[x]	[ ] ChIP-seq
[x]	[ ] Flow cytometry
[x]	[ ] MRI-based neuroimaging
