# Supplementary figures and images for: Diverse MarR bacterial regulators of auxin catabolism in the plant microbiome
Source: Nat Microbiol. 2022 Oct 20;7(11):1817–33. doi: 10.1038/s41564-022-01244-3 (PMC9613470; doi:10.1038/s41564-022-01244-3)

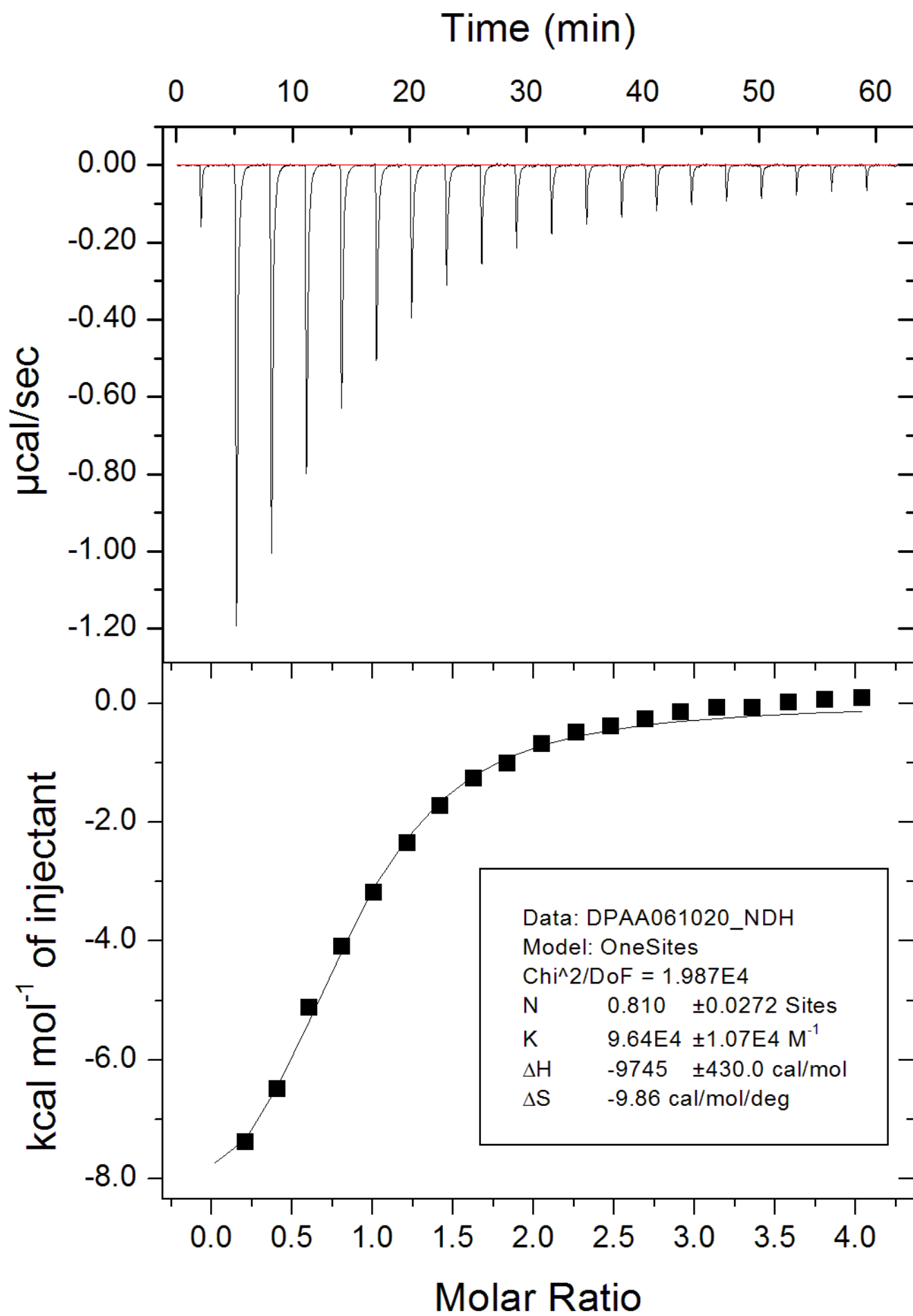

Supplement: Supplementary file 3 — ITC data files. [file 41564_2022_1244_MOESM3_ESM.zip › Variovorax_paradoxus_MarR_73_ligands_SUBMIT/Phenylacetic Acid/Variovorax_paradoxus_MarR_73_Phenylacetic Acid_itc1.pdf]

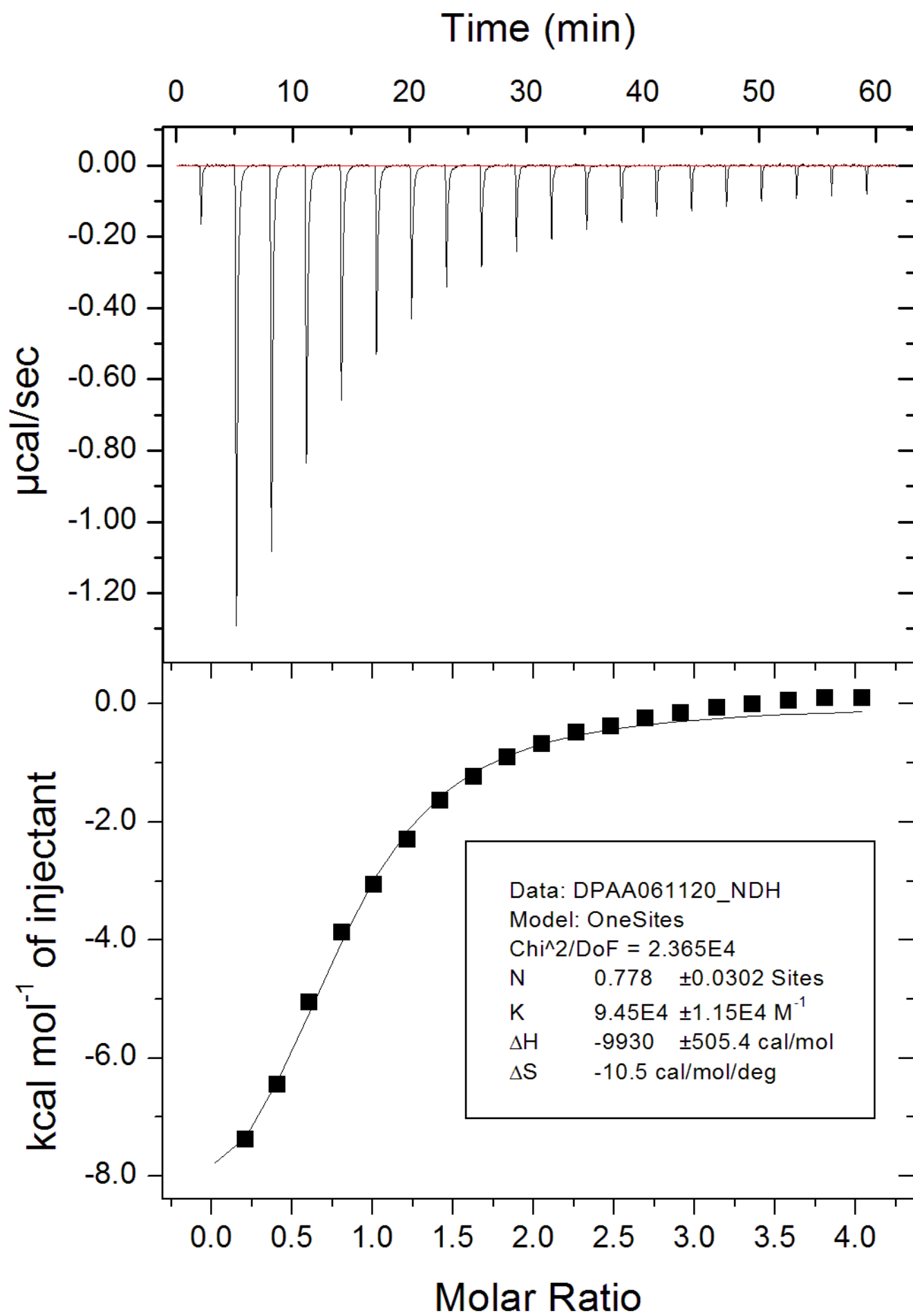

Supplement: Supplementary file 3 — ITC data files. [file 41564_2022_1244_MOESM3_ESM.zip › Variovorax_paradoxus_MarR_73_ligands_SUBMIT/Phenylacetic Acid/Variovorax_paradoxus_MarR_73_Phenylacetic Acid_itc2.pdf]

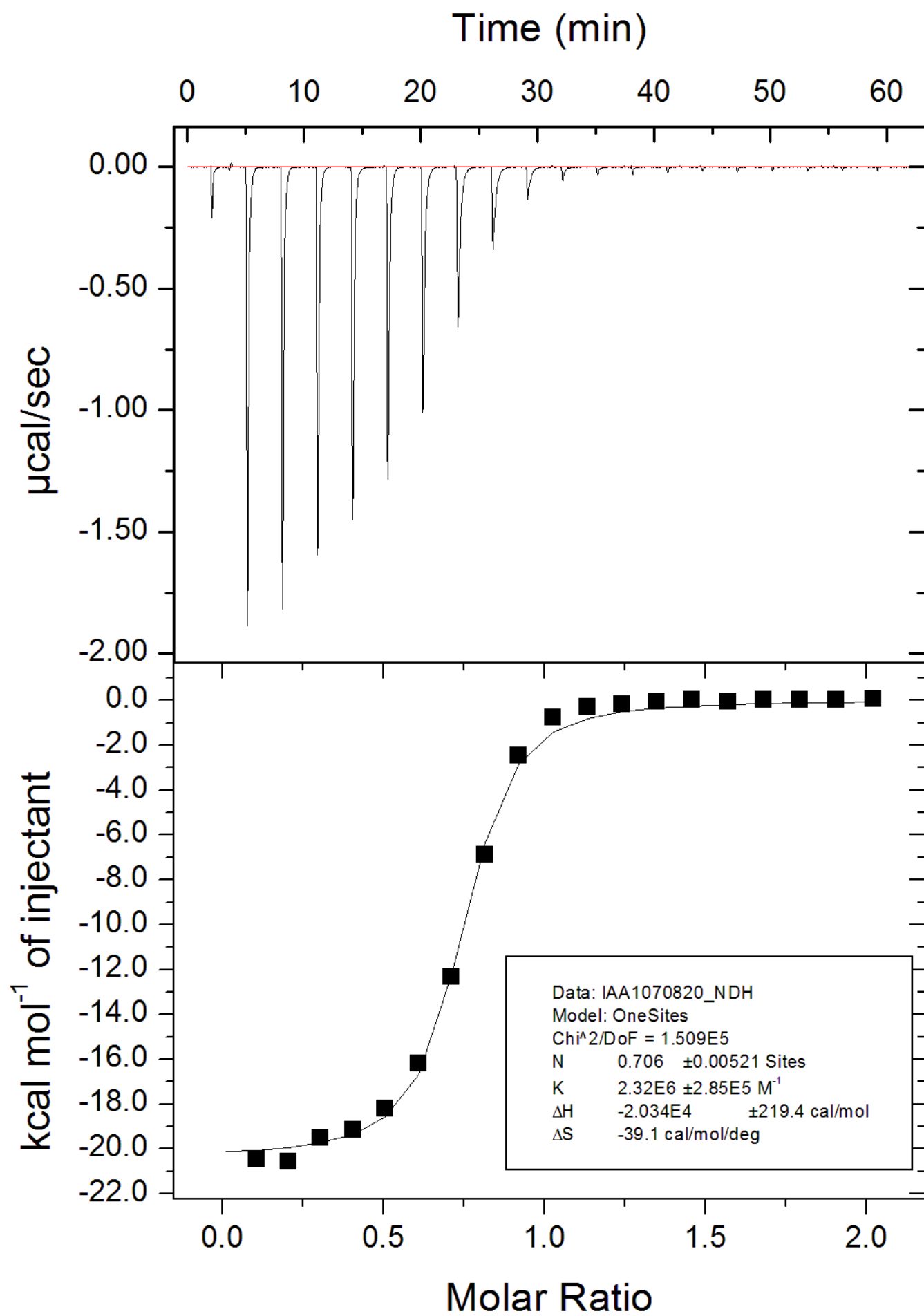

Supplement: Supplementary file 3 — ITC data files. [file 41564_2022_1244_MOESM3_ESM.zip › Variovorax_paradoxus_MarR_73_ligands_SUBMIT/Indole-3-Acetic Acid (IAA)/Variovorax_paradoxus_MarR_73_Indole-3-Acetic Acid (IAA)_itc1.pdf]

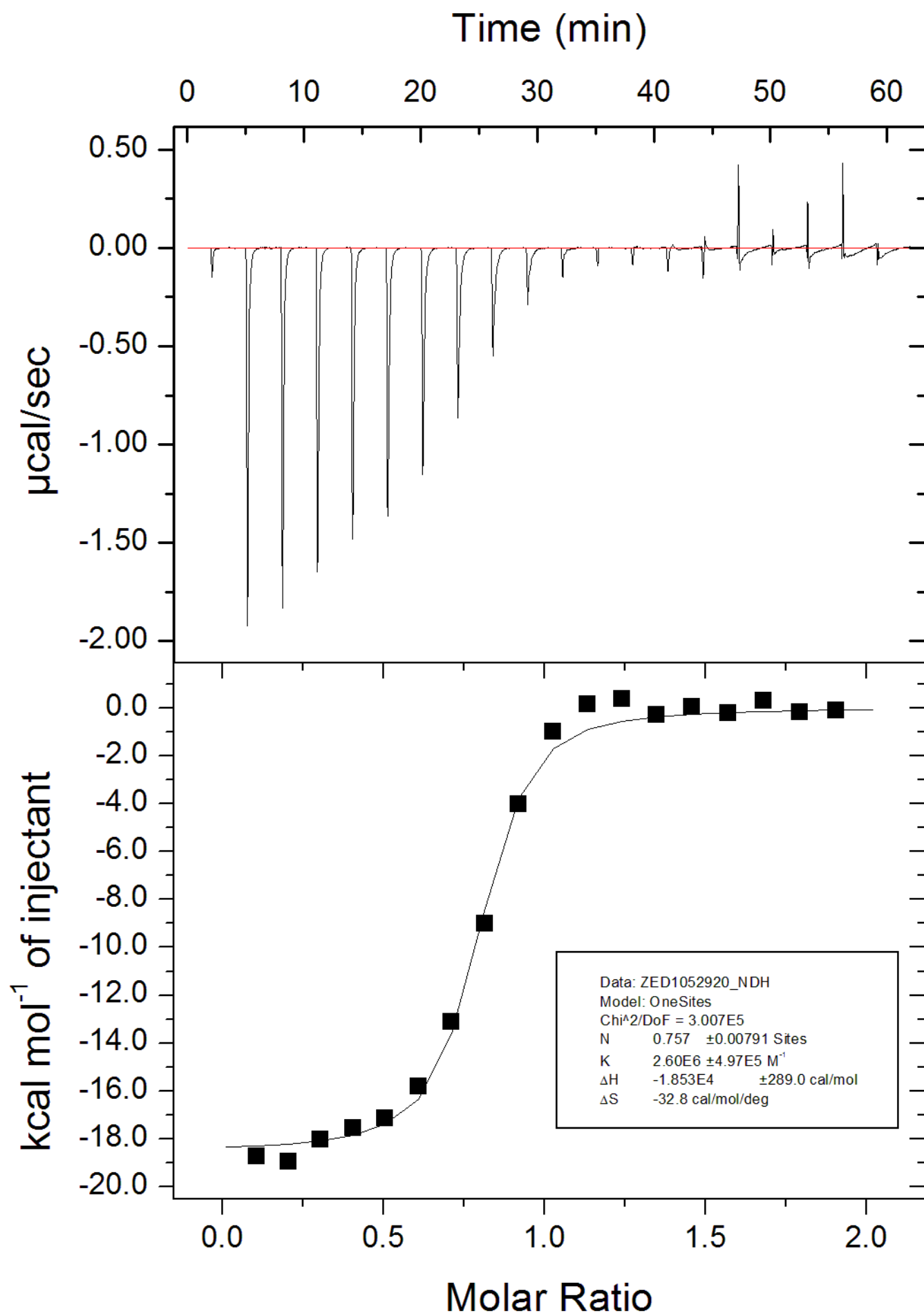

Supplement: Supplementary file 3 — ITC data files. [file 41564_2022_1244_MOESM3_ESM.zip › Variovorax_paradoxus_MarR_73_ligands_SUBMIT/Indole-3-Acetic Acid (IAA)/Variovorax_paradoxus_MarR_73_Indole-3-Acetic Acid (IAA)_itc2.pdf]

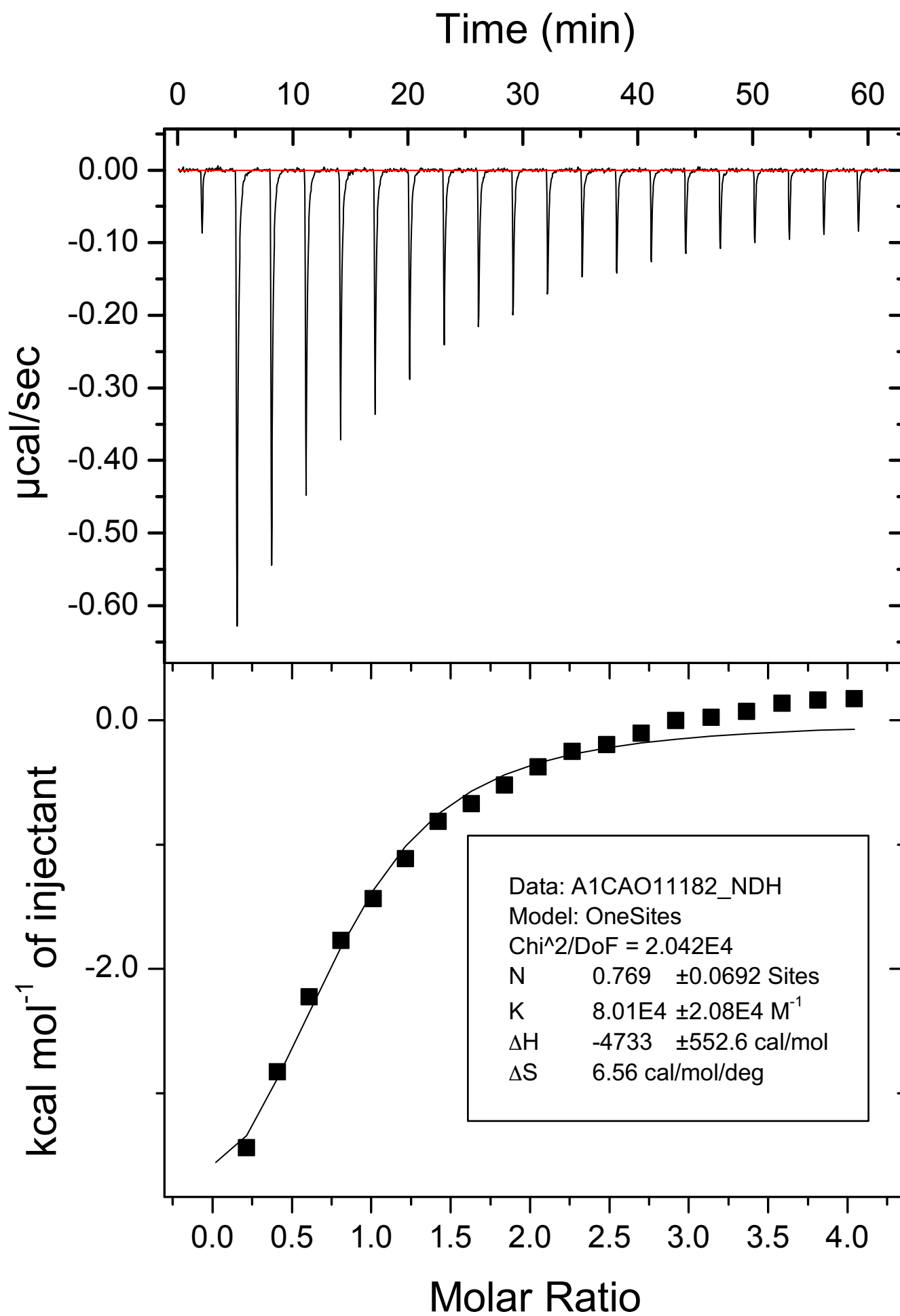

Supplement: Supplementary file 3 — ITC data files. [file 41564_2022_1244_MOESM3_ESM.zip › Variovorax_paradoxus_MarR_73_ligands_SUBMIT/Catachol (1,2-Dihydroxybenzene)/Variovorax_paradoxus_MarR_73_Catachol (1,2-Dihydroxybenzene)_itc1.pdf]

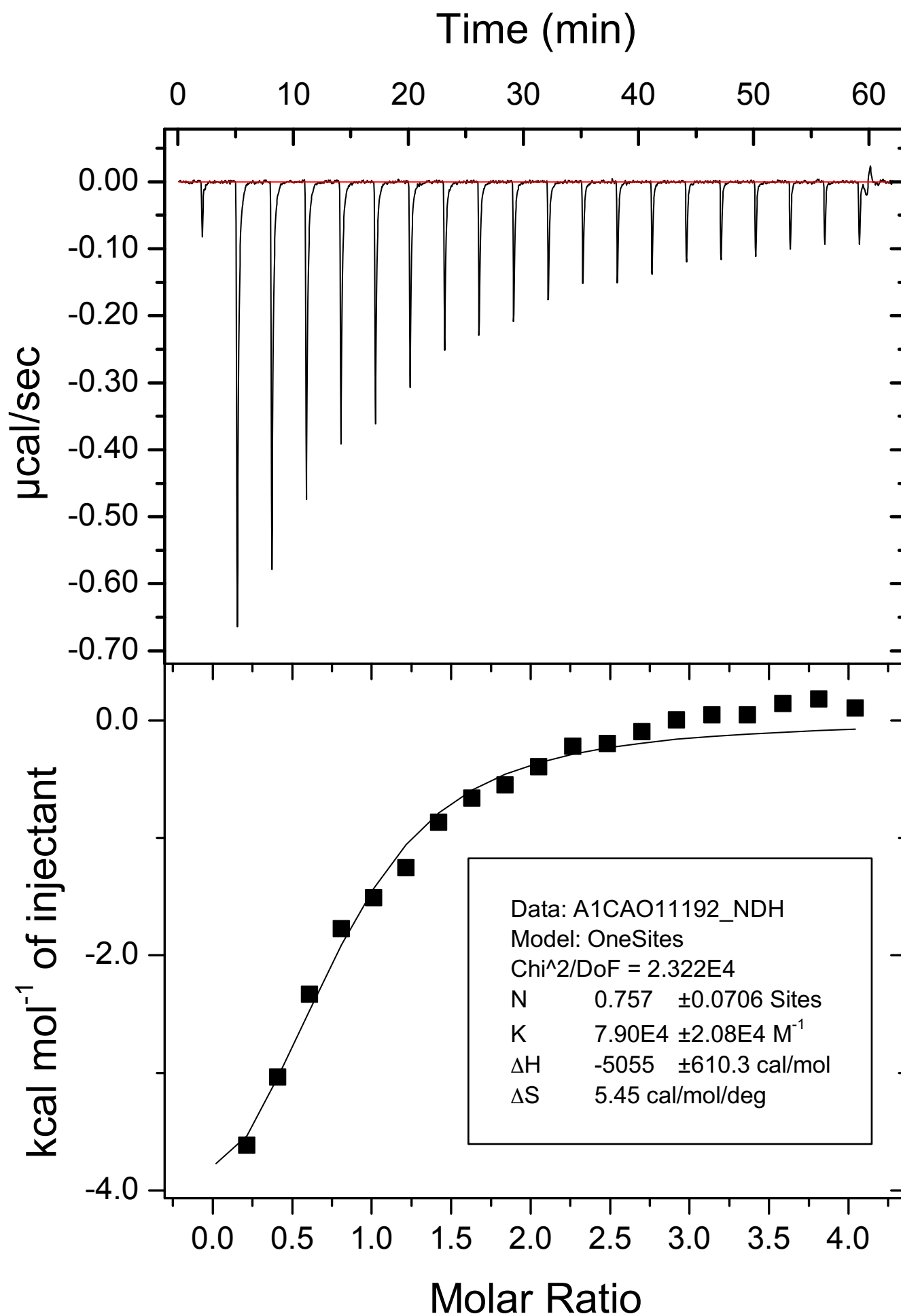

Supplement: Supplementary file 3 — ITC data files. [file 41564_2022_1244_MOESM3_ESM.zip › Variovorax_paradoxus_MarR_73_ligands_SUBMIT/Catachol (1,2-Dihydroxybenzene)/Variovorax_paradoxus_MarR_73_Catachol (1,2-Dihydroxybenzene)_itc2.pdf]

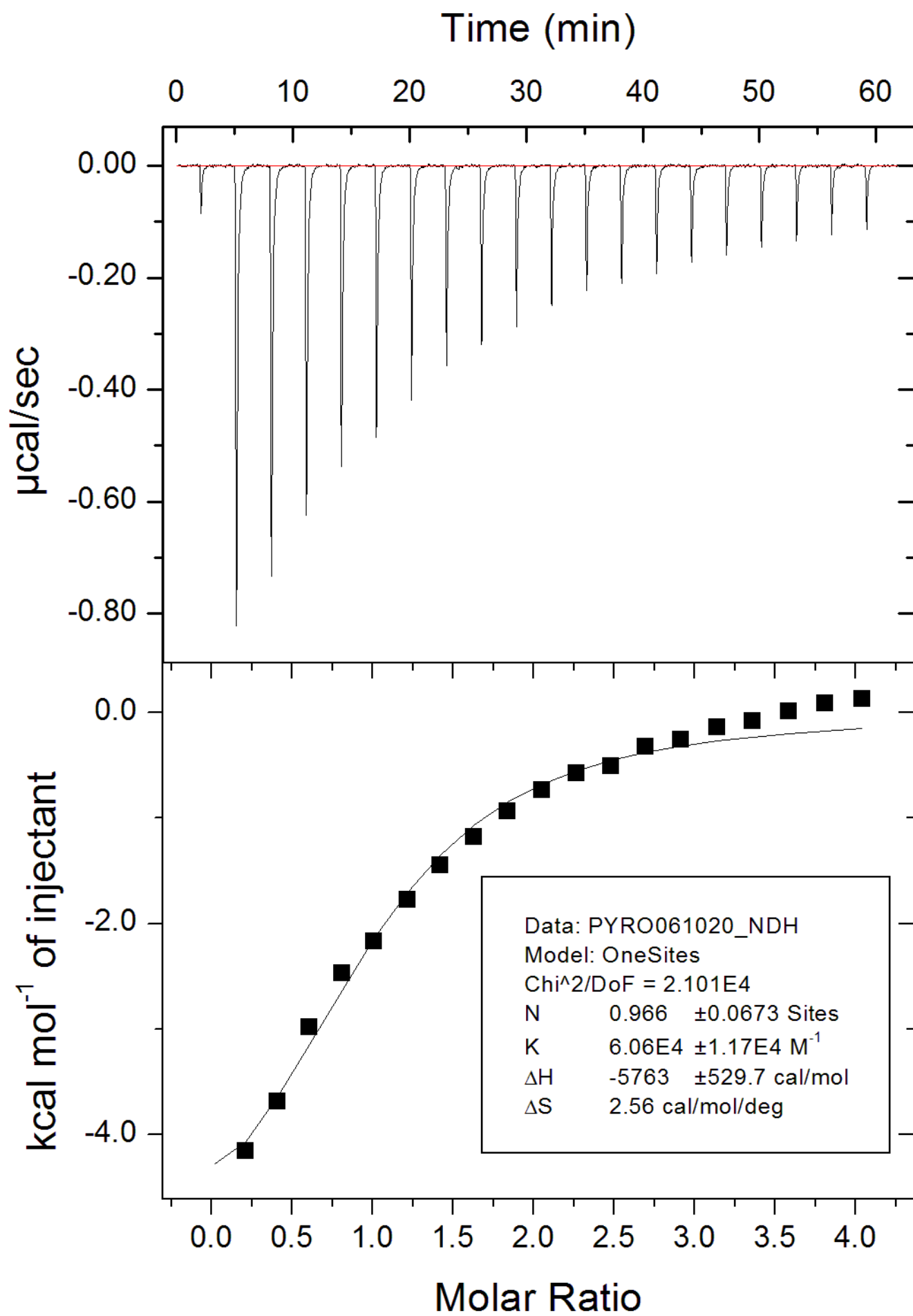

Supplement: Supplementary file 3 — ITC data files. [file 41564_2022_1244_MOESM3_ESM.zip › Variovorax_paradoxus_MarR_73_ligands_SUBMIT/Picloram /Variovorax_paradoxus_MarR_73_Picloram_itc1.pdf]

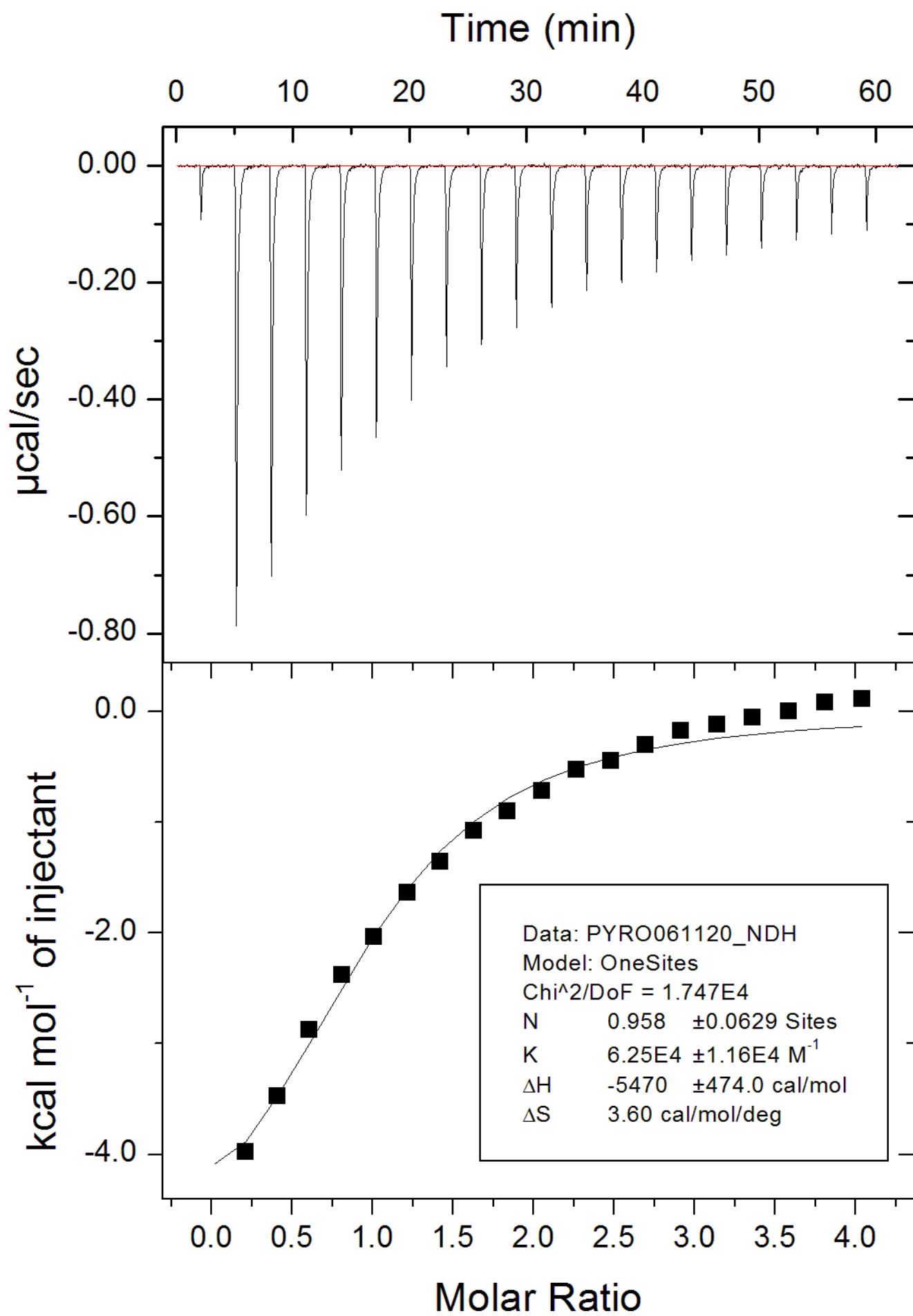

Supplement: Supplementary file 3 — ITC data files. [file 41564_2022_1244_MOESM3_ESM.zip › Variovorax_paradoxus_MarR_73_ligands_SUBMIT/Picloram /Variovorax_paradoxus_MarR_73_Picloram_itc2.pdf]

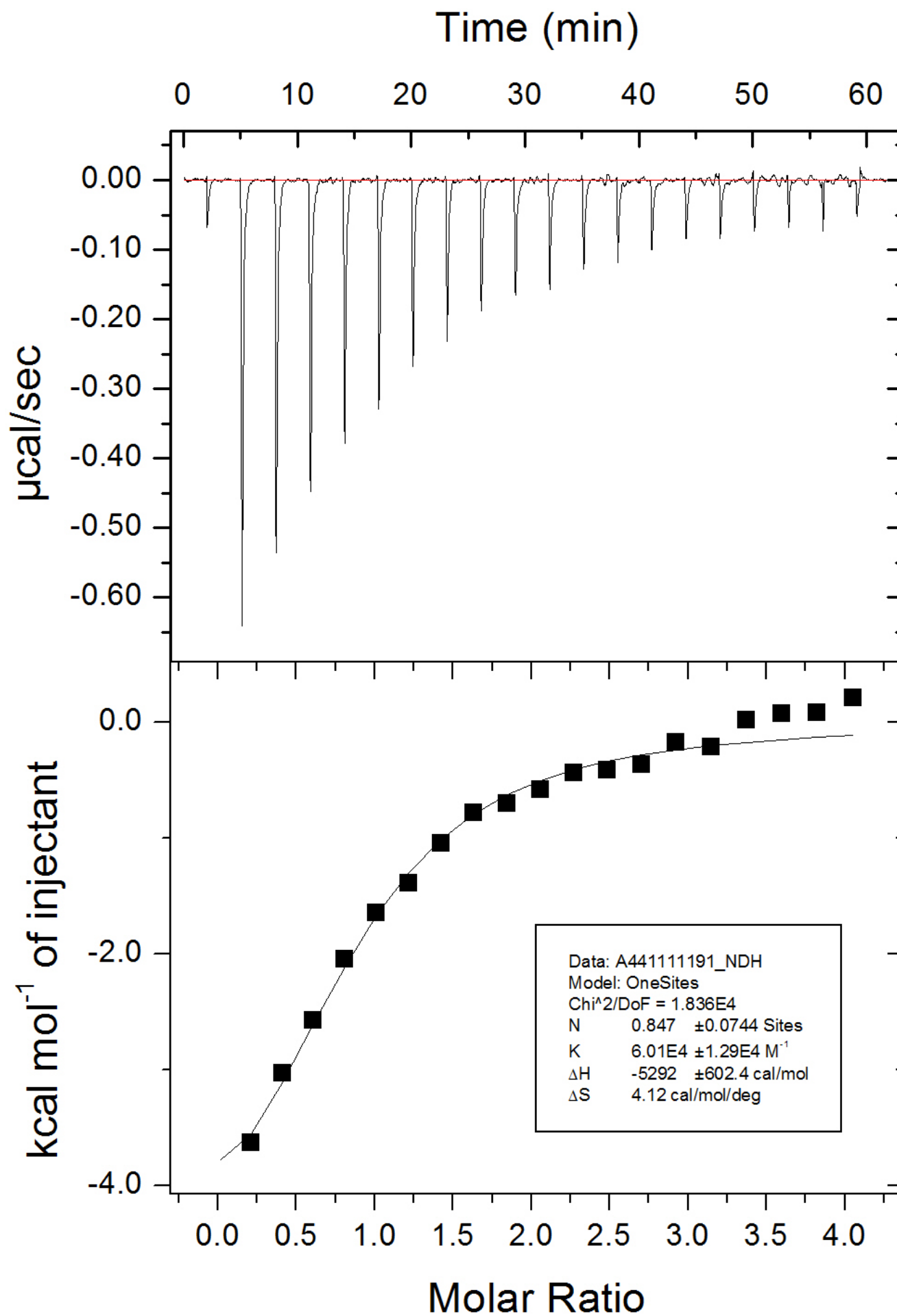

Supplement: Supplementary file 3 — ITC data files. [file 41564_2022_1244_MOESM3_ESM.zip › Variovorax_paradoxus_MarR_73_ligands_SUBMIT/Indole-3-propionic Acid /Variovorax_paradoxus_MarR_73_Indole-3-propionic Acid_itc2.pdf]

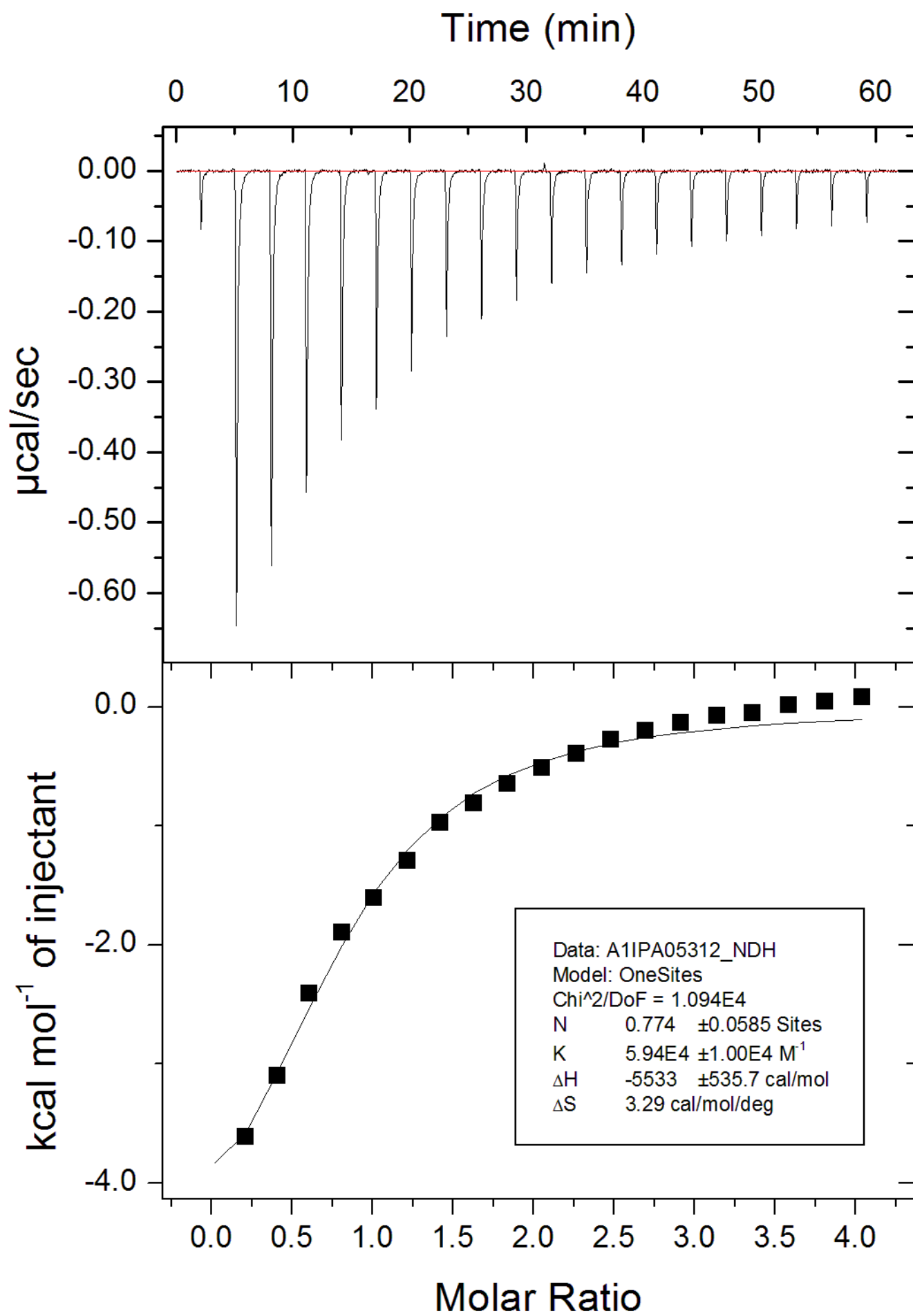

Supplement: Supplementary file 3 — ITC data files. [file 41564_2022_1244_MOESM3_ESM.zip › Variovorax_paradoxus_MarR_73_ligands_SUBMIT/Indole-3-propionic Acid /Variovorax_paradoxus_MarR_73_Indole-3-propionic Acid_itc1.pdf]

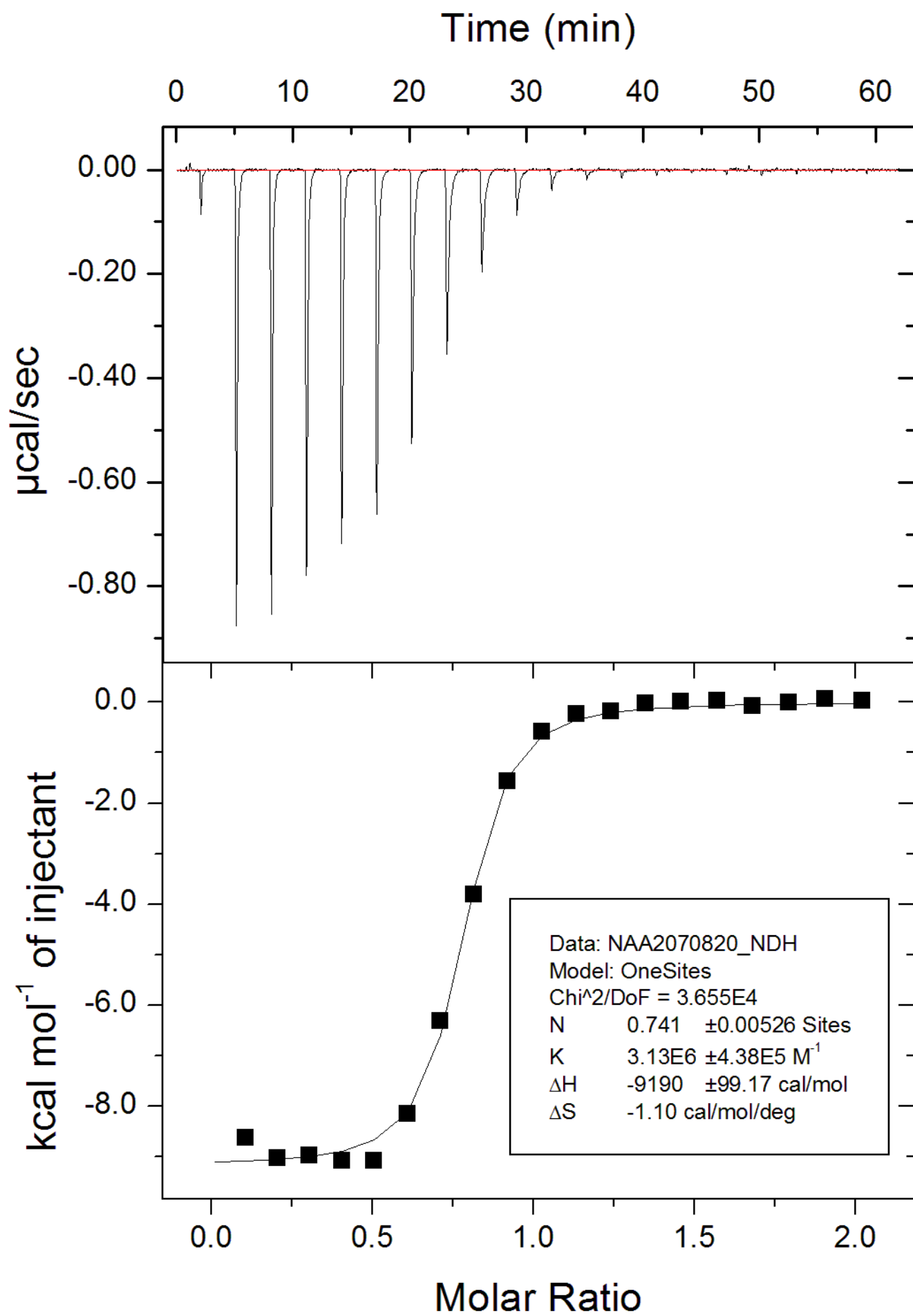

Supplement: Supplementary file 3 — ITC data files. [file 41564_2022_1244_MOESM3_ESM.zip › Variovorax_paradoxus_MarR_73_ligands_SUBMIT/1-Naphthaleneacetic Acid/Variovorax_paradoxus_MarR_73_1-Naphthaleneacetic Acid_itc1.pdf]

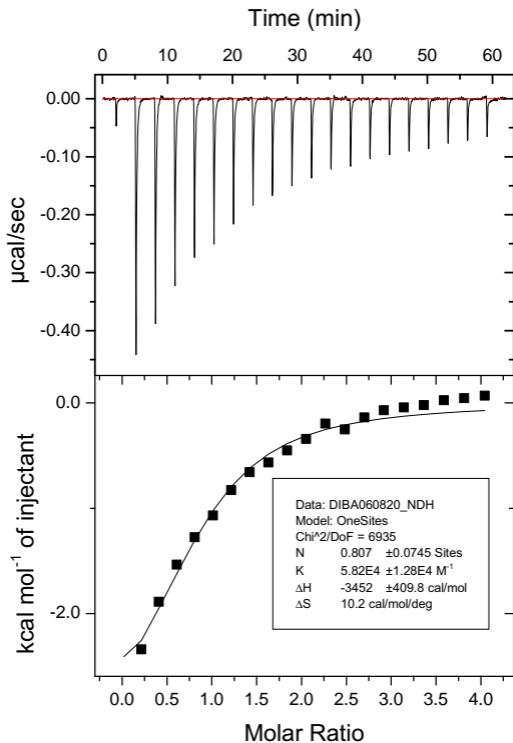

Supplement: Supplementary file 3 — ITC data files. [file 41564_2022_1244_MOESM3_ESM.zip › Variovorax_paradoxus_MarR_73_ligands_SUBMIT/Indole-3-Butyric Acid /Variovorax_paradoxus_MarR_73_Indole-3-Butyric Acid_itc1.PDF]

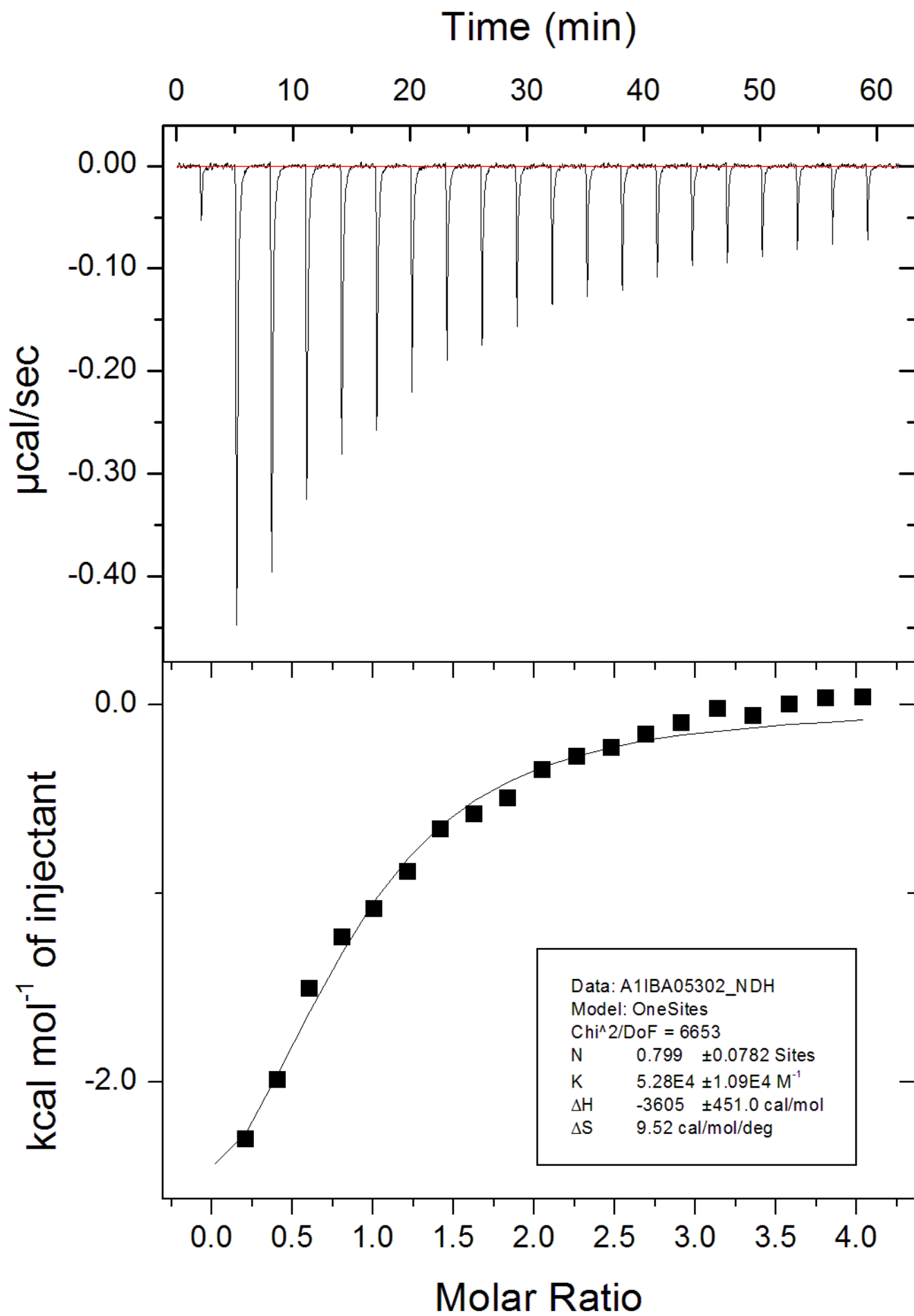

Supplement: Supplementary file 3 — ITC data files. [file 41564_2022_1244_MOESM3_ESM.zip › Variovorax_paradoxus_MarR_73_ligands_SUBMIT/Indole-3-Butyric Acid /Variovorax_paradoxus_MarR_73_Indole-3-Butyric Acid_itc2.pdf]

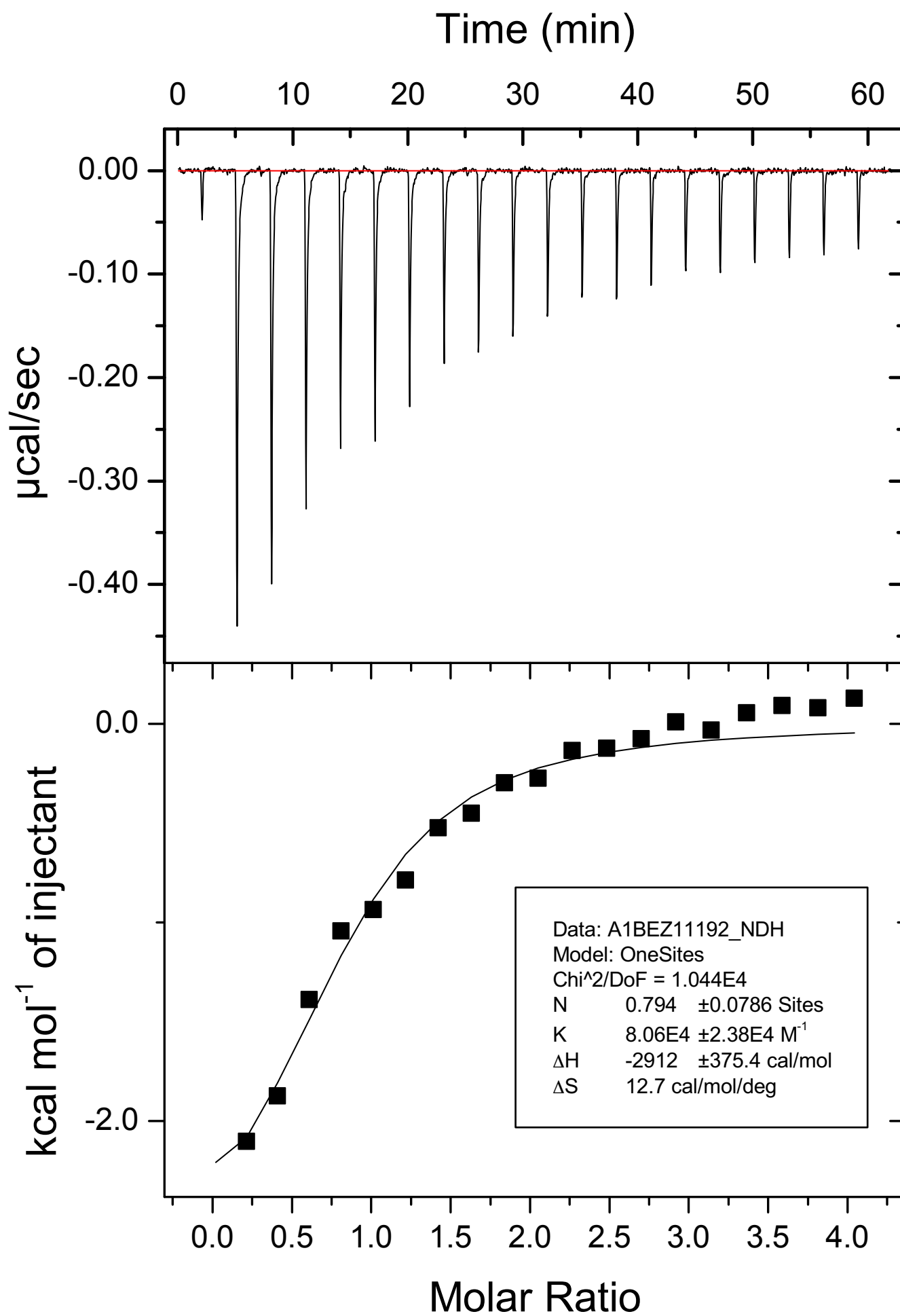

Supplement: Supplementary file 3 — ITC data files. [file 41564_2022_1244_MOESM3_ESM.zip › Variovorax_paradoxus_MarR_73_ligands_SUBMIT/Benzoic Acid /Variovorax_paradoxus_MarR_73_Benzoic Acid_itc2.pdf]

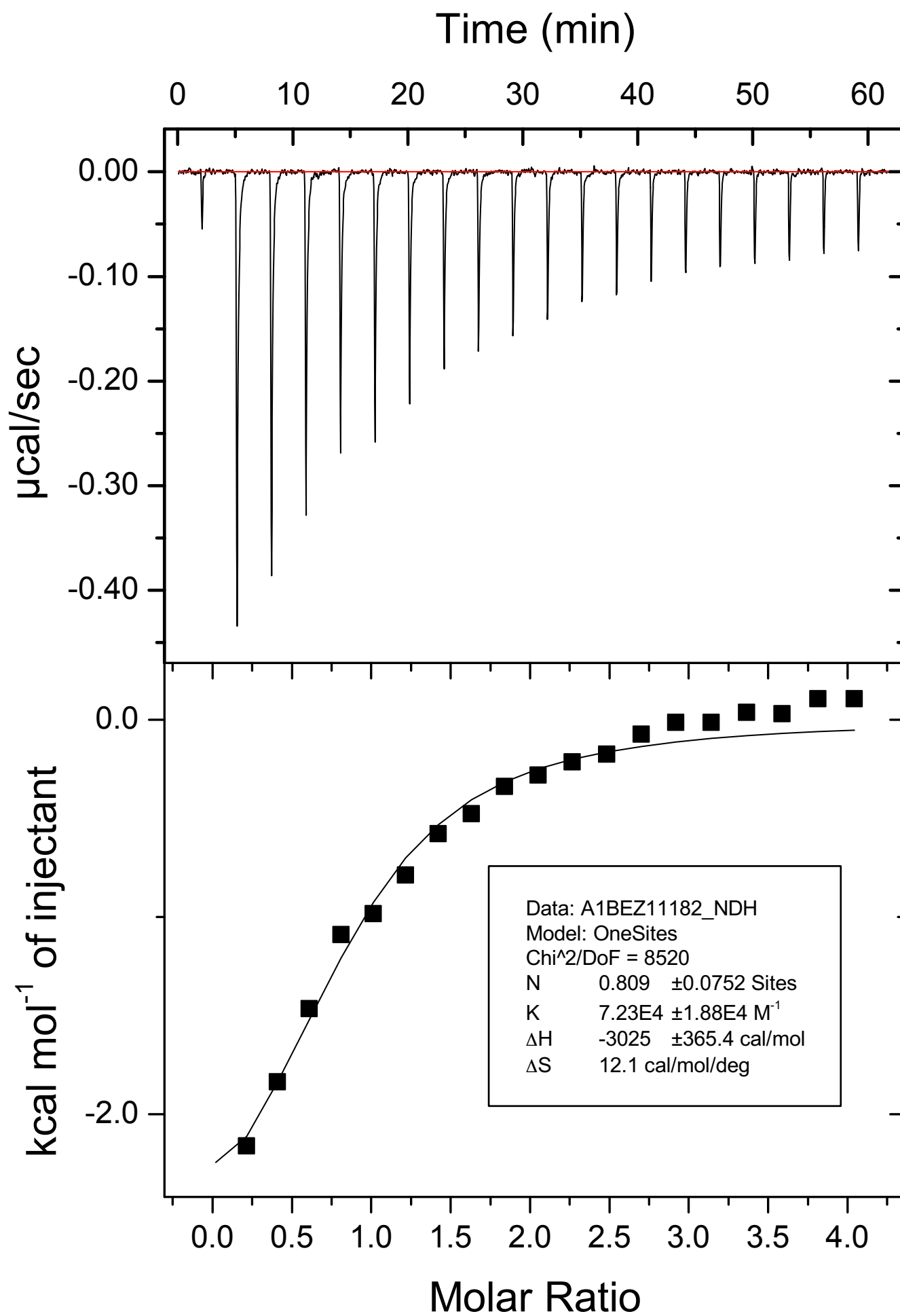

Supplement: Supplementary file 3 — ITC data files. [file 41564_2022_1244_MOESM3_ESM.zip › Variovorax_paradoxus_MarR_73_ligands_SUBMIT/Benzoic Acid /Variovorax_paradoxus_MarR_73_Benzoic Acid_itc1.pdf]

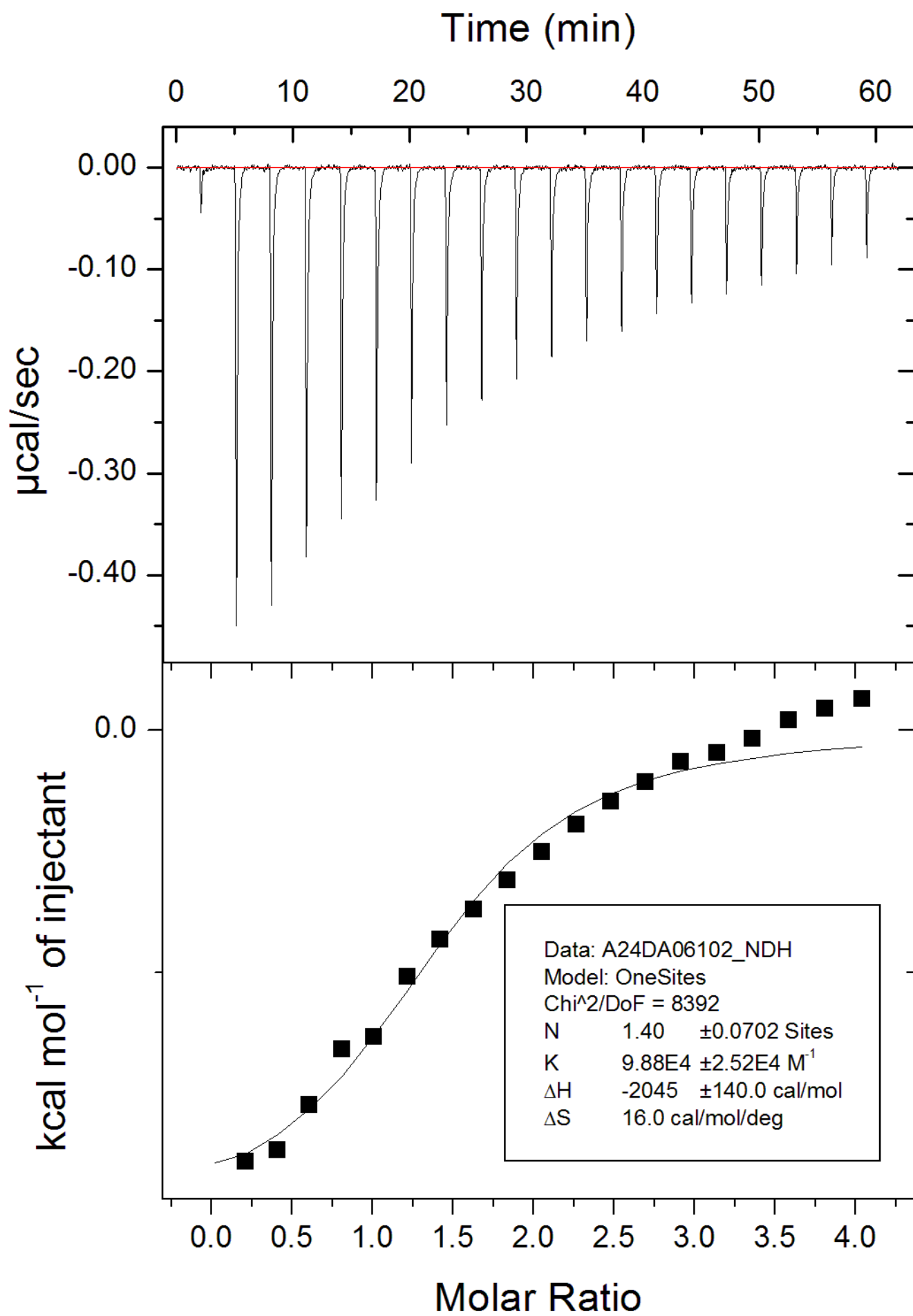

Supplement: Supplementary file 3 — ITC data files. [file 41564_2022_1244_MOESM3_ESM.zip › Variovorax_paradoxus_MarR_73_ligands_SUBMIT/2,4-Dichorophenoxyacetic Acid /Variovorax_paradoxus_MarR_73_2,4-Dichorophenoxyacetic Acid_itc1.pdf]

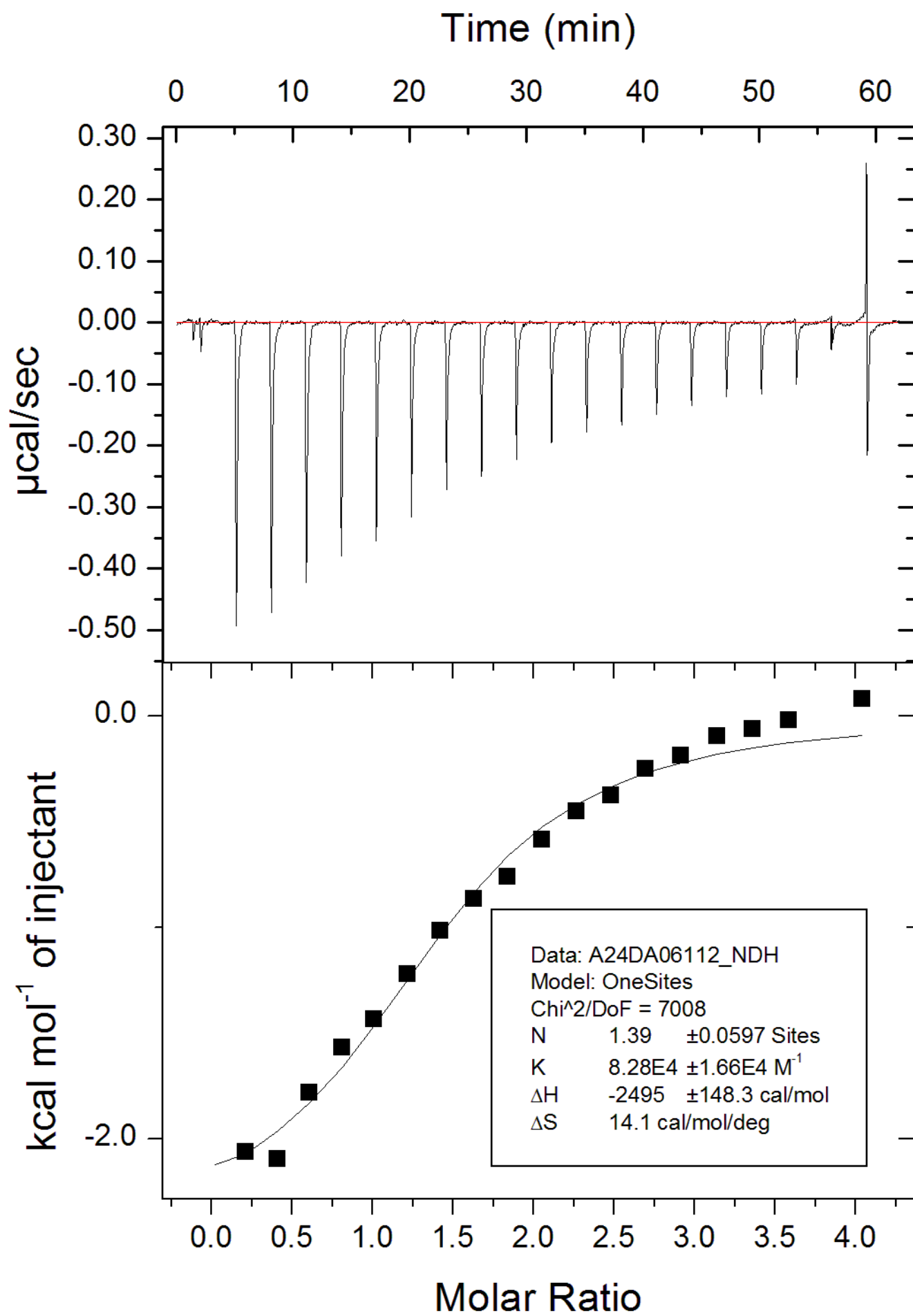

Supplement: Supplementary file 3 — ITC data files. [file 41564_2022_1244_MOESM3_ESM.zip › Variovorax_paradoxus_MarR_73_ligands_SUBMIT/2,4-Dichorophenoxyacetic Acid /Variovorax_paradoxus_MarR_73_2,4-Dichorophenoxyacetic Acid_itc2.pdf]

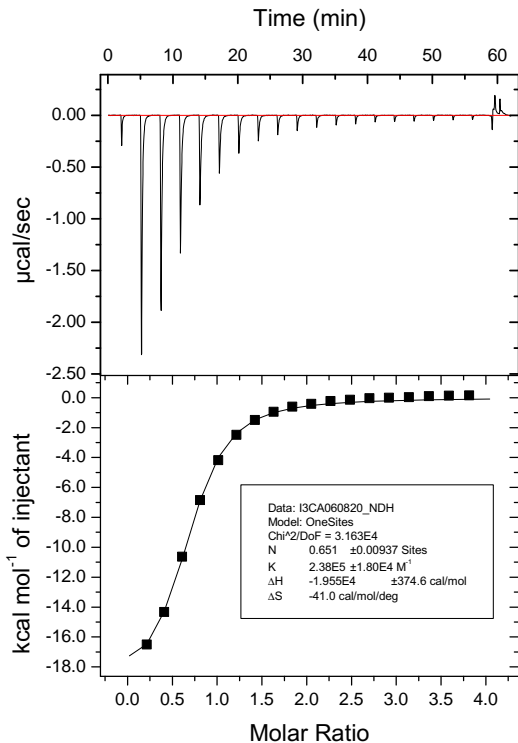

Supplement: Supplementary file 3 — ITC data files. [file 41564_2022_1244_MOESM3_ESM.zip › Variovorax_paradoxus_MarR_73_ligands_SUBMIT/Indole-3-Carboxylic Acid/Variovorax_paradoxus_MarR_73_Indole-3-Carboxylic Acid_itc1.PDF]

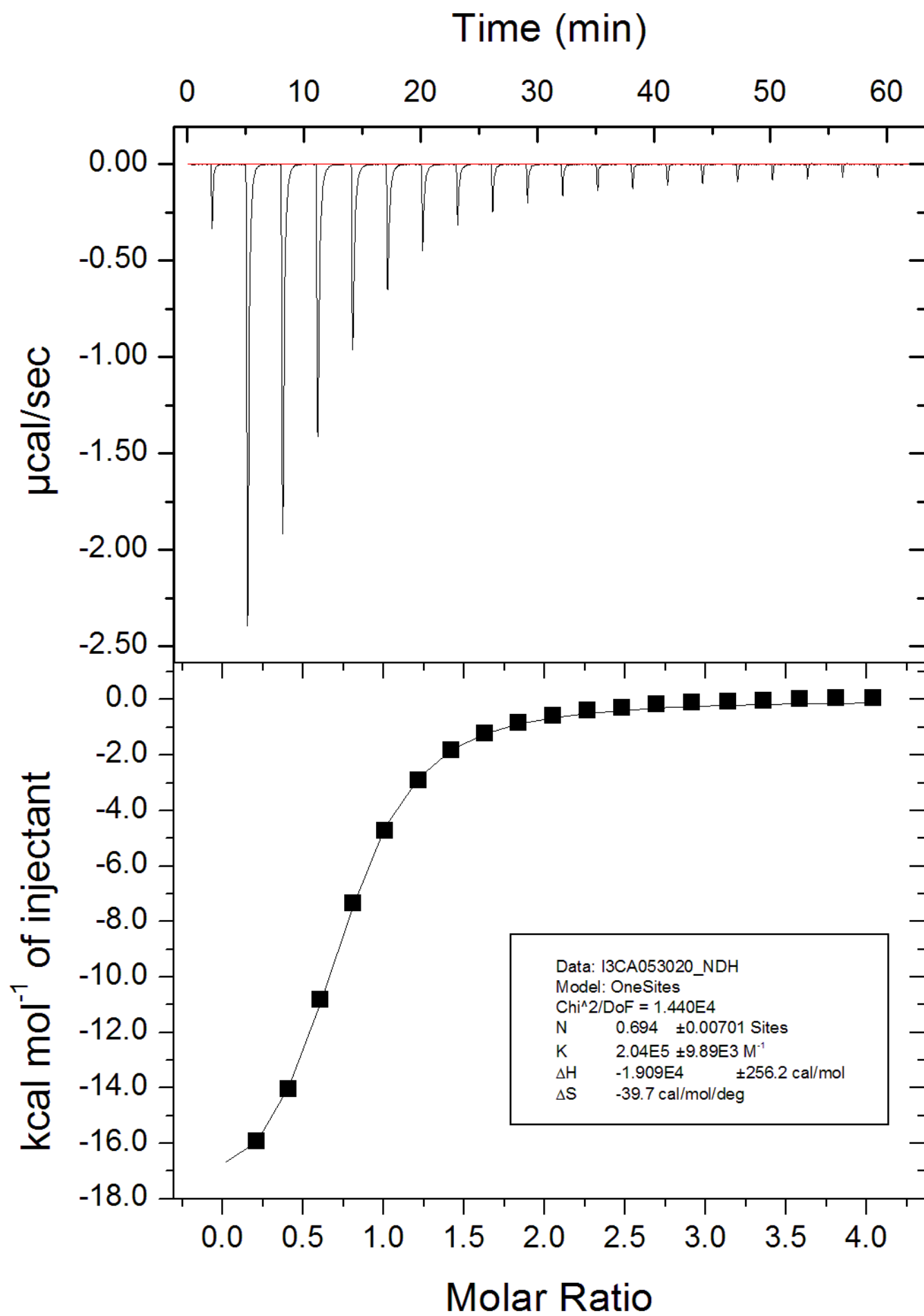

Supplement: Supplementary file 3 — ITC data files. [file 41564_2022_1244_MOESM3_ESM.zip › Variovorax_paradoxus_MarR_73_ligands_SUBMIT/Indole-3-Carboxylic Acid/Variovorax_paradoxus_MarR_73_Indole-3-Carboxylic Acid_itc2.pdf]

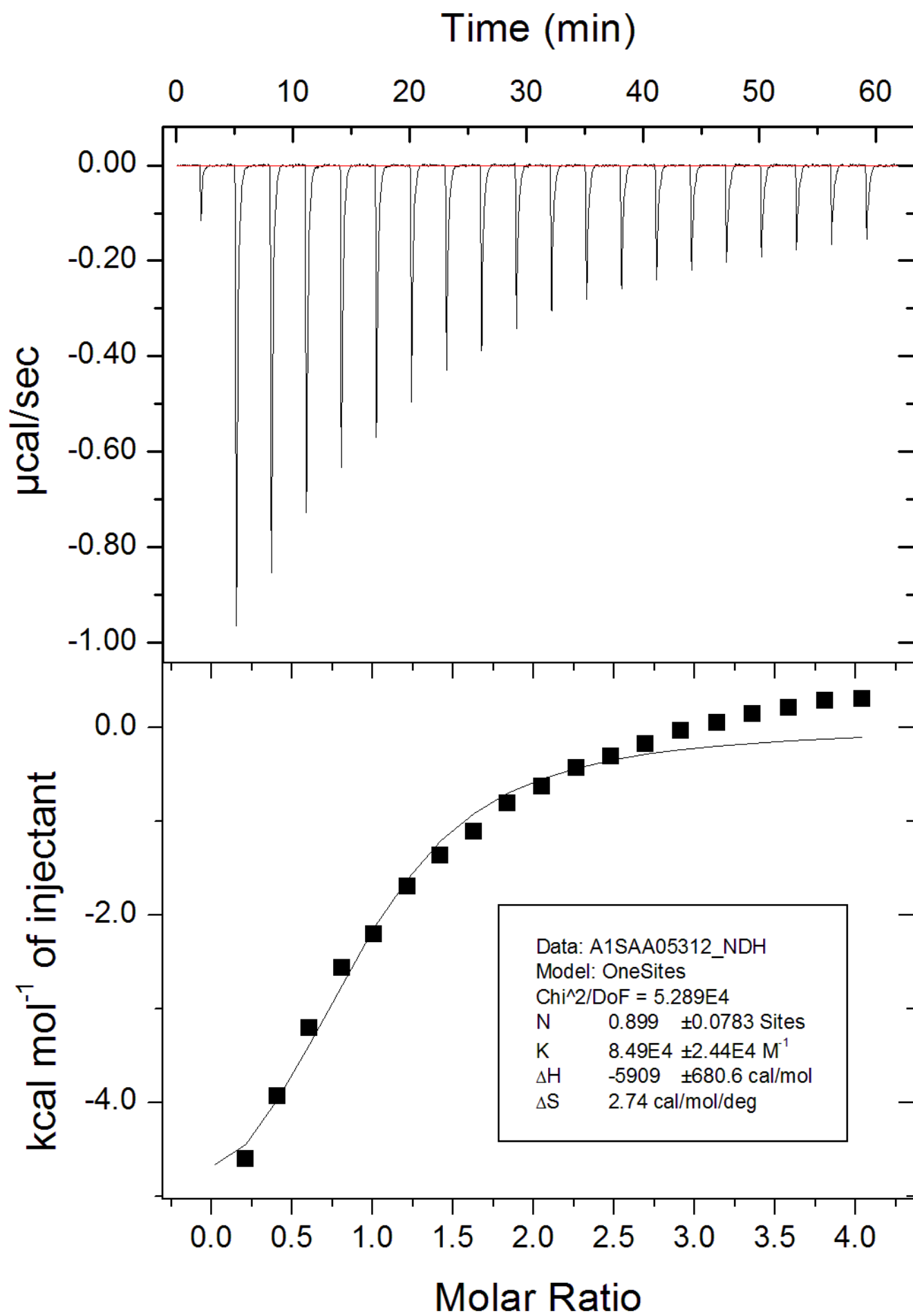

Supplement: Supplementary file 3 — ITC data files. [file 41564_2022_1244_MOESM3_ESM.zip › Variovorax_paradoxus_MarR_73_ligands_SUBMIT/Salicylic Acid /Variovorax_paradoxus_MarR_73_Salicylic Acid_itc2.pdf]

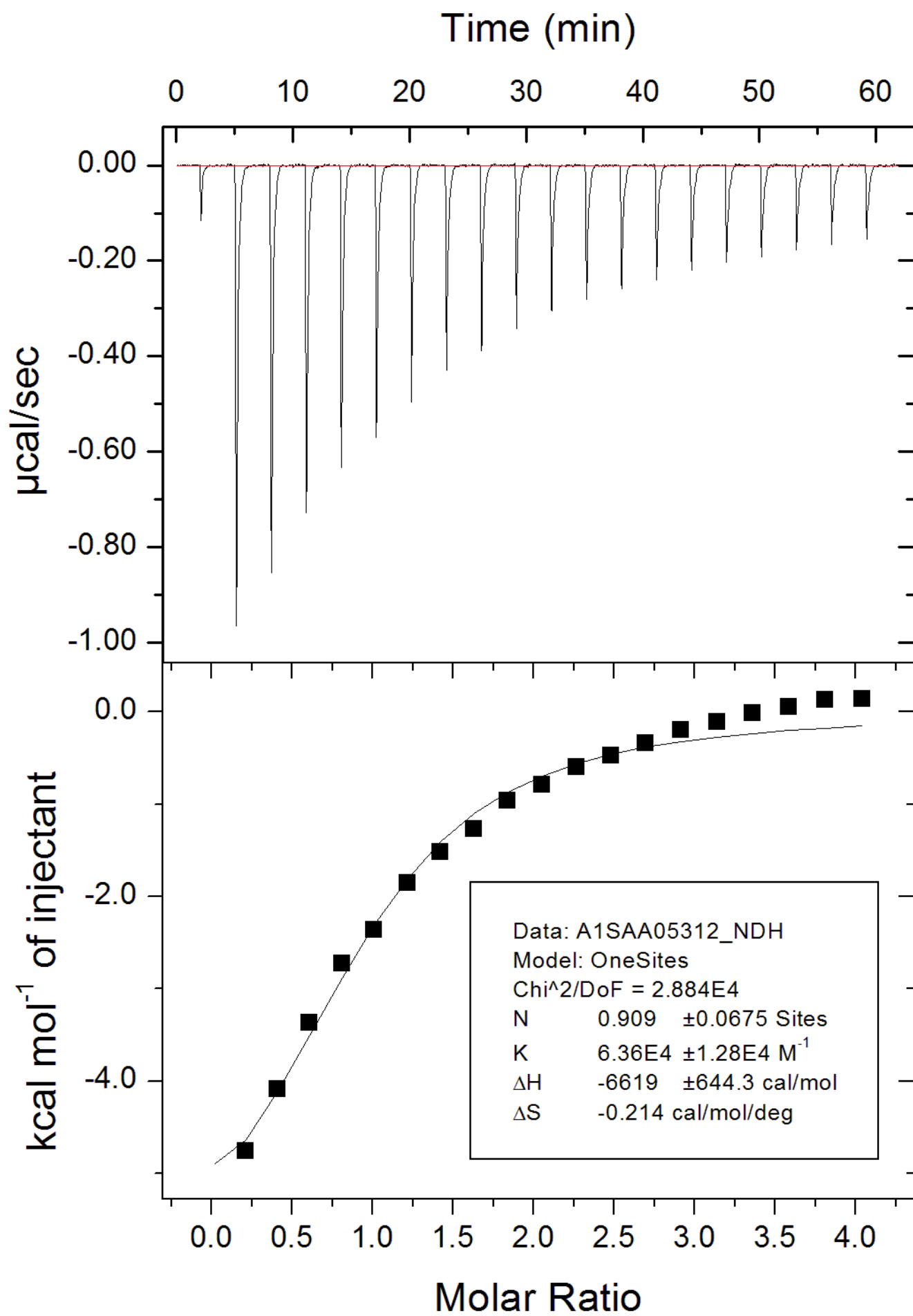

Supplement: Supplementary file 3 — ITC data files. [file 41564_2022_1244_MOESM3_ESM.zip › Variovorax_paradoxus_MarR_73_ligands_SUBMIT/Salicylic Acid /Variovorax_paradoxus_MarR_73_Salicylic Acid_itc1.pdf]

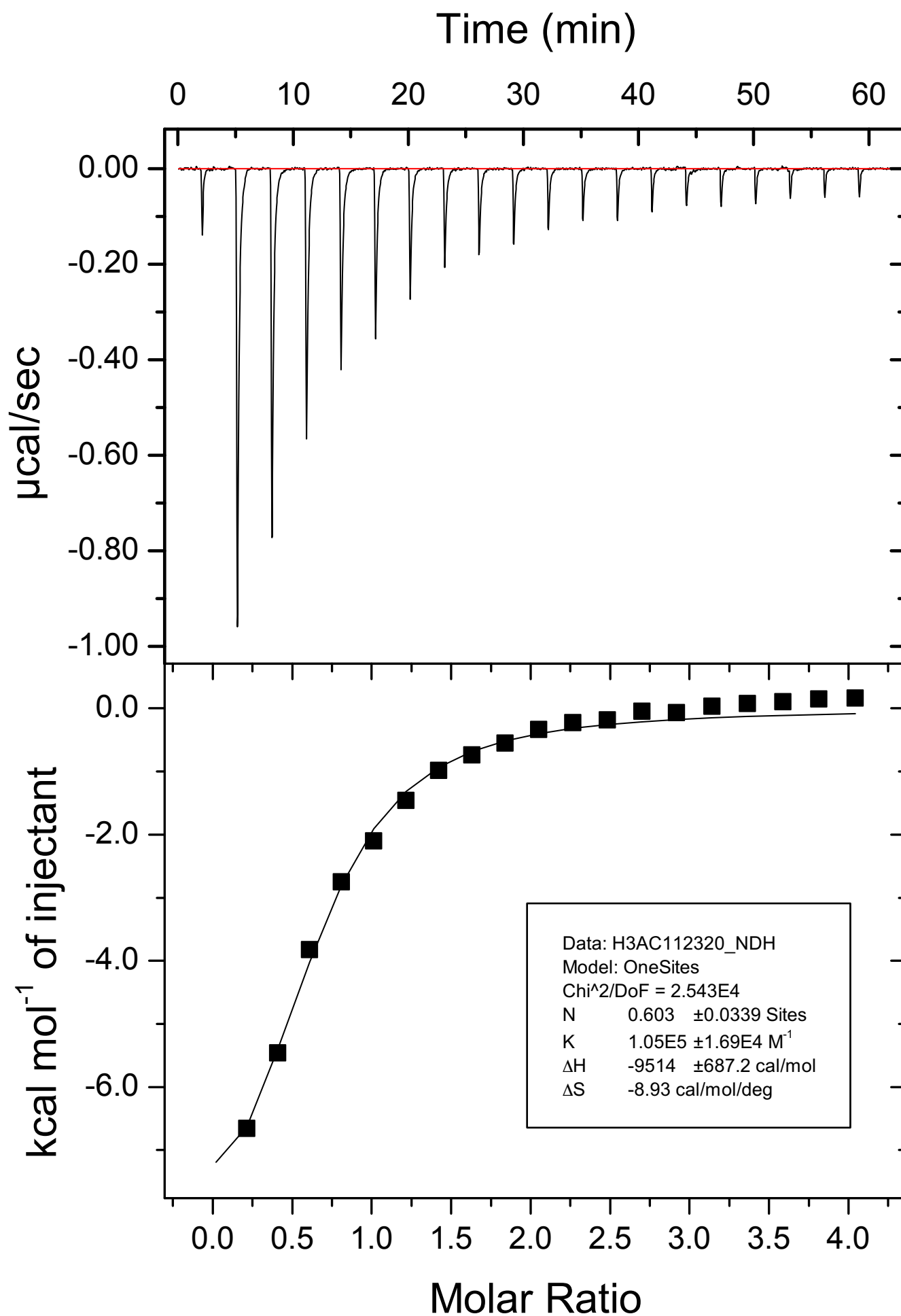

Supplement: Supplementary file 3 — ITC data files. [file 41564_2022_1244_MOESM3_ESM.zip › Variovorax_paradoxus_MarR_73_ligands_SUBMIT/5-Hydroxyindole-3-Acetic Acid /Variovorax_paradoxus_MarR_73_5-Hydroxyindole-3-Acetic Acid_itc1.pdf]

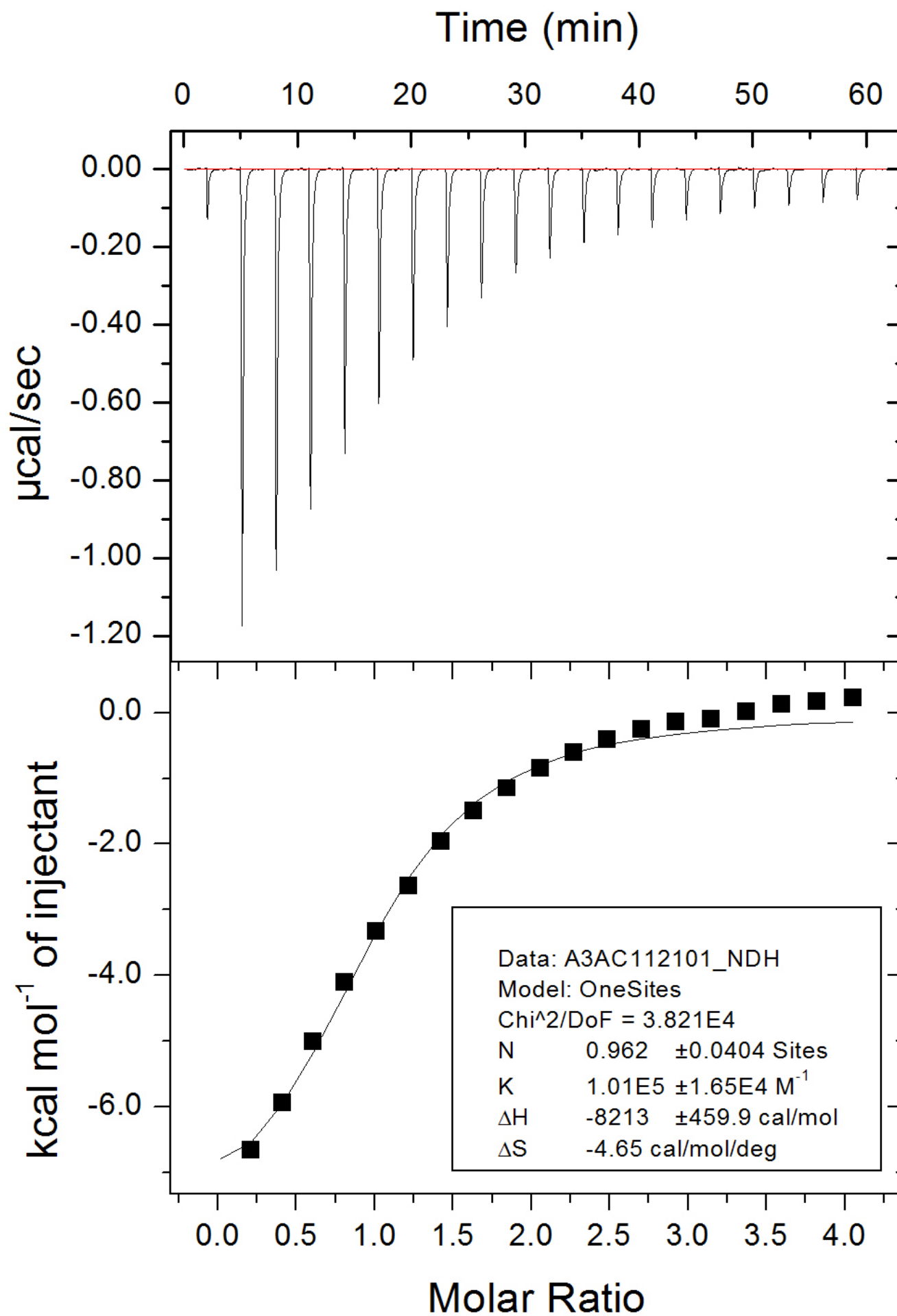

Supplement: Supplementary file 3 — ITC data files. [file 41564_2022_1244_MOESM3_ESM.zip › Variovorax_paradoxus_MarR_73_ligands_SUBMIT/5-Hydroxyindole-3-Acetic Acid /Variovorax_paradoxus_MarR_73_5-Hydroxyindole-3-Acetic Acid_itc2.pdf]

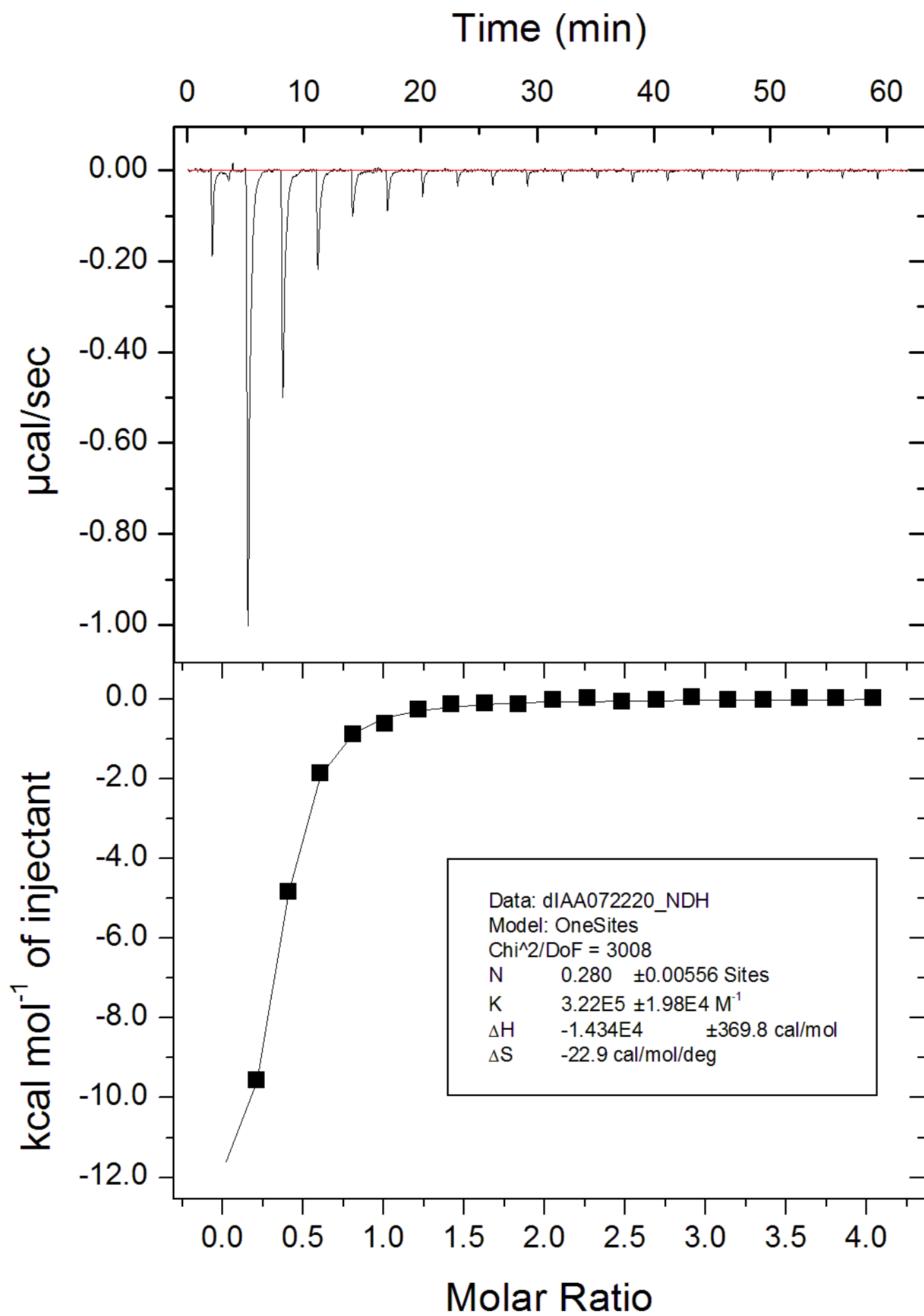

Supplement: Supplementary file 4 — ITC data files. [file 41564_2022_1244_MOESM4_ESM.zip › Variovorax_paradoxus_MarR_73_mutants_IAA_SUBMIT /H32A_IAA/H32A_IAA_itc1.pdf]

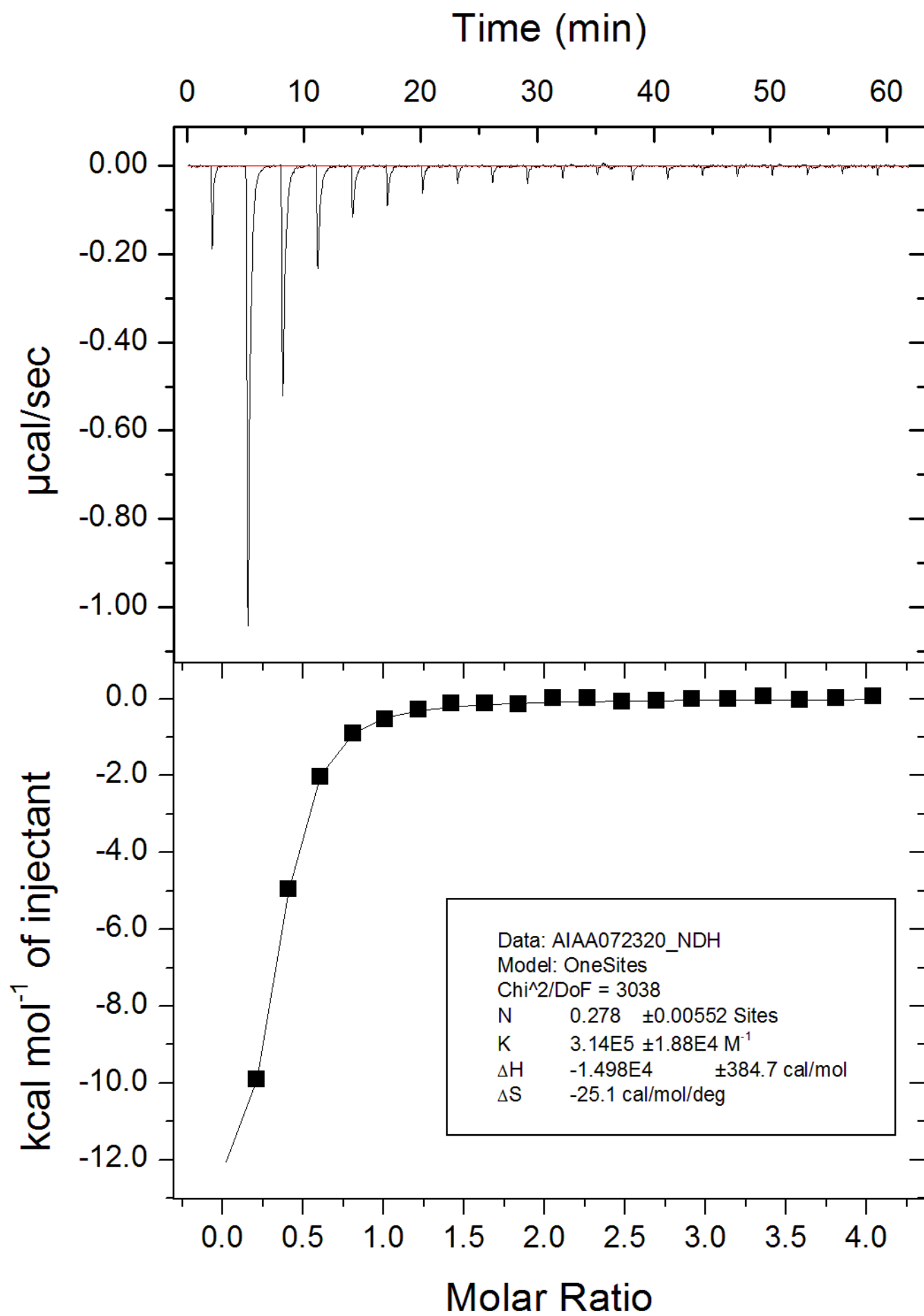

Supplement: Supplementary file 4 — ITC data files. [file 41564_2022_1244_MOESM4_ESM.zip › Variovorax_paradoxus_MarR_73_mutants_IAA_SUBMIT /H32A_IAA/H32A_IAA_itc2.pdf]

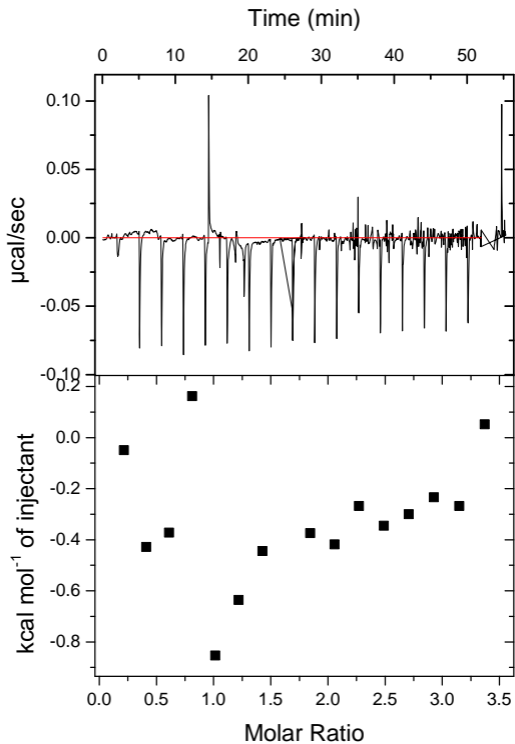

Supplement: Supplementary file 4 — ITC data files. [file 41564_2022_1244_MOESM4_ESM.zip › Variovorax_paradoxus_MarR_73_mutants_IAA_SUBMIT /S28AH32AR46A_IAA/S28AH32AR46A_IAA_itc2.PDF]

Time (min)

0 10 20 30 40 50 60

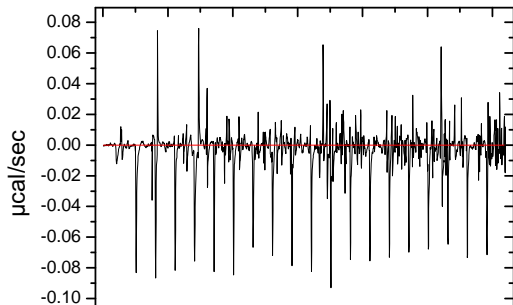

kcal mol<sup>-1</sup> of injectant

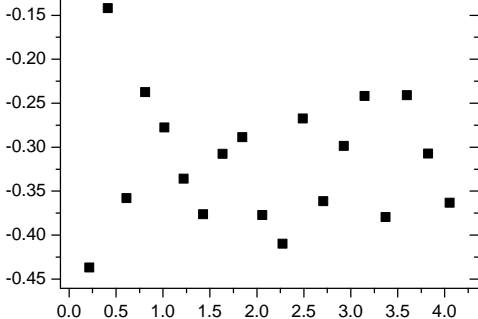

Molar Ratio

Supplement: Supplementary file 4 — ITC data files. [file 41564_2022_1244_MOESM4_ESM.zip › Variovorax_paradoxus_MarR_73_mutants_IAA_SUBMIT /S28AH32AR46A_IAA/S28AH32AR46A_IAA_itc1.PDF]

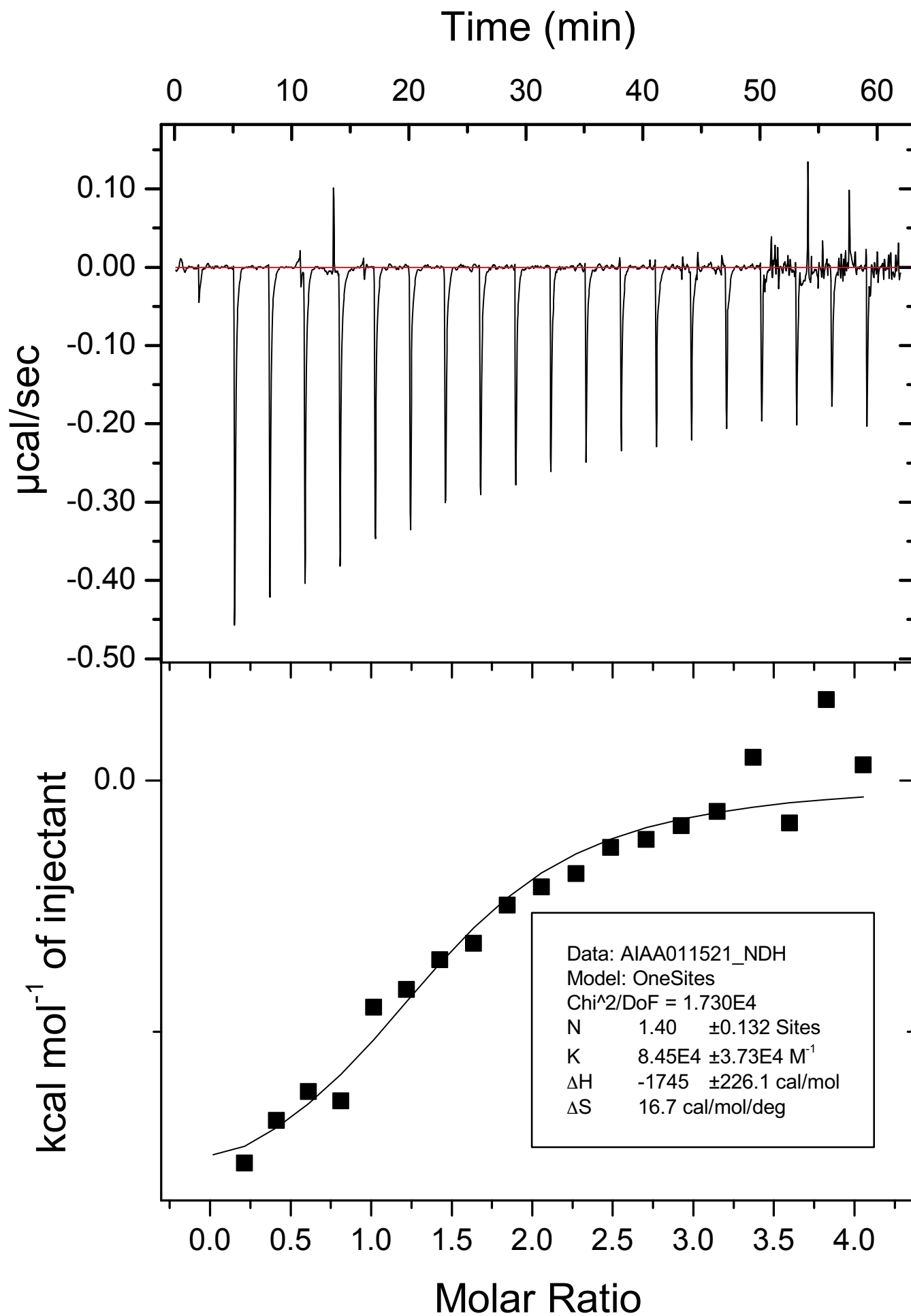

Supplement: Supplementary file 4 — ITC data files. [file 41564_2022_1244_MOESM4_ESM.zip › Variovorax_paradoxus_MarR_73_mutants_IAA_SUBMIT /S28AR46A_IAA/S28AR46A_IAA_itc1.pdf]

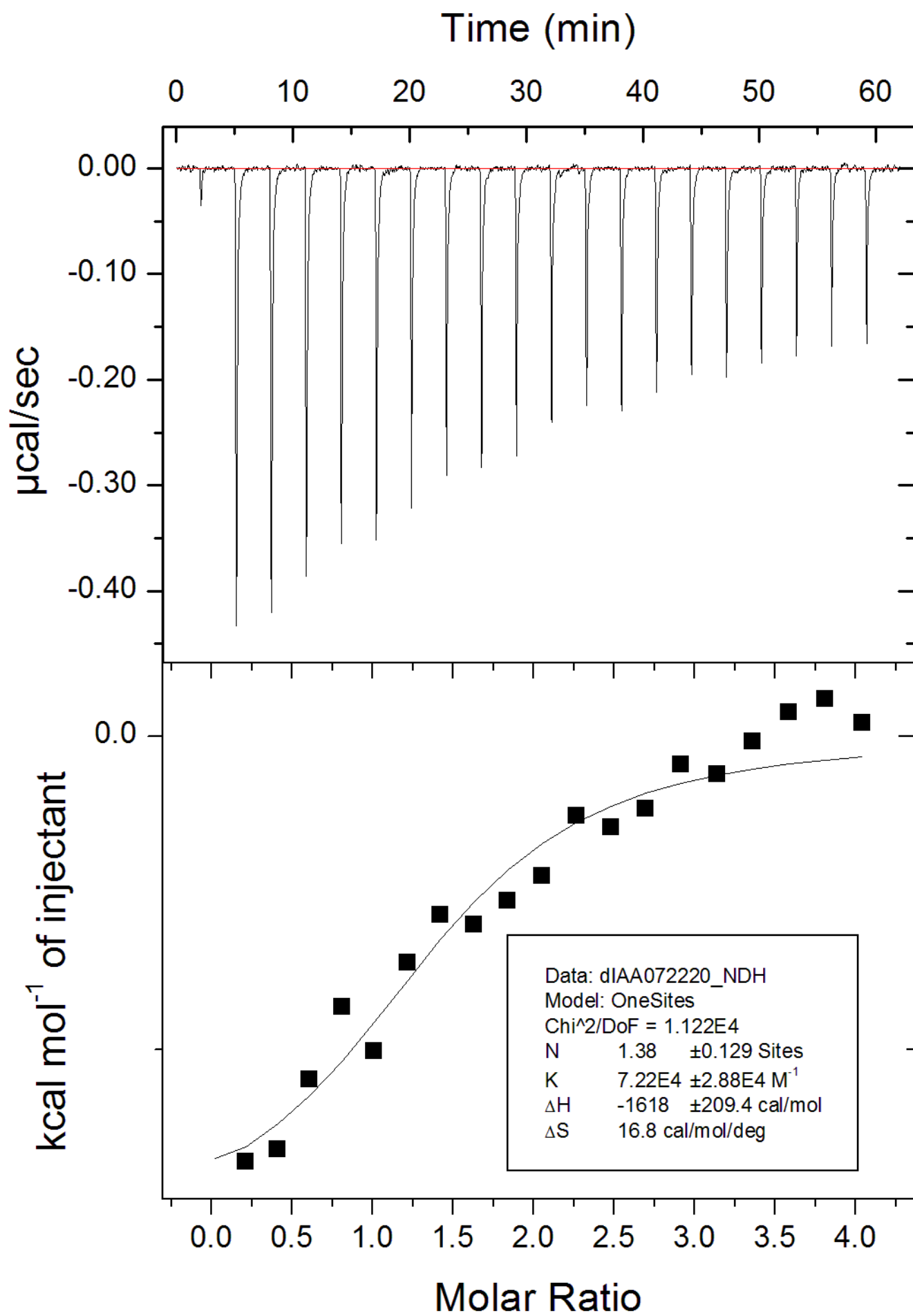

Supplement: Supplementary file 4 — ITC data files. [file 41564_2022_1244_MOESM4_ESM.zip › Variovorax_paradoxus_MarR_73_mutants_IAA_SUBMIT /S28AR46A_IAA/S28AR46A_IAA_itc2.pdf]

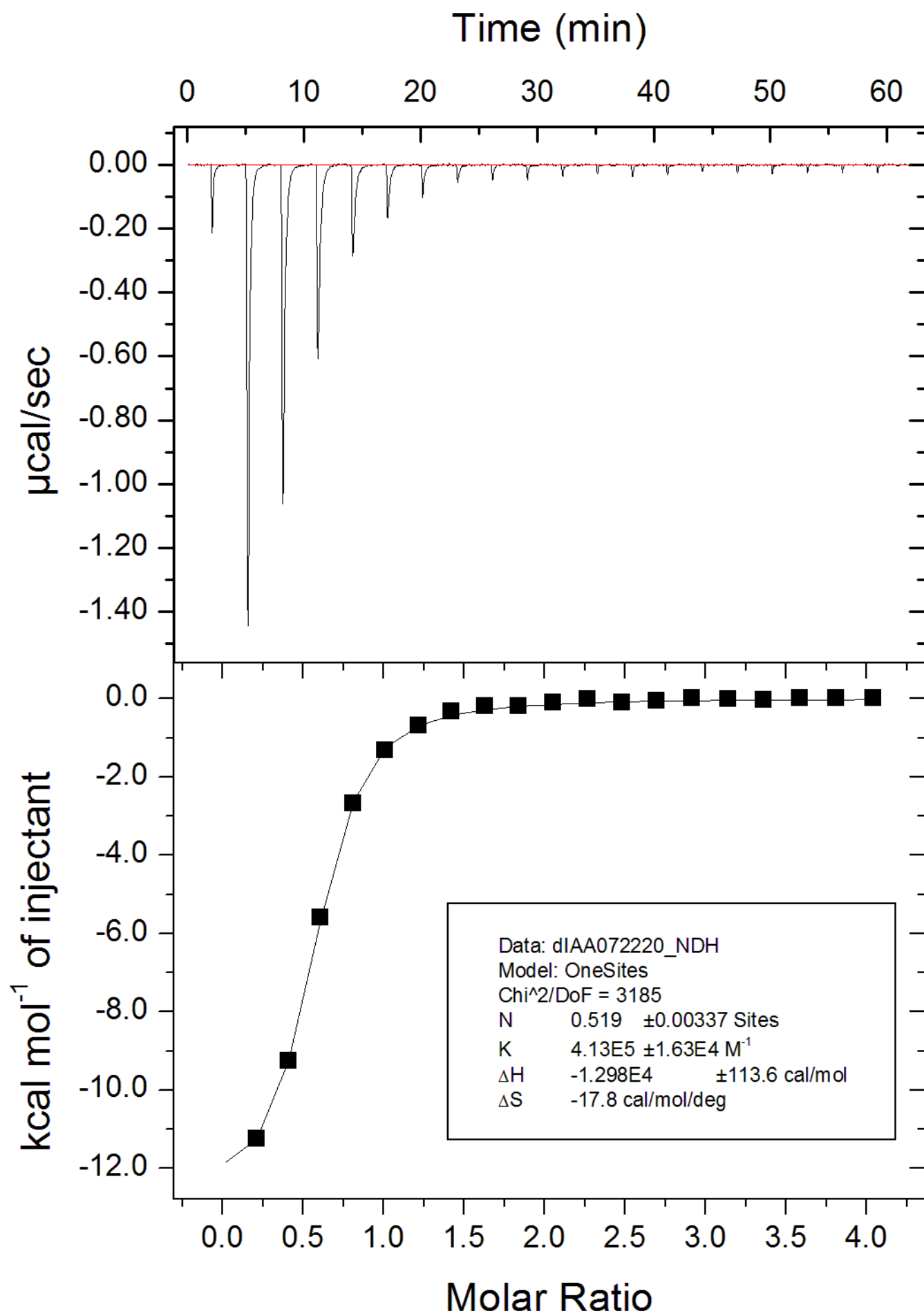

Supplement: Supplementary file 4 — ITC data files. [file 41564_2022_1244_MOESM4_ESM.zip › Variovorax_paradoxus_MarR_73_mutants_IAA_SUBMIT /S28A_IAA/S28A_IAA_itc1.pdf]

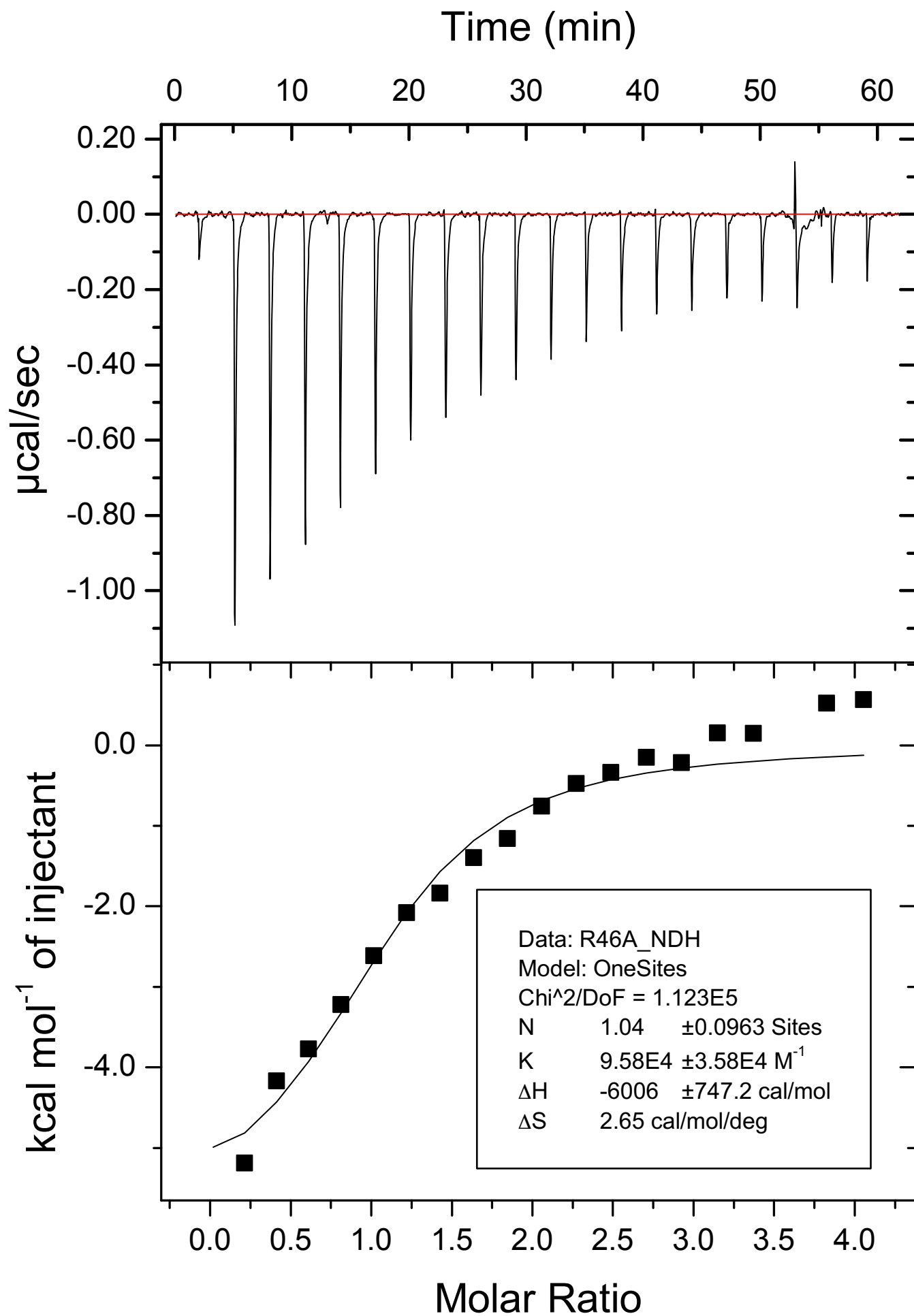

Supplement: Supplementary file 4 — ITC data files. [file 41564_2022_1244_MOESM4_ESM.zip › Variovorax_paradoxus_MarR_73_mutants_IAA_SUBMIT /R46A_IAA/R46A_IAA_itc1.pdf]

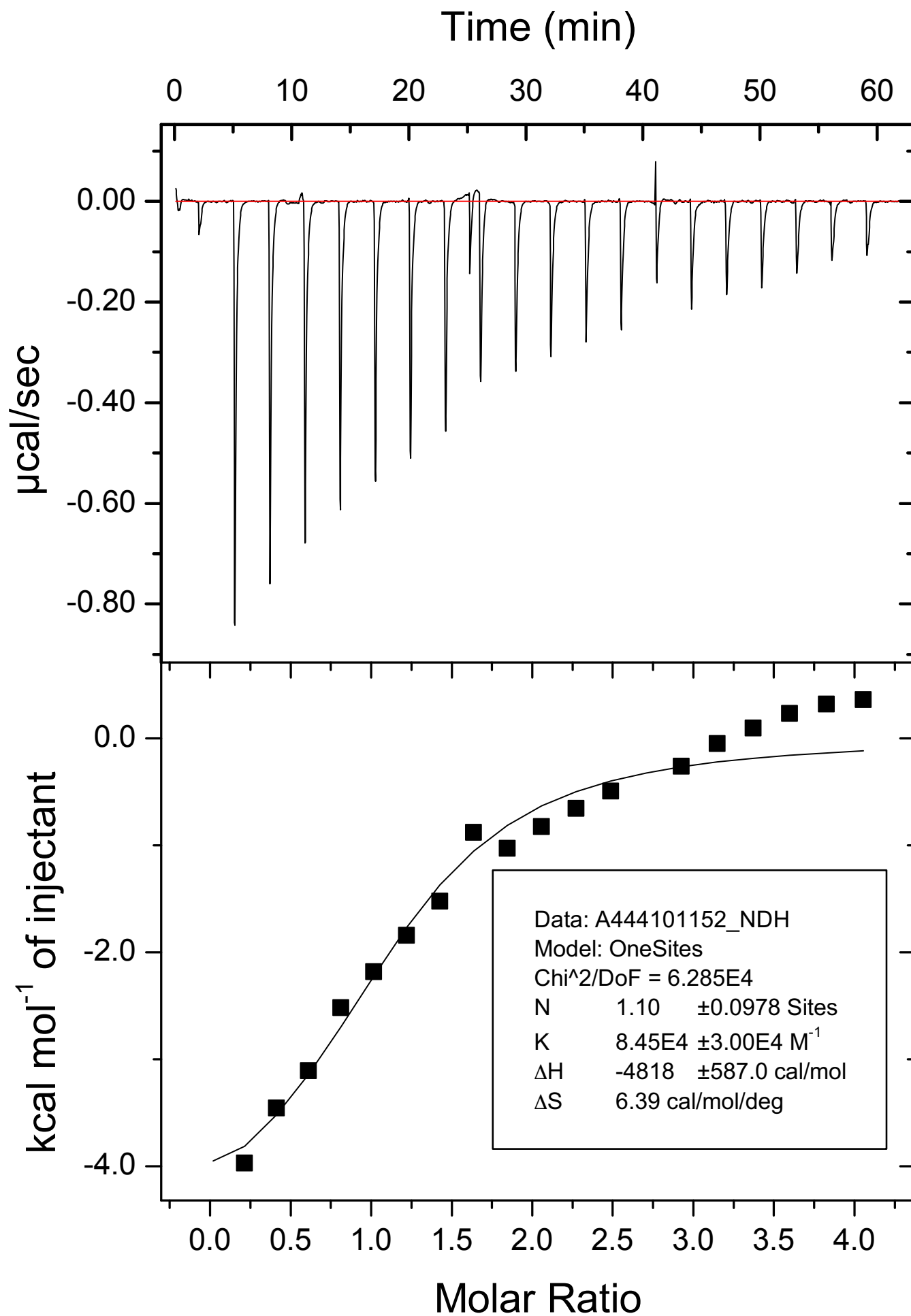

Supplement: Supplementary file 4 — ITC data files. [file 41564_2022_1244_MOESM4_ESM.zip › Variovorax_paradoxus_MarR_73_mutants_IAA_SUBMIT /R46A_IAA/R46A_IAA_itc2.pdf]

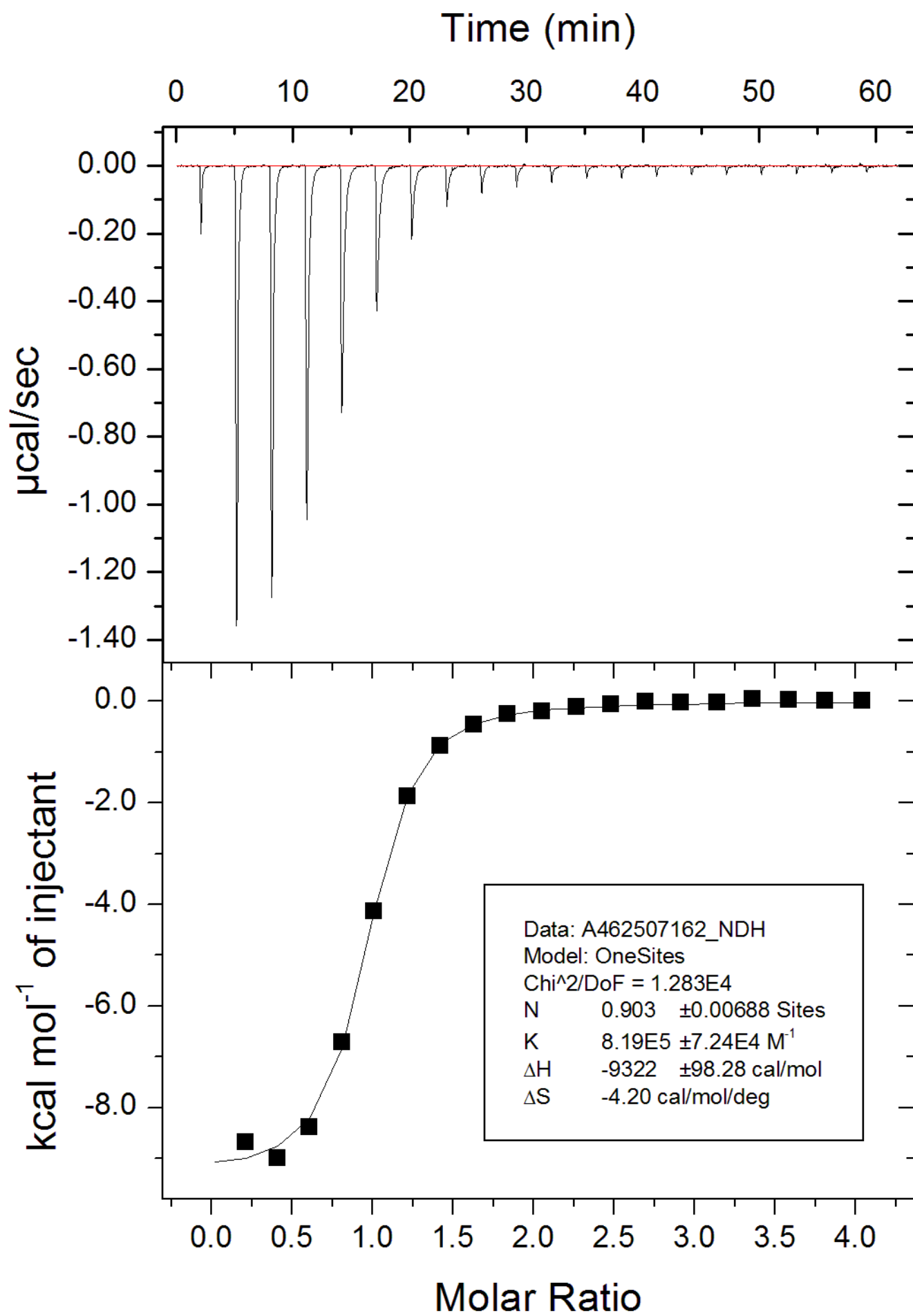

Supplement: Supplementary file 5 — ITC data files. [file 41564_2022_1244_MOESM5_ESM.zip › Homologs_SUBMIT/Acinetobacter baumannii (UNC462-5)/Acinetobacter baumannii_itc1.pdf]

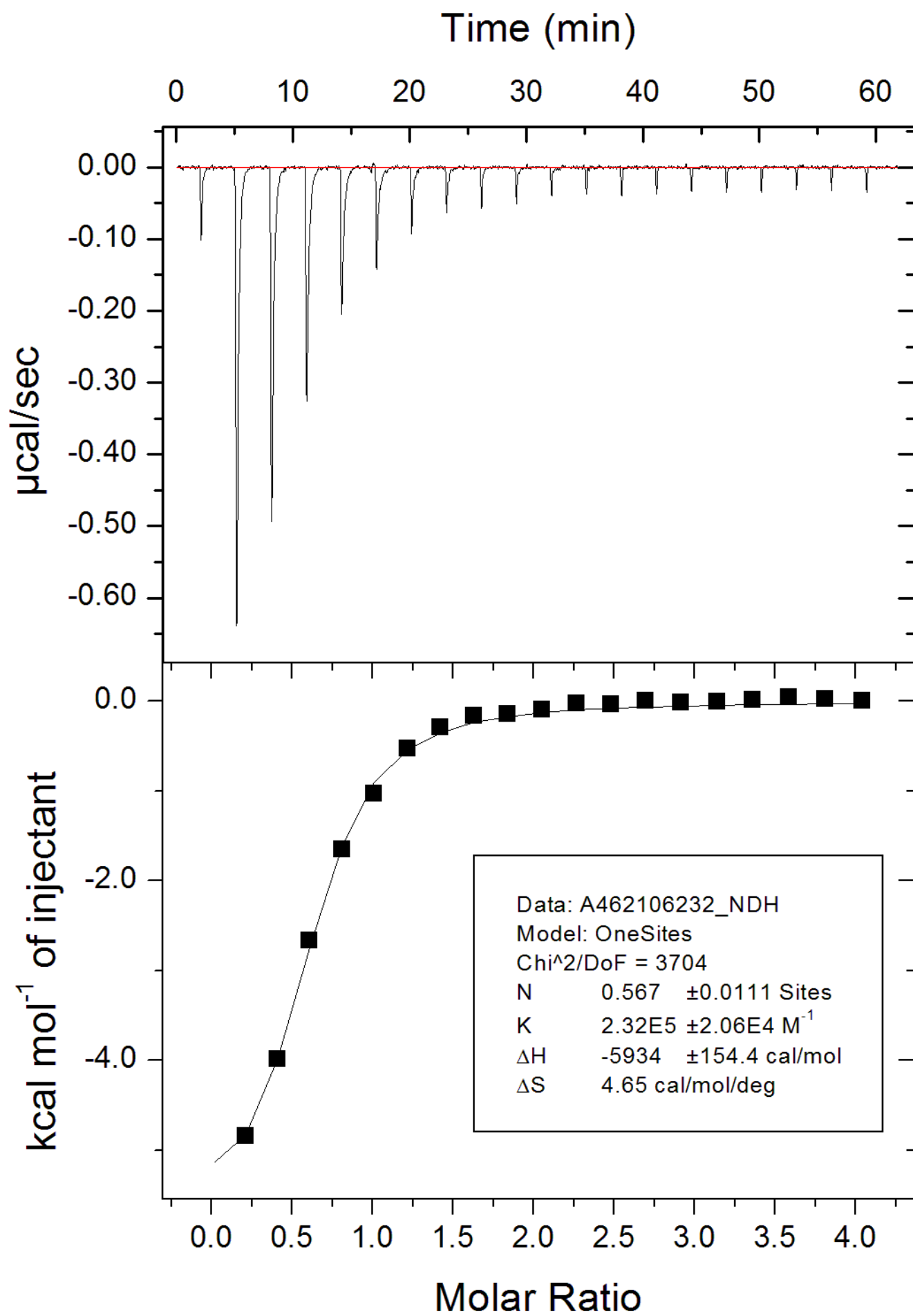

Supplement: Supplementary file 5 — ITC data files. [file 41564_2022_1244_MOESM5_ESM.zip › Homologs_SUBMIT/Pseudomonas putida (UNC462-1)/Pseudomonas putida_itc1.pdf]

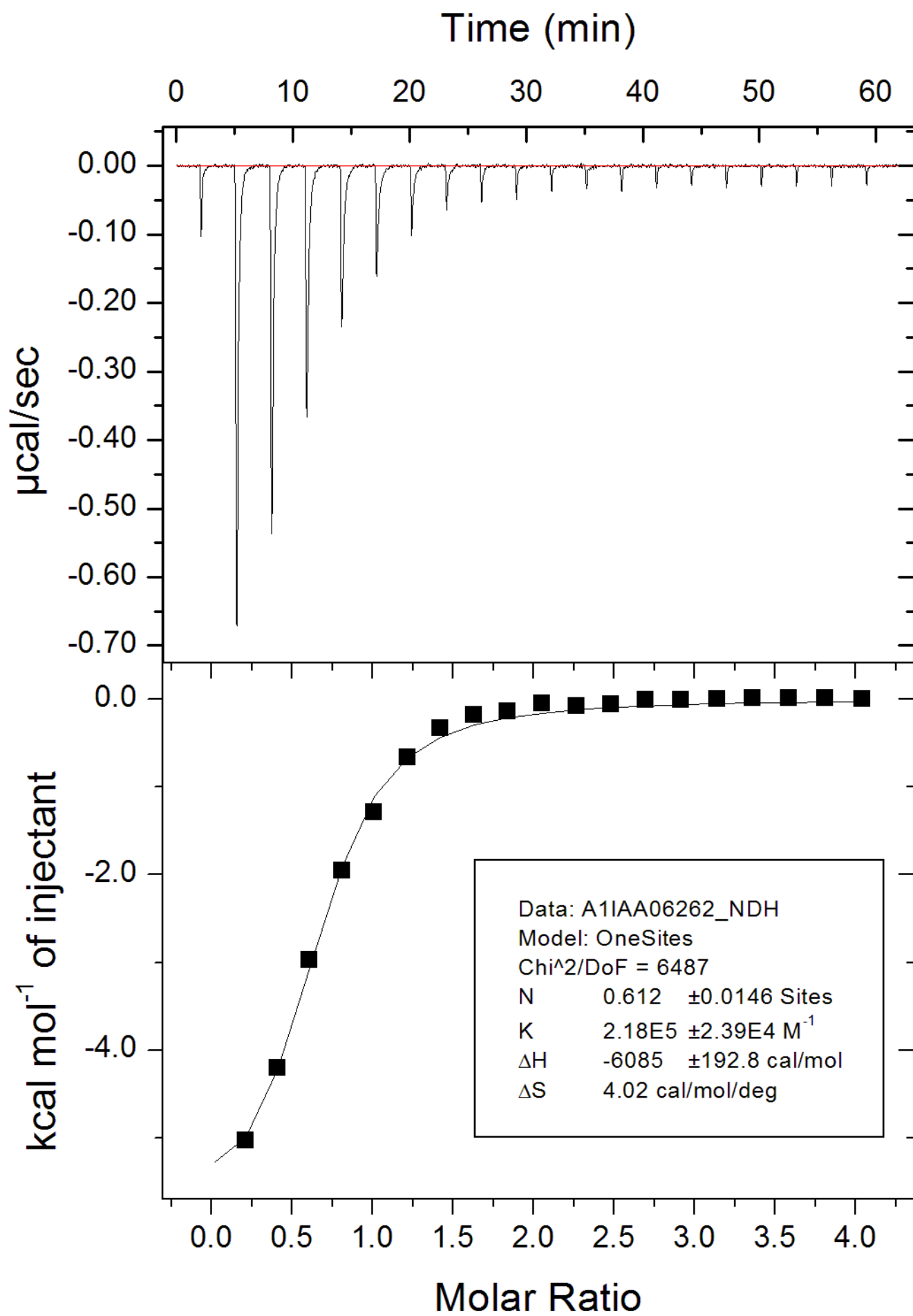

Supplement: Supplementary file 5 — ITC data files. [file 41564_2022_1244_MOESM5_ESM.zip › Homologs_SUBMIT/Pseudomonas putida (UNC462-1)/Pseudomonas putida_itc2.pdf]

Time (min)

0 10 20 30 40 50 60

0.00

$\mu\text{cal/sec}$

$\text{kcal mol}^{-1}$  of injectant

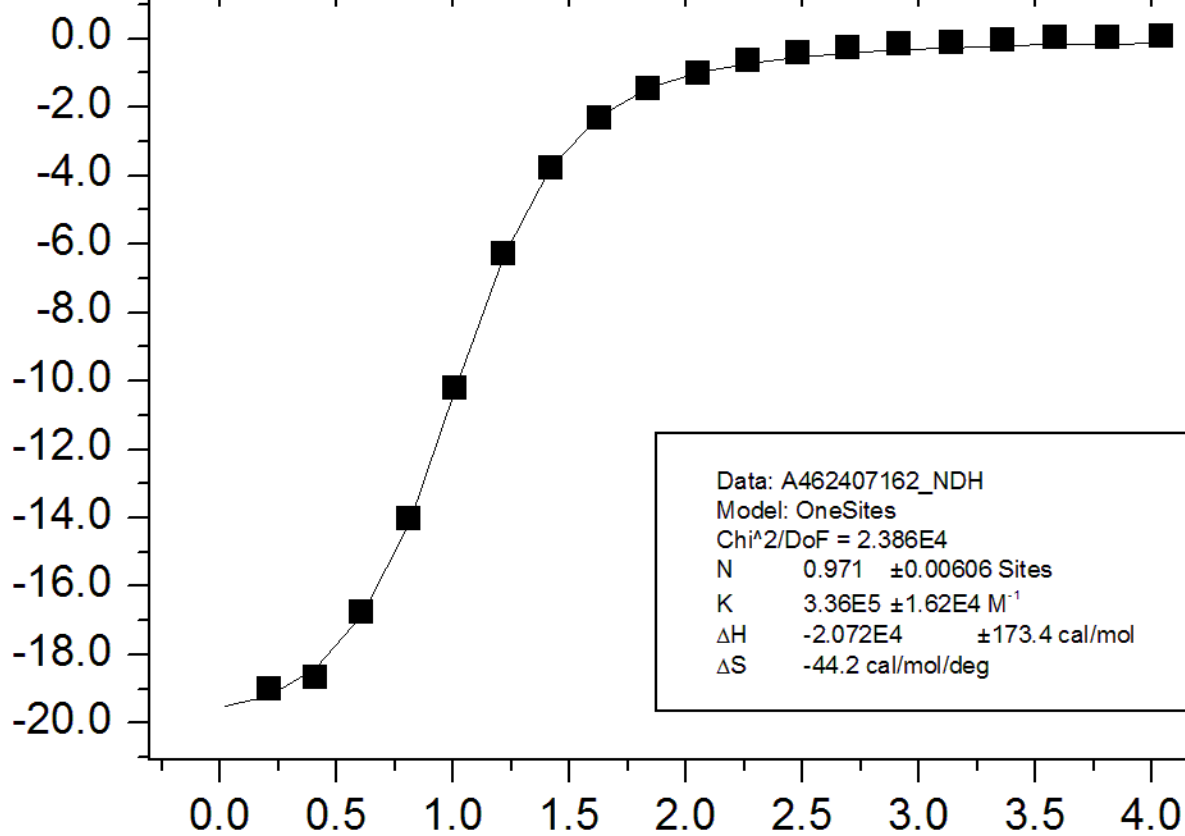

Molar Ratio

Supplement: Supplementary file 5 — ITC data files. [file 41564_2022_1244_MOESM5_ESM.zip › Homologs_SUBMIT/Enterobacter soil (UNC462-4)/Enterobacter soil_itc1.pdf]

Time (min)

0 10 20 30 40 50 60

0.00

$\mu\text{cal/sec}$

$\text{kcal mol}^{-1}$  of injectant

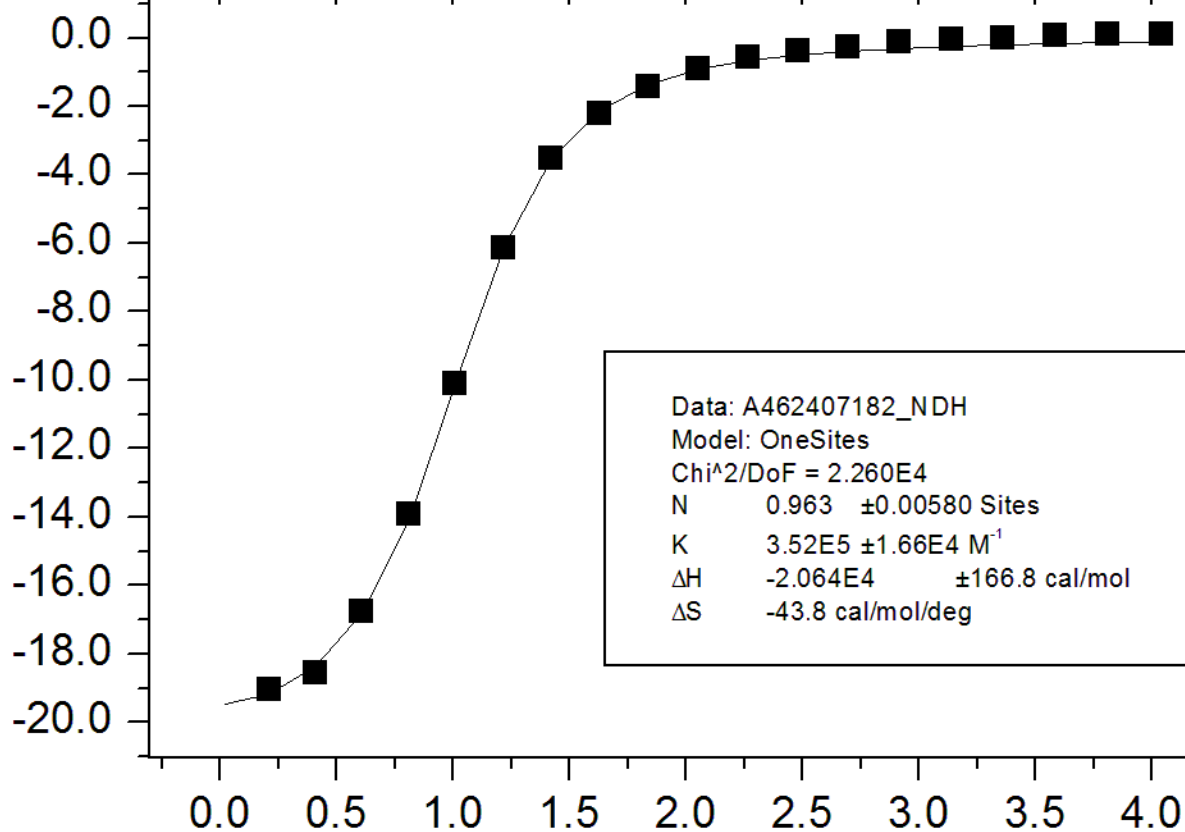

Molar Ratio

Supplement: Supplementary file 5 — ITC data files. [file 41564_2022_1244_MOESM5_ESM.zip › Homologs_SUBMIT/Enterobacter soil (UNC462-4)/Enterobacter soil_itc2.pdf]

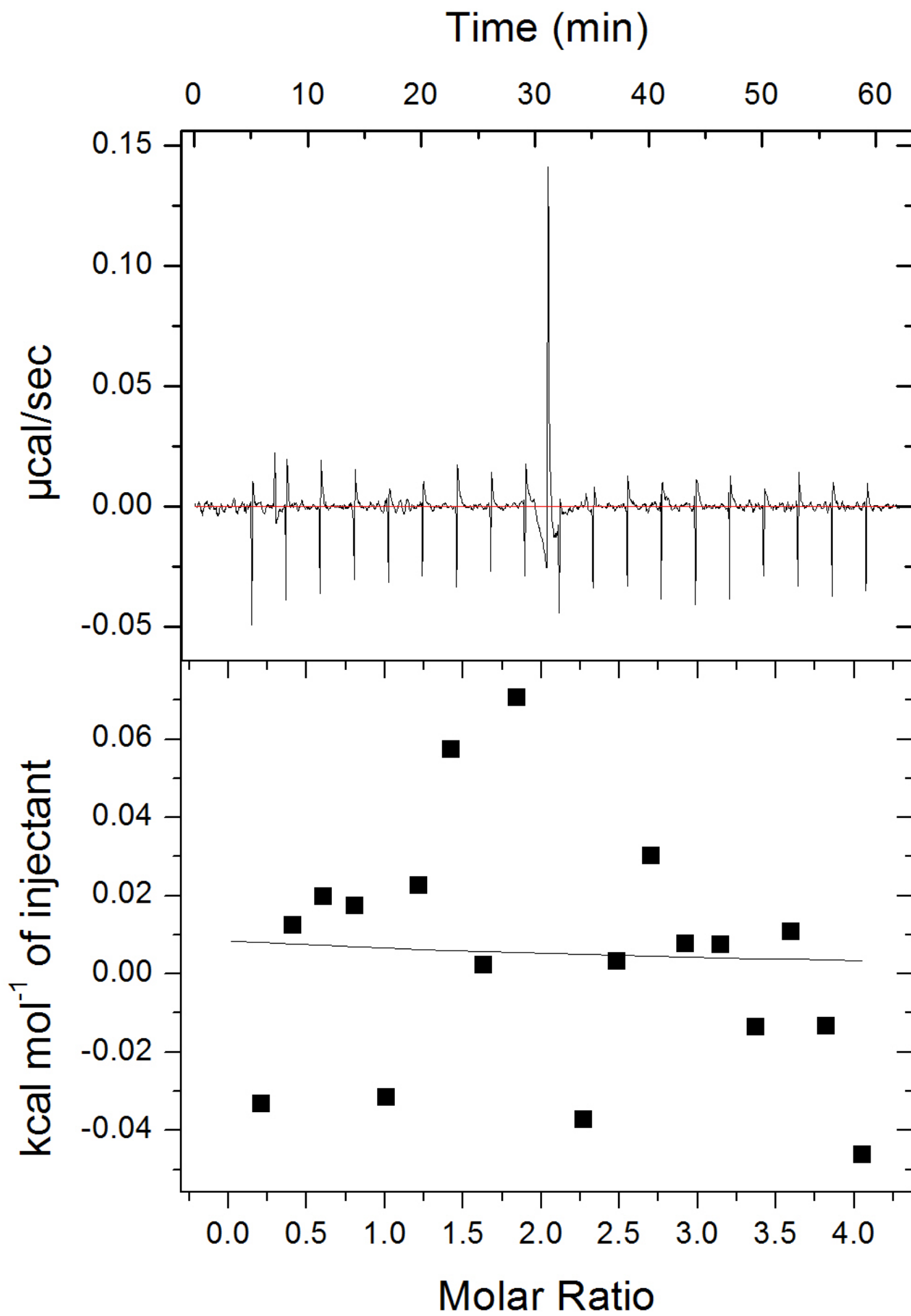

Supplement: Supplementary file 5 — ITC data files. [file 41564_2022_1244_MOESM5_ESM.zip › Homologs_SUBMIT/Variovorax_paradoxus_MarR_50/Variovorax_paradoxus_MarR_50_itc2.PDF]

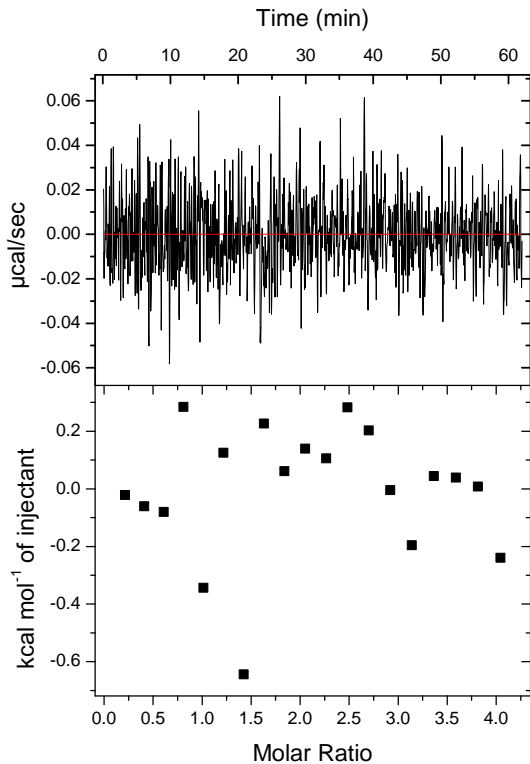

Supplement: Supplementary file 5 — ITC data files. [file 41564_2022_1244_MOESM5_ESM.zip › Homologs_SUBMIT/Variovorax_paradoxus_MarR_50/Variovorax_paradoxus_MarR_50_itc1.PDF]

Time (min)

0 10 20 30 40 50 60

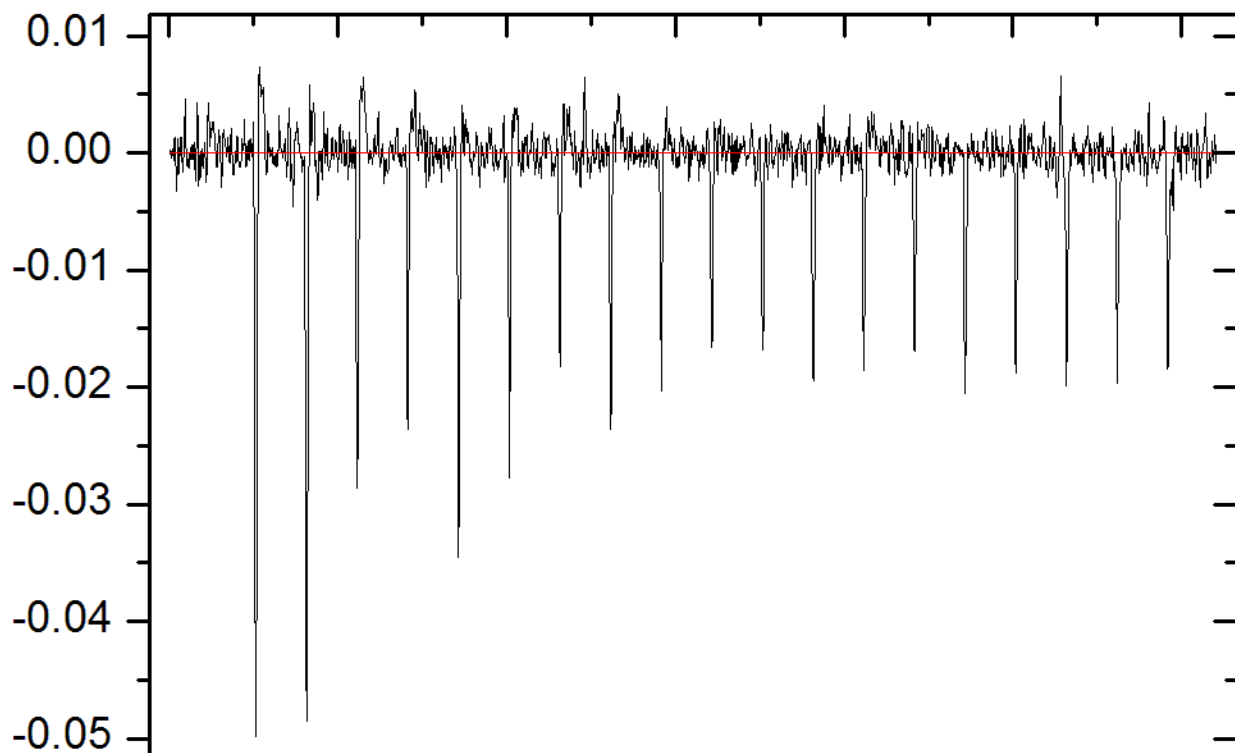

$\mu\text{cal/sec}$

kcal mol<sup>-1</sup> of injectant

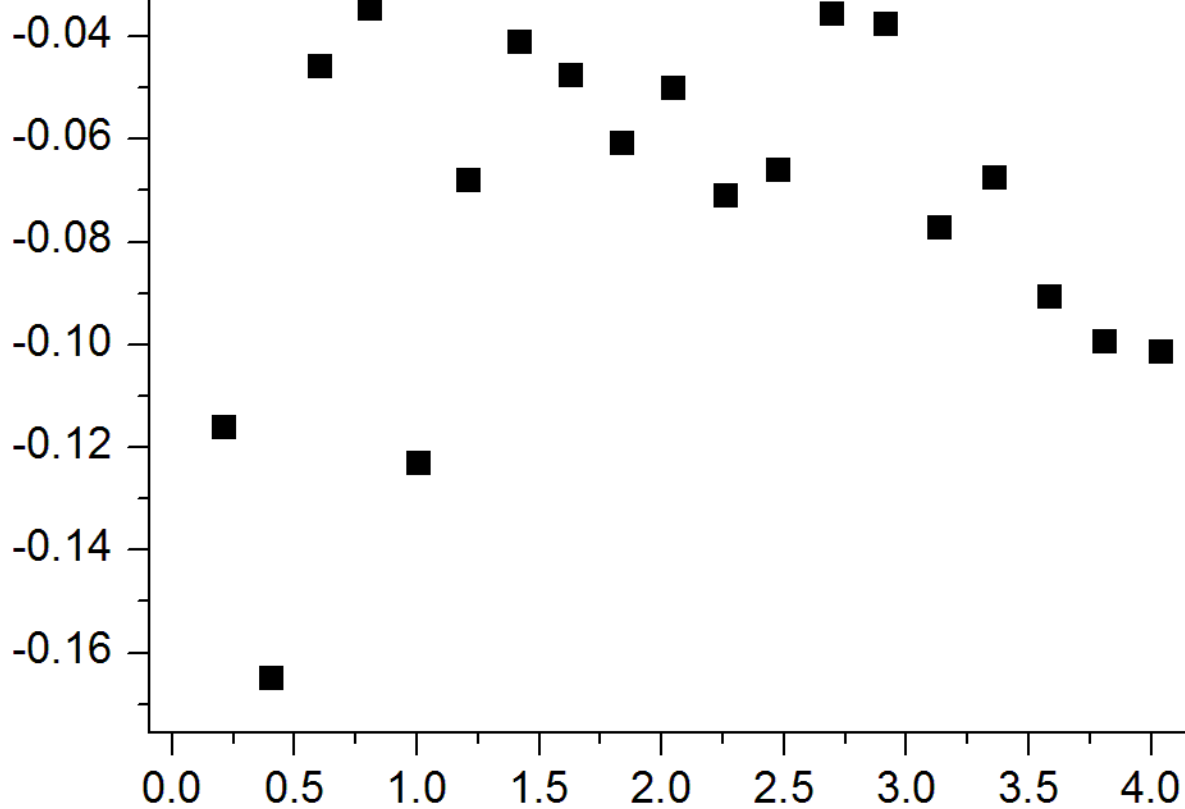

Molar Ratio

Supplement: Supplementary file 5 — ITC data files. [file 41564_2022_1244_MOESM5_ESM.zip › Homologs_SUBMIT/Bradyrhizobium japonicum2 (UNC462-3)/Bradyrhizobium japonicum2_itc1.pdf]

Time (min)

0 10 20 30 40 50 60

$\mu\text{cal/sec}$

0.02  
0.00  
-0.02  
-0.04  
-0.06

$\text{kcal mol}^{-1}$  of injectant

-0.04  
-0.06  
-0.08  
-0.10  
-0.12  
-0.14  
-0.16  
-0.18

Molar Ratio

0.0 0.5 1.0 1.5 2.0 2.5 3.0 3.5 4.0

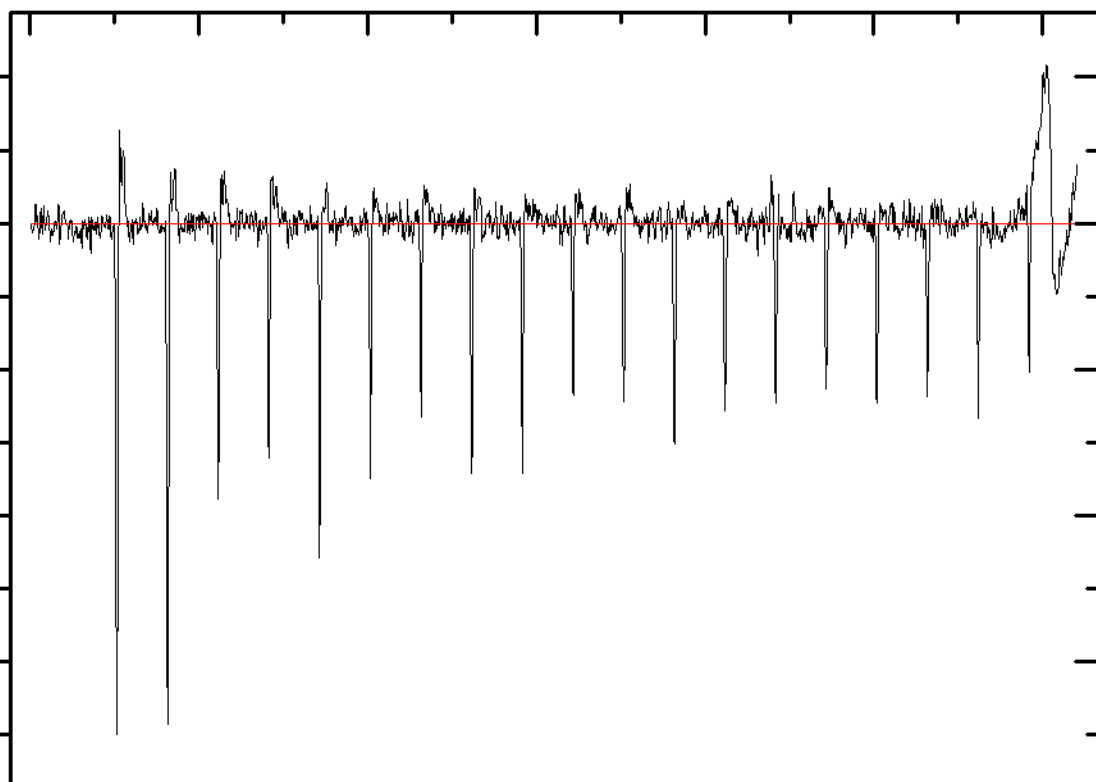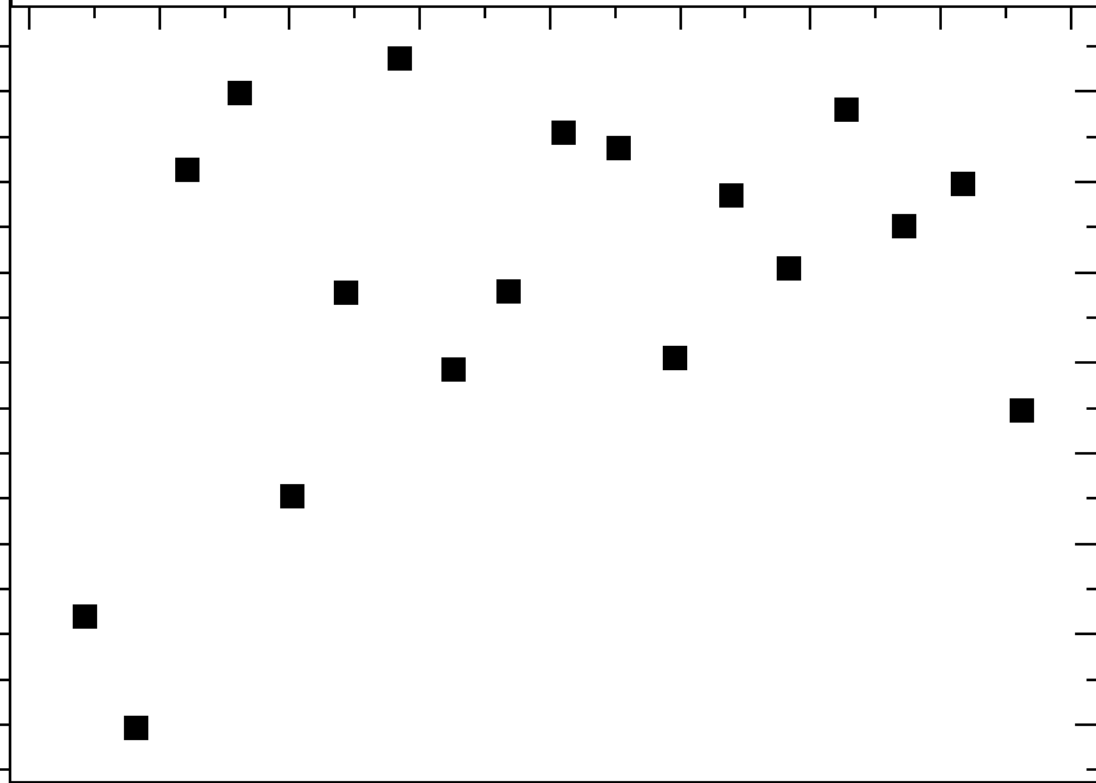

Supplement: Supplementary file 5 — ITC data files. [file 41564_2022_1244_MOESM5_ESM.zip › Homologs_SUBMIT/Bradyrhizobium japonicum2 (UNC462-3)/Bradyrhizobium japonicum2_itc2.pdf]

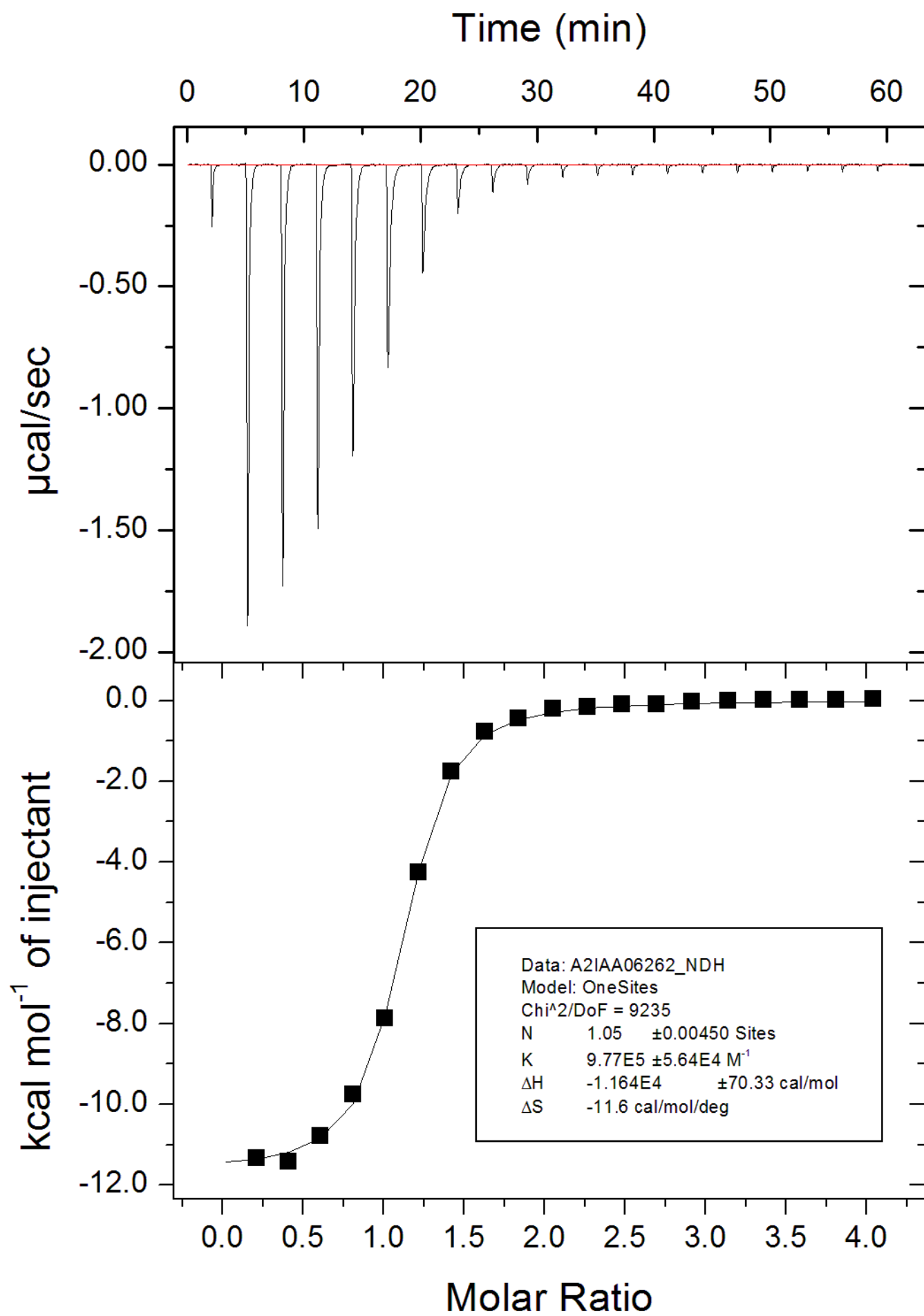

Supplement: Supplementary file 5 — ITC data files. [file 41564_2022_1244_MOESM5_ESM.zip › Homologs_SUBMIT/Bradyrhizobium japonicum1 (UNC462-2)/Bradyrhizobium japonicum1_itc2.pdf]

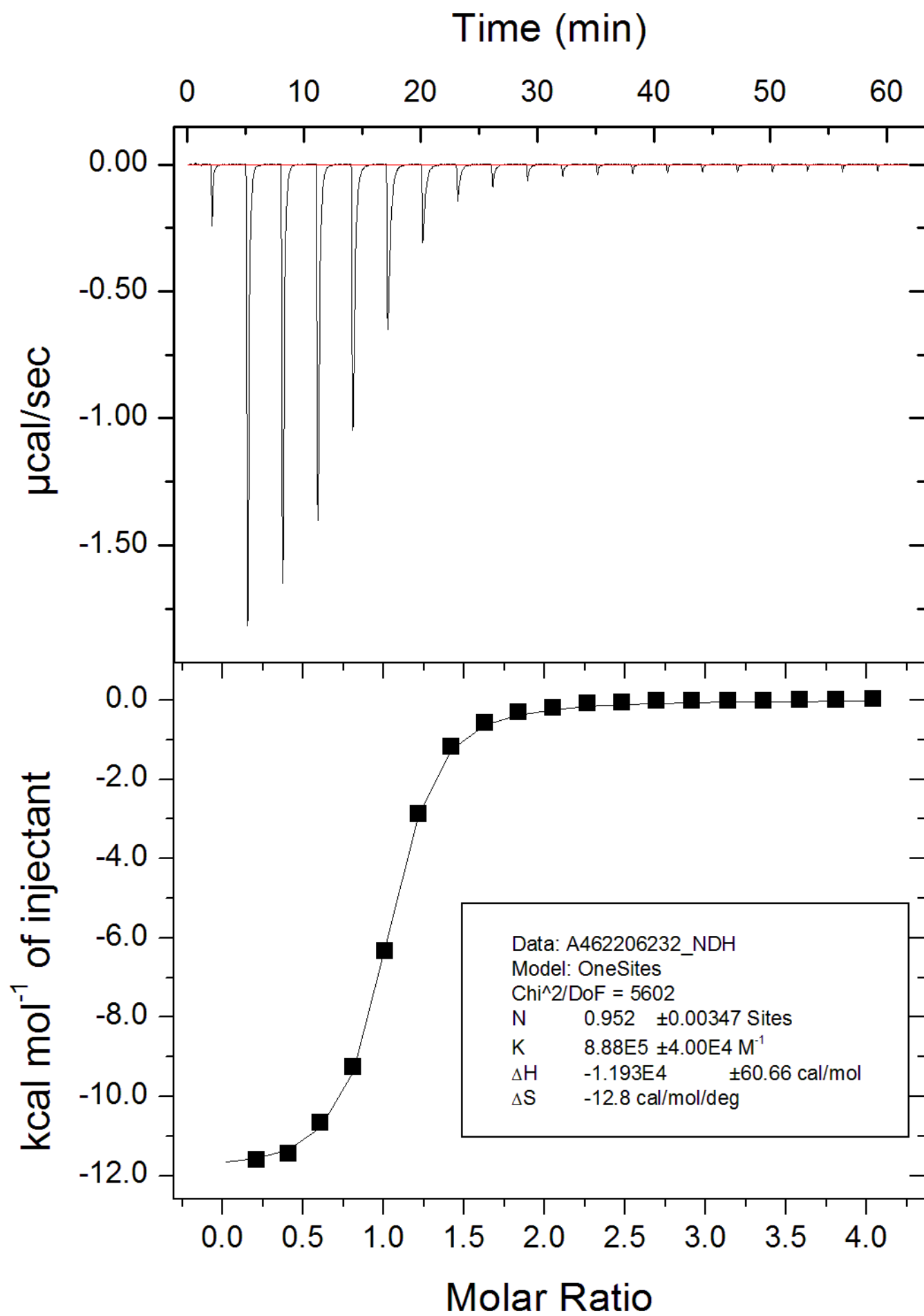

Supplement: Supplementary file 5 — ITC data files. [file 41564_2022_1244_MOESM5_ESM.zip › Homologs_SUBMIT/Bradyrhizobium japonicum1 (UNC462-2)/Bradyrhizobium japonicum1_itc1.pdf]

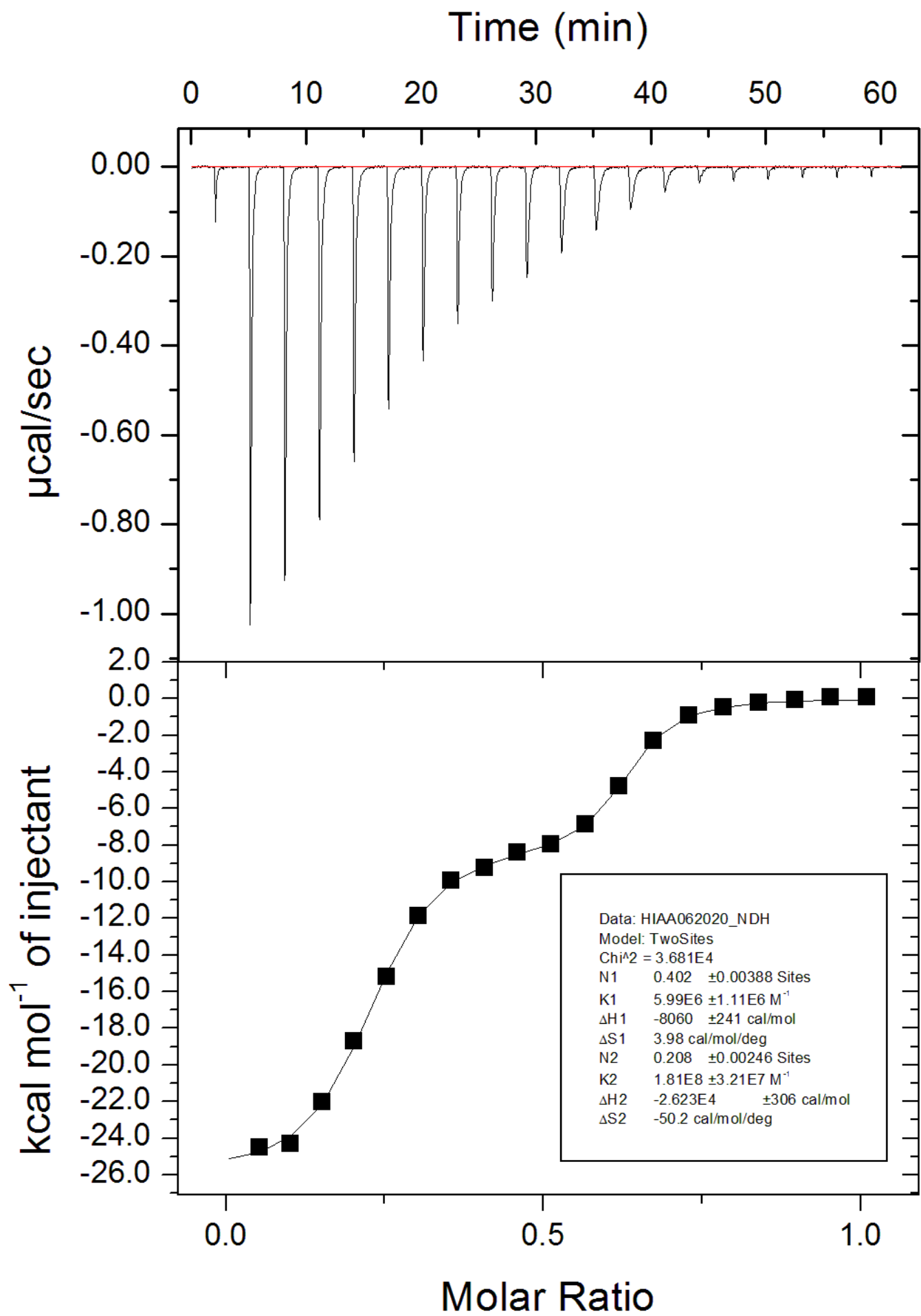

Supplement: Supplementary file 5 — ITC data files. [file 41564_2022_1244_MOESM5_ESM.zip › Homologs_SUBMIT/Ruegaria pomeroyi (3CDH)/Silicibacter pomeroyi_itc2.pdf]

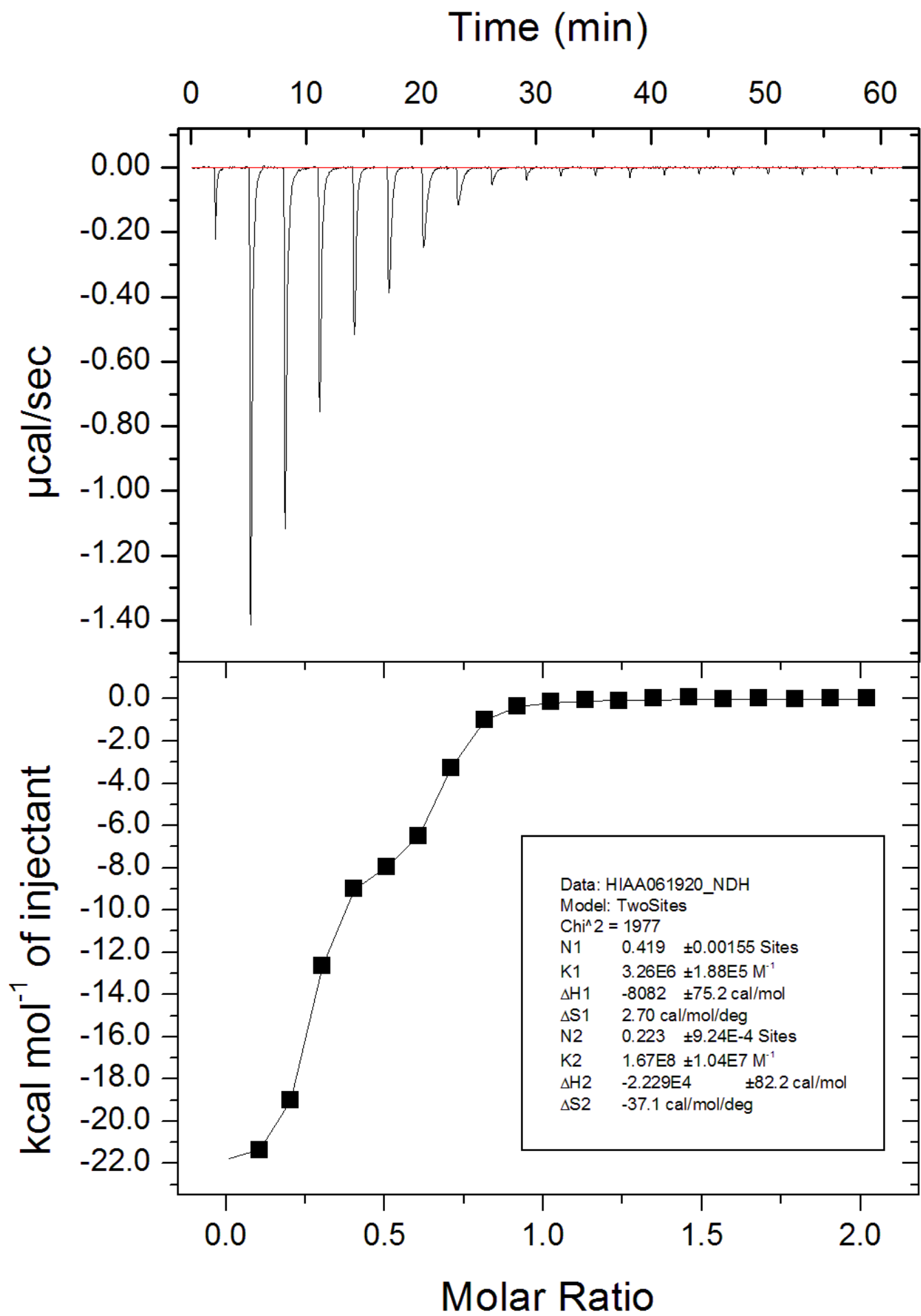

Supplement: Supplementary file 5 — ITC data files. [file 41564_2022_1244_MOESM5_ESM.zip › Homologs_SUBMIT/Ruegaria pomeroyi (3CDH)/Silicibacter pomeroyi_itc1.pdf]
