# Supplementary material for: Diverse MarR bacterial regulators of auxin catabolism in the plant microbiome
Source: Nat Microbiol. 2022 Oct 20;7(11):1817–33. doi: 10.1038/s41564-022-01244-3 (PMC9613470; doi:10.1038/s41564-022-01244-3)
Supplement: Supplementary file 4 — ITC data files. [file 41564_2022_1244_MOESM4_ESM.zip › Variovorax_paradoxus_MarR_73_mutants_IAA_SUBMIT /S28A_IAA/S28A_IAA_itc2.pdf]

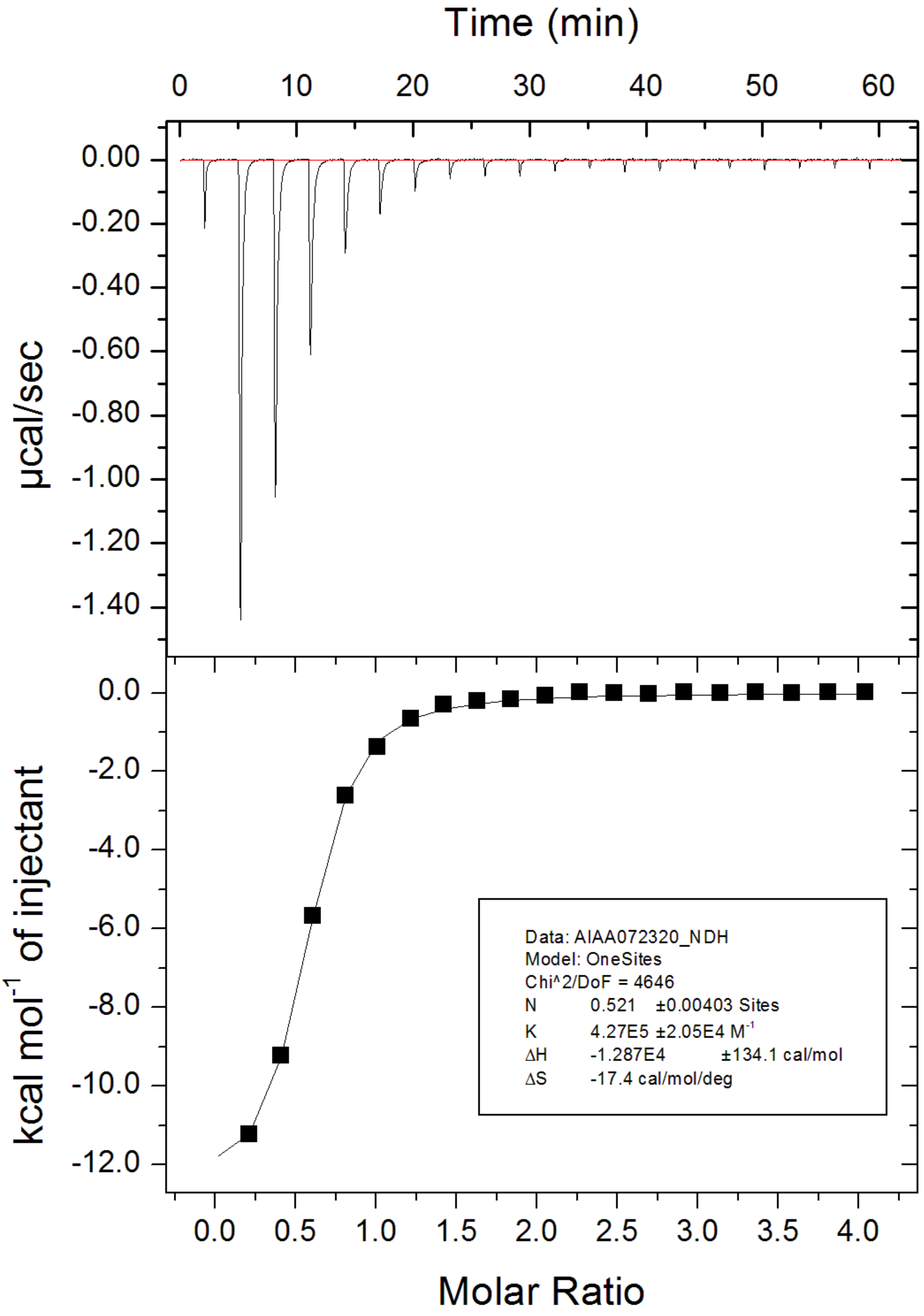
Time (min)
0
10
20
30
40
50
60
0.00
-0.20
-0.40
-0.60
-0.80
-1.00
-1.20
-1.40
µcal/sec
0.0
-2.0
-4.0
-6.0
-8.0
-10.0
-12.0
kcal mol-1 of injectant
Data: AIAA072320_NDH
Model: OneSites
Chi^2/DoF = 4646
N 0.521 ±0.00403 Sites
K 4.27E5 ±2.05E4 M-1
ΔH -1.287E4 ±134.1 cal/mol
ΔS -17.4 cal/mol/deg
0.0
0.5
1.0
1.5
2.0
2.5
3.0
3.5
4.0
Molar Ratio
